# Antibody responses to the RTS,S/AS01_E_ vaccine and *Plasmodium falciparum* antigens after a booster dose within the phase 3 trial in Mozambique

**DOI:** 10.1038/s41541-020-0192-7

**Published:** 2020-06-04

**Authors:** Lina Sánchez, Marta Vidal, Chenjerai Jairoce, Ruth Aguilar, Itziar Ubillos, Inocencia Cuamba, Augusto J. Nhabomba, Nana Aba Williams, Núria Díez-Padrisa, David Cavanagh, Evelina Angov, Ross L. Coppel, Deepak Gaur, James G. Beeson, Sheetij Dutta, Pedro Aide, Joseph J. Campo, Gemma Moncunill, Carlota Dobaño

**Affiliations:** 1 ISGlobal, Hospital Clínic—Universitat de Barcelona, Barcelona, Catalonia Spain; 2 UnivLyon, Université Claude Bernard Lyon 1, 69100 Villeurbanne, France; 3 Centro de Investigação em Saúde de Manhiça (CISM), Maputo, Mozambique; 4 Institute of Immunology & Infection Research and Centre for Immunity, Infection & Evolution, Ashworth Laboratories, School of Biological Sciences, University of Edinburgh, King’s Buildings, Edinburgh, UK; 5 U.S. Military Malaria Vaccine Program, Walter Reed Army Institute of Research (WRAIR), Silver Spring, MD USA; 6 Infection and Immunity Program, Monash Biomedicine Discovery Institute and Department of Microbiology, Monash University, Melbourne, VIC Australia; 7 Malaria Group, International Centre for Genetic Engineering and Biotechnology (ICGEB), New Delhi, India; 8 Laboratory of Malaria and Vaccine Research, School of Biotechnology, Jawaharlal Nehru University, New Delhi, India; 9 Burnet Institute, Melbourne, VIC Australia

**Keywords:** Malaria, Adaptive immunity, Vaccines

## Abstract

The RTS,S/AS01_E_ vaccine has shown consistent but partial vaccine efficacy in a pediatric phase 3 clinical trial using a 3-dose immunization schedule. A fourth-dose 18 months after the primary vaccination was shown to restore the waning efficacy. However, only total IgG against the immunodominant malaria vaccine epitope has been analyzed following the booster. To better characterize the magnitude, nature, and longevity of the immune response to the booster, we measured levels of total IgM, IgG, and IgG_1-4_ subclasses against three constructs of the circumsporozoite protein (CSP) and the hepatitis B surface antigen (HBsAg, also present in RTS,S) by quantitative suspension array technology in 50 subjects in the phase 3 trial in Manhiça, Mozambique. To explore the impact of vaccination on naturally acquired immune responses, we measured antibodies to *P. falciparum* antigens not included in RTS,S. We found increased IgG, IgG1, IgG3 and IgG4, but not IgG2 nor IgM, levels against vaccine antigens 1 month after the fourth dose. Overall, antibody responses to the booster dose were lower than the initial peak response to primary immunization and children had higher IgG and IgG1 levels than infants. Higher anti-Rh5 IgG and IgG_1-4_ levels were detected after the booster dose, suggesting that RTS,S partial protection could increase some blood stage antibody responses. Our work shows that the response to the RTS,S/AS01_E_ booster dose is different from the primary vaccine immune response and highlights the dynamic changes in subclass antibody patterns upon the vaccine booster and with acquisition of adaptive immunity to malaria.

## Introduction

Despite the great reduction in malaria cases in the last 15 years, thanks to the combination of multiple control measures, it is estimated that 219 million malaria cases and 435,000 deaths occurred in 2017, mostly associated with *Plasmodium falciparum*^1^. Importantly, 90% of these deaths concentrated in sub-Saharan Africa and a large proportion occurred in children under 5 years. Owing to the concerning rise of parasite resistance to antimalarial drugs and vector resistance to insecticides^1,2^ and stalling progress in reducing malaria since 2016^1,2^, integration of a malaria vaccine with other preventive measures will be a useful addition to control disease burden in the future.

Currently, the pre-erythrocytic RTS,S/AS01_E_ vaccine is the most advanced, having shown consistent but partial vaccine efficacy (VE) that wanes over time and is less effective in infants compared to children^3^. RTS,S/AS01E contains a fusion protein including the central tandem repeat (NANP) and the C-terminal (C-term) regions of the *P. falciparum* circumsporozoite protein (CSP), and the hepatitis B virus surface antigen (HBsAg). It is expressed together with HBsAg, and injected in combination with the AS01 adjuvant system^4^. The vaccine was tested in a phase 3 clinical trial of a 3-dose immunization schedule (month [M] 0, M1 and M2) with a fourth dose 18 months after primary vaccination (M20)^3^, with the booster dose partly restoring the waning VE. Specifically, VE for the 3-dose immunization schedule was 35.2% in children and 20.3% in infants up to M32 of the study, but VE waned over time with a VE of 16.1 and 7.6%, respectively, when considering only the period from M20 to M32. In children and infants who received the booster dose, waning VE was restored to overall levels of 43.9 and 27.8%, respectively^3^. In order to understand why protection offered by RTS,S is suboptimal and continue efforts to improve it, there is a need to decipher the mechanisms of protection elicited by the vaccine. It has been shown that antibody levels are involved in the vaccine-induced immunity, but they do not fully explain the protective effect of the vaccine^5,6^. Thus far, the study of antibody response in trials performed in endemic areas has been largely focused on IgG levels against the NANP repeat region of CSP, with the exception of our previous work assessing more generally subclass responses to NANP and to other antigens after primary vaccination in the phase 3 trial^7–9^.

Characterizing responses by other antibody isotypes, subclasses, and responses to different epitopes may provide in depth understanding of the immune response to the vaccine and the mode of action. Antibody levels are not the sole means to determine vaccine mechanisms of action. Characteristics like the balance between isotypes or subclasses of the antibodies are important because of their varying effector functions^10^. For instance, some IgG subclasses act as cytophilic while others have non-cytophilic functions^10^, influencing the roles of Fc-mediated functions such as complement fixation and phagocytosis^11^. Determining which type of response is detrimental or beneficial could further inform which responses could be modified to enhance the efficacy of the vaccine.

The epitope specificity of the antibody response is also relevant. There is clear evidence that NANP is related to VE^6^ but other regions could also mediate protection. Avidity of IgG to the CSP C-term has been associated with protection in African children^12^, and C-term and not the NANP-repeat-specific antibodies have been reported to be the main mediators of phagocytic activity in naive adults^13^. Furthermore, antibodies to both C-term and NANP-repeat can mediate complement fixation in children, suggesting both regions are important for functional activity^14,15^.

Additionally, studying the response to *P. falciparum* blood stage antigens not present in the vaccine is relevant to determine the effect of the vaccine on naturally acquired immunity (NAI), developed from continuous parasite exposure. It has been hypothesized that vaccination could (1) decrease NAI by reducing the exposure to the parasite, which could mean individuals are left vulnerable in the long term due to the waning efficacy of the vaccine^3^, as predicted for other malaria prevention tools^16^, or (2) increase NAI by allowing subclinical exposure to the parasite due to the partial efficacy of the vaccine^9,17^.

Here, we used samples from the phase 3 trial at the time of the booster dose (M20) and onwards from a subgroup of subjects in Manhiça, Mozambique, to characterize the effect of the RTS,S/AS01_E_ booster dose on different antibody responses. We evaluated total IgM, IgG and IgG_1-4_ subclasses to vaccine and vaccine-unrelated *P. falciparum* blood stage antigens. Data were combined with those from the primary vaccine response previously assessed^7–9^ to display the kinetics from baseline (M0) until M32.

## Results

### Short- and long-term booster immunogenicity

**Fig. 1:**
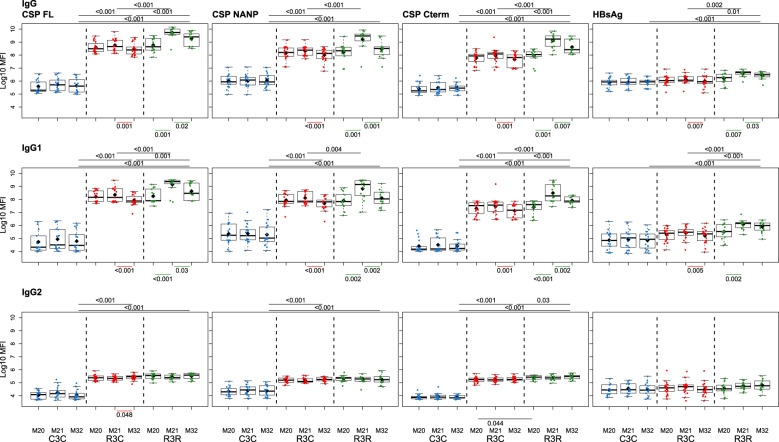
RTS,S/AS01_E_ booster and long-term immunogenicity against vaccine antigens: total IgG, IgG1-2 subclasses for CSP constructs and HBsAg at month (M) 20, 21, and 32 for RTS,S/AS01 vaccinees with (R3R) and without (R3C) booster, and comparator (C3C). Boxplots with medians, interquartile ranges (IQR), upper whisker as the smallest between maximum × value and Q3 + 1.5*IQR, lower whisker as the largest between minimum × value and Q1 – 1.5*IQR, and log_10_(geometric mean(MFI)) (diamond). Non-parametric tests were used to compare the booster response (M20 vs. M21) and the long-term immunogenicity (M21 vs. M32), as well as to compare the R3C and R3R groups at each timepoint. Only *p*-values < 0.05 after adjustment for multiple testing are shown. The *y*-axis is in logarithm 10 scale. R3R (green): three doses of RTS,S/AS01_E_ and a RTS,S/AS01_E_ booster. R3C (red): three doses of RTS,S/AS01_E_ and a comparator booster. C3C (blue): three doses of a comparator vaccine and a comparator booster.

**Fig. 2:**
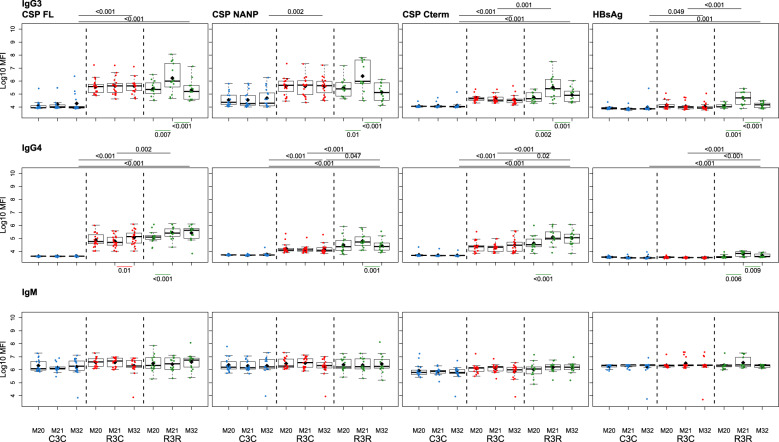
RTS,S/AS01_E_ booster and long-term immunogenicity against vaccine antigens: IgG3-4 subclasses and IgM for CSP constructs and HBsAg at month (M) 20, 21, and 32 for RTS,S/AS01 vaccinees with (R3R) and without (R3C) booster, and comparator (C3C). Boxplots with medians, interquartile ranges (IQR), upper whisker as the smallest between maximum × value and Q3 + 1.5*IQR, lower whisker as the largest between minimum × value and Q1 – 1.5*IQR, and log_10_(geometric mean(MFI)) (diamond). Non-parametric tests were used to compare the booster response (M20 vs. M21) and the long-term immunogenicity (M21 vs. M32), as well as to compare the R3C and R3R groups at each timepoint. Only *p*-values < 0.05 after adjustment for multiple testing are shown. The *y-*axis is in logarithm 10 scale. R3R (green): three doses of RTS,S/AS01_E_ and a RTS,S/AS01_E_ booster. R3C (red): three doses of RTS,S/AS01E and a comparator booster. C3C (blue): three doses of a comparator vaccine and a comparator booster.

The RTS,S/AS01_E_ booster dose increased IgG, IgG1, IgG3, and IgG4 levels against all vaccine antigens 1 month (M21) after its administration (M20), but it did not increase IgG2 nor IgM levels (Figs. 1 and 2; and Supplementary Table 1). The increase in antibody levels was significant both when comparing the levels pre-boost at M20 and M21 of the same individual, and when comparing the levels at post-boost M21 of the RTS,S booster group (R3R) to those of the individuals who did not receive a booster (R3C), except for IgG3 CSP NANP and IgG3 CSP full length (FL) for the latter comparison. At M21, the highest levels were against FL CSP, followed by the CSP NANP region, the CSP C-term and HBsAg. The predominant subclass was IgG1 followed by IgG3, then lower levels of IgG2 and least for IgG4.

Longer-term immunogenicity was measured 1 year after the administration of the booster (M32). IgG and IgG1 (but not IgG3) levels against vaccine antigens in the R3R group remained above the R3C group, except for IgG1 NANP (Figs. 1 and 2; and Supplementary Table 1). Similar to the pattern at M21, IgG2 and IgM levels were not higher in R3R at M32. For IgG4, levels were significantly higher in R3R compared to R3C only for CSP C-term and HBsAg. In comparison to the group that did not receive any RTS,S dose (C3C), the R3R and R3C groups levels at M32 remained higher for most antigens and IgG subclasses, except HBsAg IgG2 and IgG3, and NANP IgG3.

### Antibody kinetics through the entire study follow-up

**Fig. 3:**
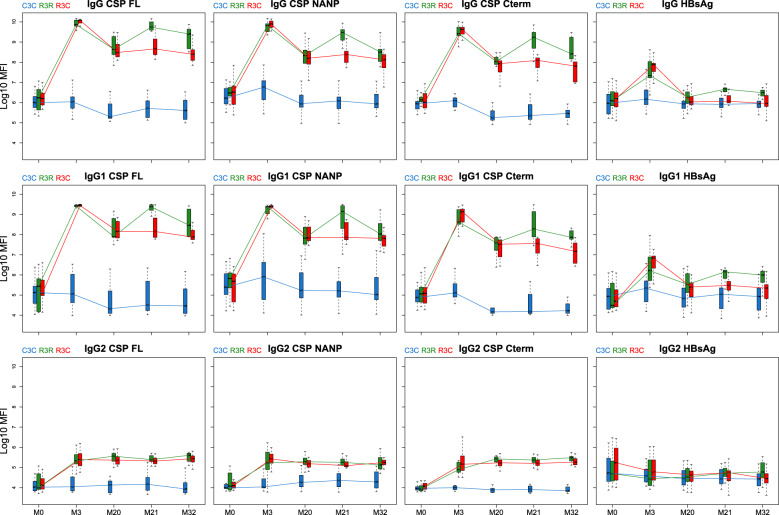
Antibody responses against vaccine antigens for months (M) 0, 3, 20, 21, and 32 for IgG, IgG1, and IgG2. Boxplots with median, interquartile ranges (IQR), upper whisker as the smallest between maximum × value and Q3 + 1.5*IQR, and lower whisker as the largest between minimum × value and Q1 – 1.5*IQR. The *y*-axis is in logarithm 10 scale. Data from months 0 and 3 were obtained from a previous study in the same individuals^7^, thus a batch effect might be present. R3R (green): three doses of RTS,S/AS01_E_ and a RTS,S/AS01_E_ booster at month 20. R3C (red): three doses of RTS,S/AS01_E_ and a comparator booster. C3C (blue): three doses of a comparator vaccine and a comparator booster.

**Fig. 4:**
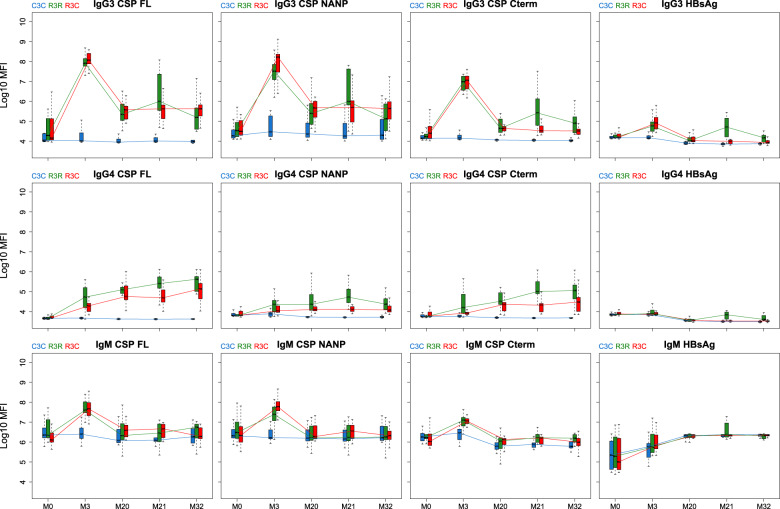
Antibody responses against vaccine antigens for months (M) 0, 3, 20, 21, and 32 for IgG3, IgG4 and IgM. Boxplots with median, interquartile ranges (IQR), upper whisker as the smallest between maximum × value and Q3 + 1.5*IQR, and lower whisker as the largest between minimum × value and Q1 – 1.5*IQR. The *y*-axis is in logarithm 10 scale. Data from months 0 and 3 were obtained from a previous study in the same individuals [7], thus a batch effect might be present. R3R (green): three doses of RTS,S/AS01_E_ and a RTS,S/AS01_E_ booster at month 20. R3C (red): three doses of RTS,S/AS01_E_ and a comparator booster. C3C (blue): three doses of a comparator vaccine and a comparator booster.

When comparing the booster response (M21) to the primary vaccination response (M3), the group that received the booster had a lower peak in IgG, IgG1 and IgG3 levels after the booster than after primary vaccination (Figs. 3 and 4; and Supplementary Figs. 1 and 2). In contrast, IgG4 levels against CSP constructs showed higher levels after the booster dose than after primary vaccination, and overall levels increased in time. The opposite happened with HBsAg where IgG4 levels decreased with time and were higher at M3 than at M21. Although primary vaccination increased IgG2 and IgM levels, the booster dose did not increase them.

The decrease in anti-CSP levels from primary vaccination to M20 was larger for IgG3 than for IgG and IgG1; the mean decreases for anti-NANP IgG and IgG1 was around 1.5 log_10_ median fluorescent intensity (MFI) while for IgG3 it was around 2.5 log_10_MFI. IgG2 levels remained more stable after the primary vaccination and did not increase or decrease vastly after M3. Remarkably, IgG4 levels against CSP FL and C-term at M20 were slightly higher than levels at M3 although this effect was not observed in the C3C group.

The levels of IgG and IgG_1-4_ to CSP in RTS,S/AS01_E_ vaccination groups (R3), with or without booster, were higher for most post-vaccination time points than the levels in the comparator group. IgM levels were higher only at M3 in the R3 groups compared to the C3C group.

### Factors affecting immunogenicity

#### Age

Children who received RTS,S/AS01_E_ either with or without a booster had higher IgG and IgG1 levels against CSP antigens than infants throughout the study period (Supplementary Figs. 1–6 and Supplementary Table 2). Without booster, IgG3 levels to NANP and FL CSP, but not C-term, were higher in children than infants. In contrast, we did not detect differences in IgG3 levels between age groups after booster immunization. Most of the differences observed were not statistically significant but there were consistent patterns, e.g., for the same isotype/subclass and antigen, levels were lower in infants than children and all comparisons were *p* < 0.05 before adjustment for multiple testing. We did not detect a significant influence of age on IgG2, IgG4, or IgM levels in any group after the booster, except for NANP IgG4 levels in the R3R group that were higher in children. Likewise, we did not detect significant differences in antibody levels against HBsAg between age groups.

#### Malaria episodes

**Fig. 5:**
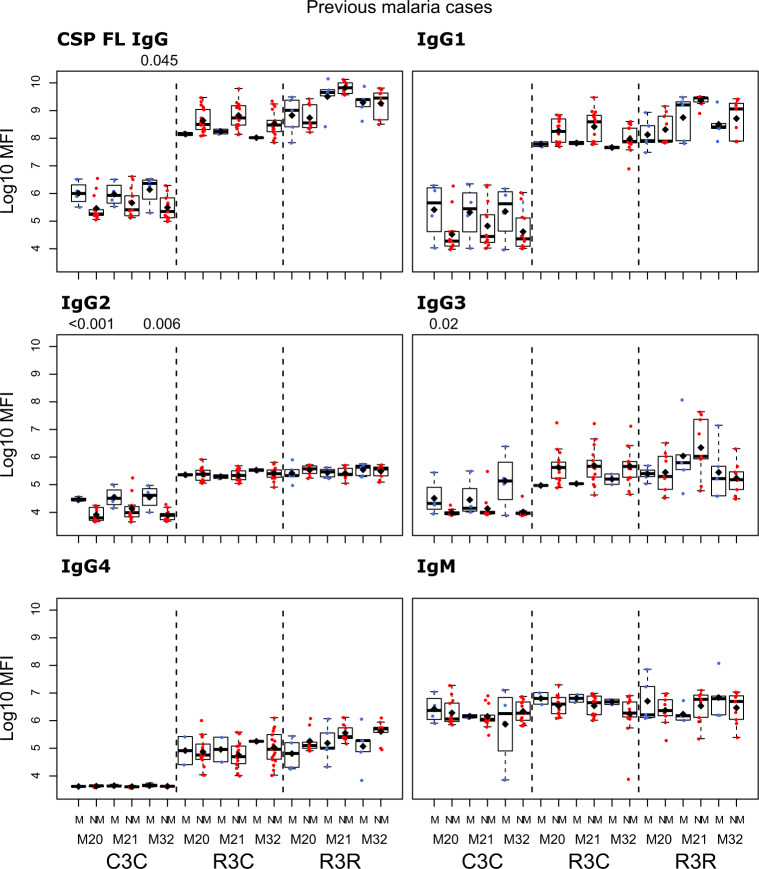
Immunogenicity for CSP FL stratified by previous clinical malaria: total IgG, IgG1-4 subclasses and IgM at month (M)20, 21, and 32 for RTS,S/AS01 vaccinees with (R3R) and without (R3C) booster, and comparator (C3C). Stratified analysis by malaria cases before M20, subjects who presented with clinical malaria (M = blue) and subjects without malaria (NM = red). Boxplots with medians, interquartile ranges (IQR), upper whisker as the smallest between maximum × value and Q3 + 1.5*IQR, lower whisker as the largest between minimum × value and Q1 – 1.5*IQR, and log_10_(geometric mean(MFI)) (diamond). Non-parametric tests were used to compare levels with or without clinical malaria (M vs. NM). *p*-values were adjusted for multiple comparisons, but none was significant. Only *p*-values < 0.05 before adjustment are shown. The *y*-axis is in logarithm 10 scale. R3R: three doses of RTS,S/AS01_E_ and a RTS,S/AS01_E_ booster. R3C: three doses of RTS,S/AS01_E_ and a comparator booster. C3C: three doses of a comparator vaccine and a comparator booster.

**Fig. 6:**
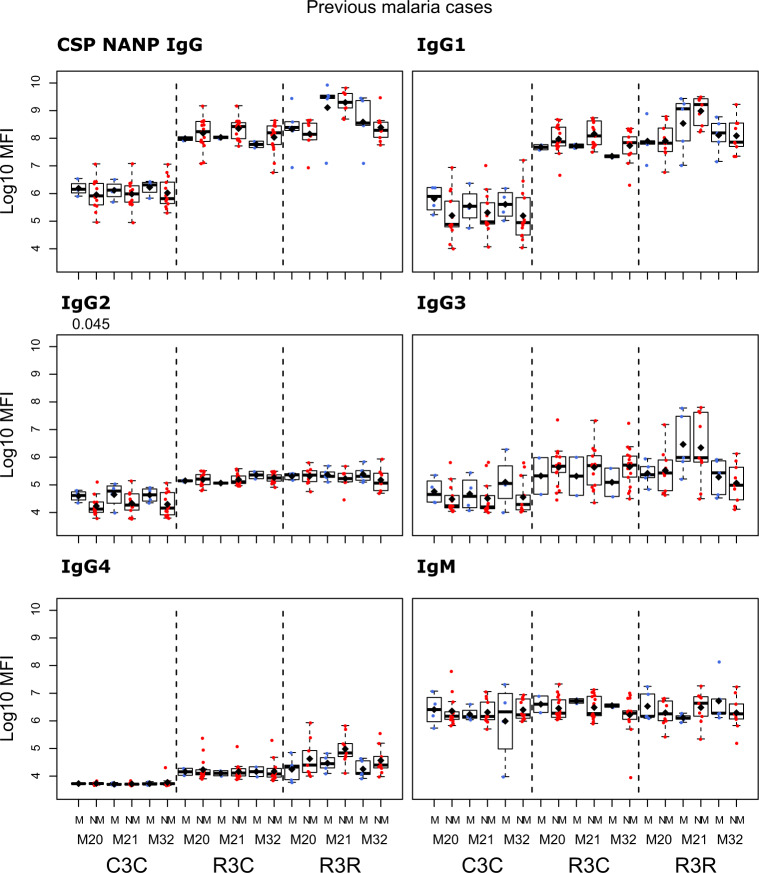
Immunogenicity for CSP NANP stratified by previous clinical malaria: total IgG, IgG1-4 subclasses and IgM at month (M)20, 21, and 32 for RTS,S/AS01 vaccinees with (R3R) and without (R3C) booster, and comparator (C3C). Stratified analysis by malaria cases before M20, subjects who presented with clinical malaria (M = blue) and subjects without malaria (NM = red). Boxplots with medians, interquartile ranges (IQR), upper whisker as the smallest between maximum × value and Q3 + 1.5*IQR, lower whisker as the largest between minimum × value and Q1 – 1.5*IQR, and log_10_(geometric mean(MFI)) (diamond). Non-parametric tests were used to compare levels with or without clinical malaria (M vs. NM). *p*-values were adjusted for multiple comparisons, but none was significant. Only *p*-values < 0.05 before adjustment are shown. The *y*-axis is in logarithm 10 scale. R3R: three doses of RTS,S/AS01_E_ and a RTS,S/AS01_E_ booster. R3C: three doses of RTS,S/AS01_E_ and a comparator booster. C3C: three doses of a comparator vaccine and a comparator booster.

**Fig. 7:**
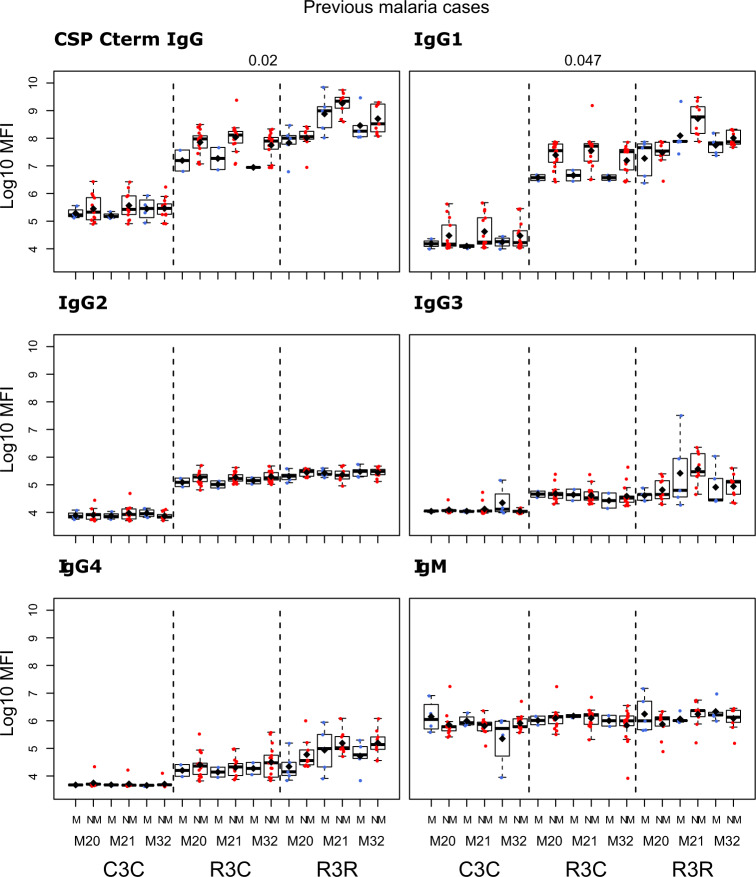
Immunogenicity for CSP C-term stratified by previous clinical malaria: total IgG, IgG1-4 subclasses and IgM at month (M)20, 21, and 32 for RTS,S/AS01 vaccinees with (R3R) and without (R3C) booster, and comparator (C3C). Stratified analysis by malaria cases before M20, subjects who presented with clinical malaria (M = blue) and subjects without malaria (NM = red). Boxplots with medians, interquartile ranges (IQR), upper whisker as the smallest between maximum × value and Q3 + 1.5*IQR, lower whisker as the largest between minimum × value and Q1 – 1.5*IQR, and log_10_ (geometric mean(MFI)) (diamond). Non-parametric tests were used to compare levels with or without clinical malaria (M vs. NM). *p*-values were adjusted for multiple comparisons, but none was significant. Only *p*-values < 0.05 before adjustment are shown. The *y*-axis is in logarithm 10 scale. R3R: three doses of RTS,S/AS01_E_ and a RTS,S/AS01_E_ booster. R3C: three doses of RTS,S/AS01_E_ and a comparator booster. C3C: three doses of a comparator vaccine and a comparator booster.

We compared the antibody levels at M20, M21 and M32 in individuals who had either presented or not with clinical malaria before M20 (Figs. 5–7 and Supplementary Table 3). None of the comparisons were statistically significant after adjusting for multiple testing. At M20 we did not detect any significant difference between individuals who presented or not with prior clinical malaria in the RTS,S vaccinees. In the R3R group at M21 there was a pattern for lower anti-CSP FL and anti-C-term IgG, IgG1, IgG3, IgG4 and IgM levels, and anti-NANP IgG4 levels (*p* < 0.05 before adjustment) in individuals who had clinical malaria. For the R3C group at M21, individuals who presented with clinical malaria before M20 had lower anti-CSP IgG and IgG1 mean levels against CSP antigens, and lower IgG3 levels against CSP FL (*p* < 0.05 before adjustment). In contrast, IgM levels were higher in plasma from previous malaria cases but this was not statistically significant. In the C3C group, IgG and IgG_1-3_ to FL CSP and NANP were higher in the subjects with previous malaria cases but this difference was not statistically significant after adjusting for multiple testing. Lower levels of IgG, IgG1, and IgG3 to HBsAg were also observed in R3C at M20, M21, and M32 in the previous malaria cases (Supplementary Fig. 7), but these differences were not statistically significant.

**Fig. 8:**
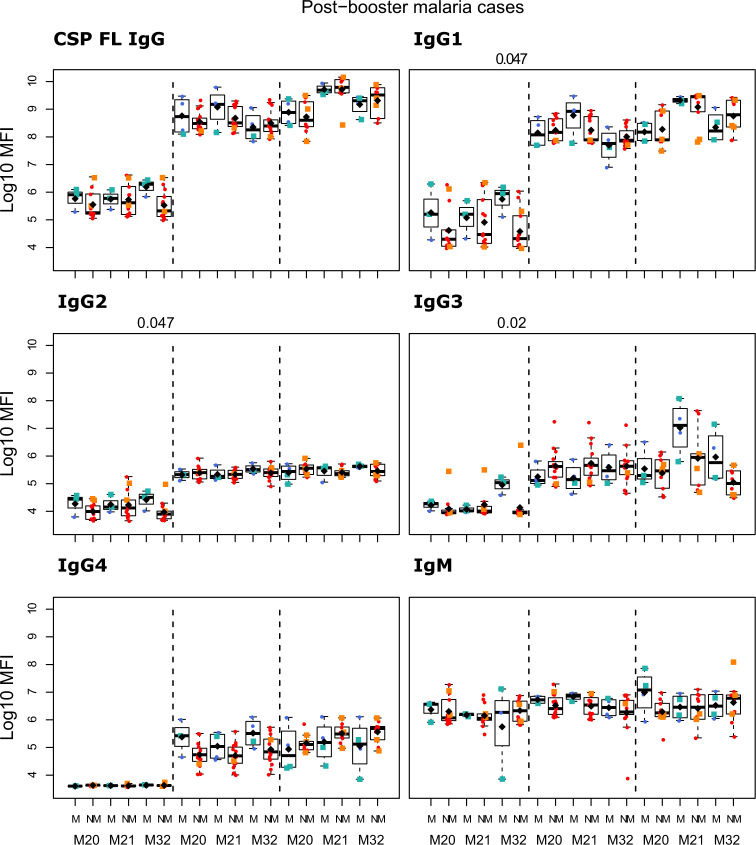
Immunogenicity stratified by clinical malaria after M21: total IgG, IgG1-4 subclasses and IgM for CSP FL at month (M) 20, 21, and 32 for RTS,S/AS01 vaccinees with (R3R) and without (R3C) booster, and comparator (C3C). Stratified analysis by malaria after M21, subjects who presented with clinical malaria (M = blue) and subjects without malaria (NM = red). Subjects who presented with clinical malaria before M20 are represented with green and orange squares. Boxplots with medians, interquartile ranges (IQR), upper whisker as the smallest between maximum × value and Q3 + 1.5*IQR, lower whisker as the largest between minimum × value and Q1 – 1.5*IQR, and log_10_ (geometric mean(MFI)) (diamond). Non-parametric tests were used to compare levels with or without clinical malaria (NM vs. M). *p*-values were adjusted for multiple comparisons, but none was significant. Only *p*-values < 0.05 before adjustment are shown. The *y*-axis is in logarithm 10 scale. R3R: three doses of RTS,S/AS01_E_ and a RTS,S/AS01_E_ booster. R3C: three doses of RTS,S/AS01_E_ and a comparator booster. C3C: three doses of a comparator vaccine and a comparator booster.

**Fig. 9:**
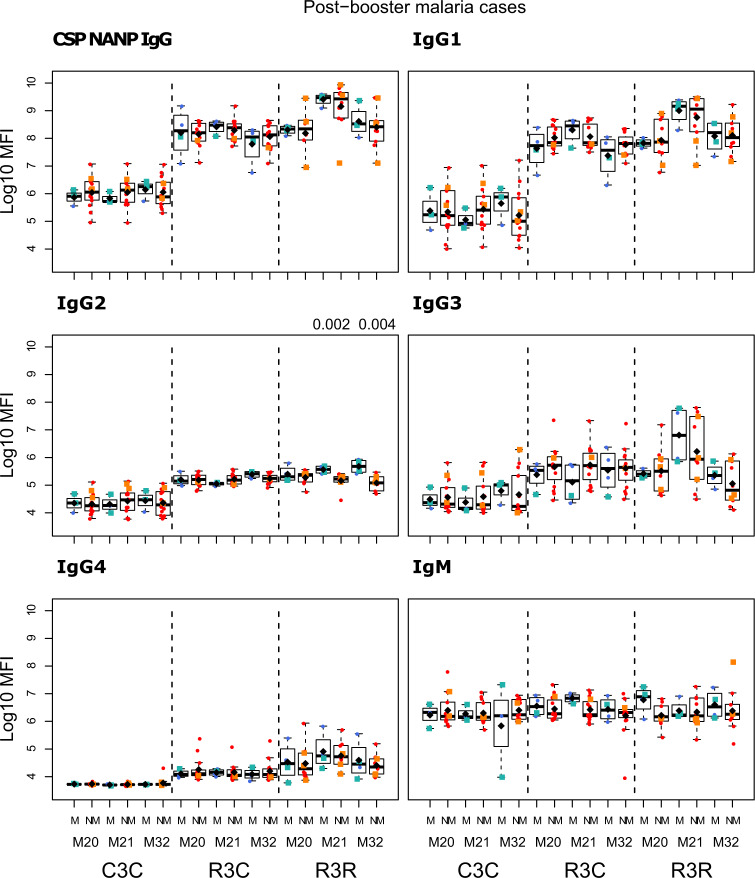
Immunogenicity stratified by clinical malaria after M21: total IgG, IgG1-4 subclasses and IgM for CSP NANP at month (M) 20, 21, and 32 for RTS,S/AS01 vaccinees with (R3R) and without (R3C) booster, and comparator (C3C). Stratified analysis by malaria after M21, subjects who presented with clinical malaria (M = blue) and subjects without malaria (NM = red). Subjects who presented with clinical malaria before M20 are represented with green and orange squares. Boxplots with medians, interquartile ranges (IQR), upper whisker as the smallest between maximum × value and Q3 + 1.5*IQR, lower whisker as the largest between minimum × value and Q1 – 1.5*IQR, and log_10_(geometric mean(MFI)) (diamond). Non-parametric tests were used to compare levels with or without clinical malaria (NM vs. M). *p*-values were adjusted for multiple comparisons, but none was significant. Only *p*-values < 0.05 before adjustment are shown. The *y*-axis is in logarithm 10 scale. R3R: three doses of RTS,S/AS01_E_ and a RTS,S/AS01_E_ booster. R3C: three doses of RTS,S/AS01_E_ and a comparator booster. C3C: three doses of a comparator vaccine and a comparator booster.

**Fig. 10:**
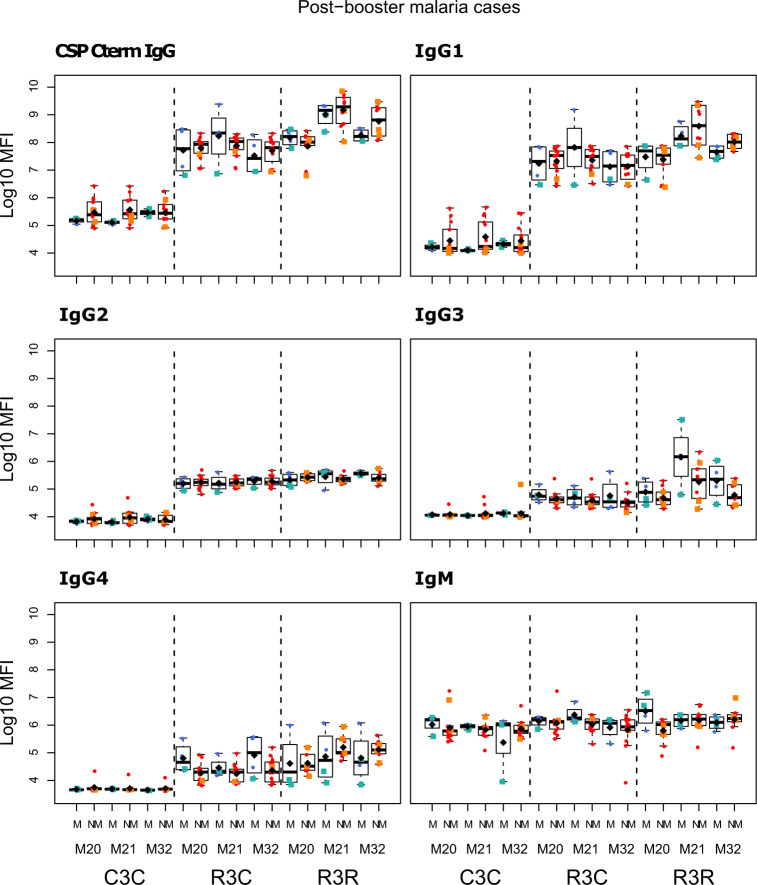
Immunogenicity stratified by clinical malaria after M21: total IgG, IgG1-4 subclasses and IgM for CSP C-term at month (M) 20, 21, and 32 for RTS,S/AS01 vaccinees with (R3R) and without (R3C) booster, and comparator (C3C). Stratified analysis by malaria after M21, subjects who presented with clinical malaria (M = blue) and subjects without malaria (NM = red). Subjects who presented with clinical malaria before M20 are represented with green and orange squares. Boxplots with medians, interquartile ranges (IQR), upper whisker as the smallest between maximum × value and Q3 + 1.5*IQR, lower whisker as the largest between minimum × value and Q1 – 1.5*IQR, and log_10_(geometric mean(MFI)) (diamond). Non-parametric tests were used to compare levels with or without clinical malaria (NM vs. M). *p*-values were adjusted for multiple comparisons, but none was significant. Only *p*-values < 0.05 before adjustment are shown. The *y*-axis is in logarithm 10 scale. R3R: three doses of RTS,S/AS01_E_ and a RTS,S/AS01_E_ booster. R3C: three doses of RTS,S/AS01_E_ and a comparator booster. C3C: three doses of a comparator vaccine and a comparator booster.

**Fig. 11:**
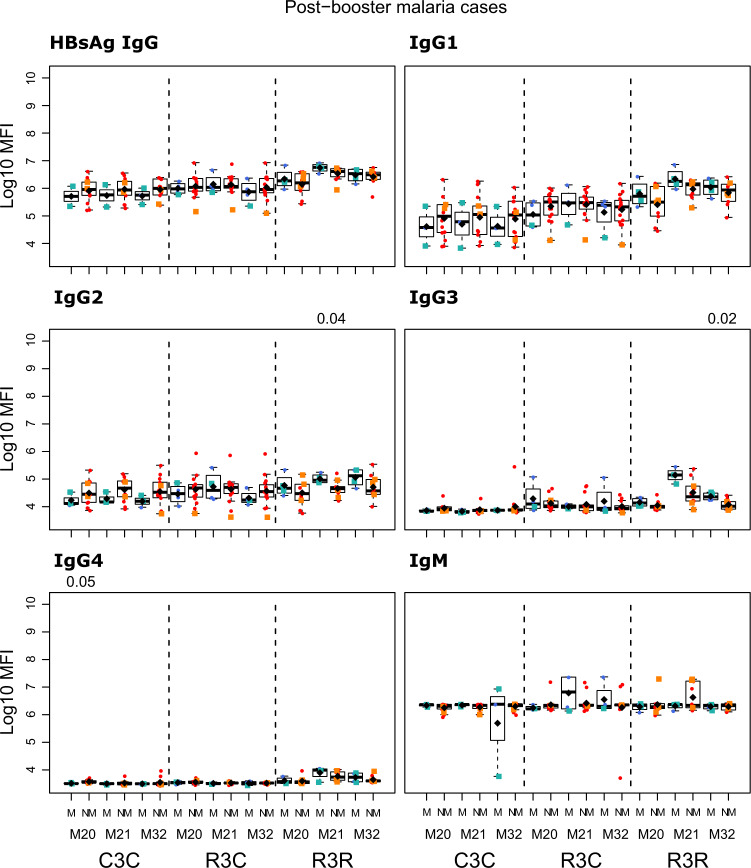
Immunogenicity stratified by clinical malaria after M21: total IgG, IgG1-4 subclasses and IgM for HBsAg at month (M) 20, 21, and 32 for RTS,S/AS01 vaccinees with (R3R) and without (R3C) booster, and comparator (C3C). Stratified analysis by malaria after M21, subjects who presented with clinical malaria (M = blue) and subjects without malaria (NM = red). Subjects who presented with clinical malaria before M20 are represented with green and orange squares. Boxplots with medians, interquartile ranges (IQR), upper whisker as the smallest between maximum × value and Q3 + 1.5*IQR, lower whisker as the largest between minimum × value and Q1 – 1.5*IQR, and log_10_(geometric mean(MFI)) (diamond). Non-parametric tests were used to compare levels with or without clinical malaria (NM vs. M). *p*-values were adjusted for multiple comparisons, but none was significant. Only *p*-values < 0.05 before adjustment are shown. The *y-*axis is in logarithm 10 scale. R3R: three doses of RTS,S/AS01_E_ and a RTS,S/AS01_E_ booster. R3C: three doses of RTS,S/AS01_E_ and a comparator booster. C3C: three doses of a comparator vaccine and a comparator booster.

The study was not designed to assess associations with future malaria risk but we had some provisional findings. IgG2 and IgG3 to vaccine antigens in the R3R group at M21 were higher in those subjects with subsequent clinical malaria, but this difference was not statistically significant. Only for anti-NANP IgG2 and anti-HBsAg IgG2 and IgG3 levels, differences had *p* < 0.05 before adjusting for multiple testing (Figs. 8–11 and Supplementary Table 3). In contrast, IgG1 and IgG4 levels to NANP and CSP FL in the R3R group at M21 were lower in malaria cases, but not significantly. An opposite pattern consisting of higher levels in malaria cases was observed in the R3C group. Fold-change in IgM levels against CSP constructs from M20 to M21 in the R3R group was lower in malaria cases (*p* < 0.05 before adjustment) (Supplementary Figs. 8 and 9). This contrasted to what was observed in the R3C group who had higher fold-change in IgM levels in malaria cases. Additionally, the fold-change in anti-HBsAg IgG3 levels was higher in malaria cases (*p* < 0.05 before adjustment). In most cases, there was no statistically significant difference between subjects presenting with clinical malaria after M21 and those who did not.

### Effect of RTS,S booster vaccination on antibodies to blood stage antigens

**Fig. 12:**
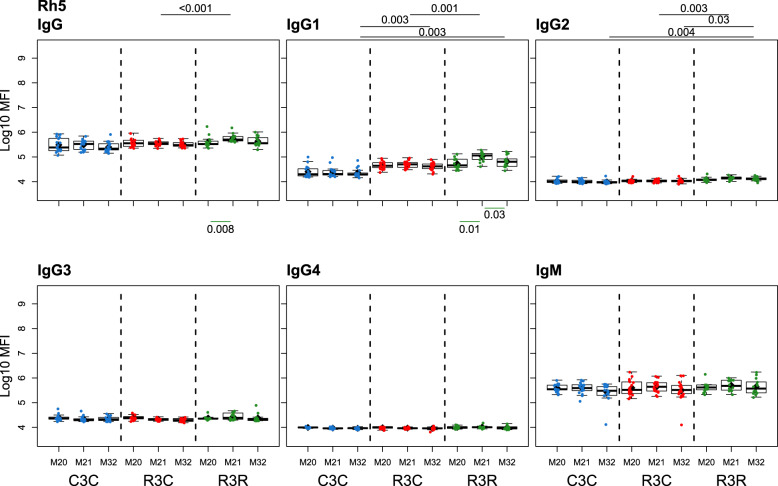
RTS,S/AS01_E_ booster and long-term immunogenicity against the blood stage antigen Rh5: total IgG, IgG1-4 subclasses and IgM at month (M) 20, 21, and 32 for RTS,S/AS01 vaccinees with (R3R) and without (R3C) booster, and comparator (C3C). Boxplots with medians, interquartile ranges (IQR), upper whisker as the smallest between maximum × value and Q3 + 1.5*IQR, lower whisker as the largest between minimum × value and Q1 – 1.5*IQR, and log_10_(geometric mean(MFI)) (diamond). Non-parametric tests were used to compare the booster response (M20 vs. M21) and the long-term immunogenicity (M21 vs. M32), as well as to compare the R3C and R3R groups at each timepoint. Only *p*-values < 0.05 after adjustment for multiple testing are shown. The *y*-axis is in logarithm 10 scale. R3R (green): three doses of RTS,S/AS01_E_ and a RTS,S/AS01_E_ booster. R3C (red): three doses of RTS,S/AS01_E_ and a comparator booster. C3C (blue): three doses of a comparator vaccine and a comparator booster.

**Fig. 13:**
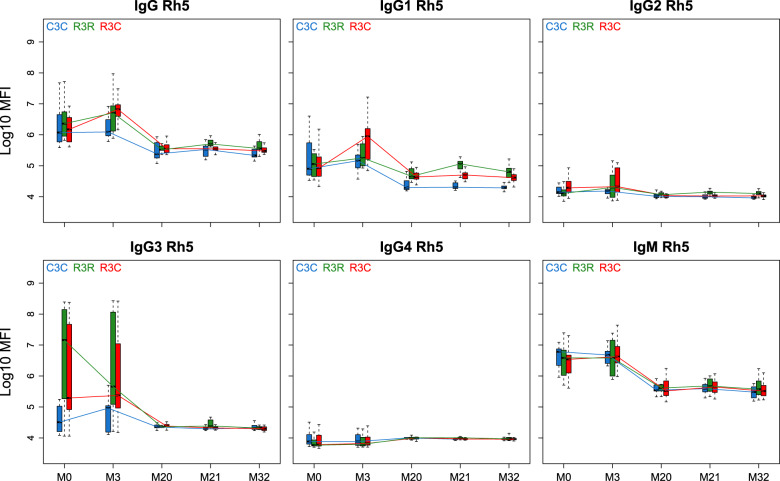
Antibody responses against the blood stage antigen Rh5 for months (M) 0, 3, 20, 21, and 32 for IgG, IgG1, IgG2, IgG3, IgG4 and IgM. Boxplots with median, interquartile ranges (IQR), upper whisker as the smallest between maximum × value and Q3 + 1.5*IQR and lower whisker as the largest between minimum × value and Q1 – 1.5*IQR. The *y*-axis is in logarithm 10 scale. Data from months 0 and 3 were obtained from a previous study in the same individuals [7], thus a batch effect might be present. R3R (green): three doses of RTS,S/AS01_E_ and a RTS,S/AS01_E_ booster at month 20. R3C (red): three doses of RTS,S/AS01_E_ and a comparator booster. C3C (blue): three doses of a comparator vaccine and a comparator booster.

For most of the blood stage antigens we studied, we could not detect differences in antibody levels before and after the booster dose, nor when comparing the R3R, R3C, and C3C groups. There were some differences (*p* < 0.05 before adjustment) in antibody levels at M3 and/or M21 for MSP5, MSP1_42_, MSP1-BL2, Rh4.2, EBA140 and EBA175 (Supplementary Figs. 10–15 and Supplementary Table 4). Interestingly, Rh5 antibodies showed a consistent change in levels after the RTS,S booster for IgG and all IgG subclasses, with higher levels in the R3R group (Figs. 12 and 13; Supplementary Fig. 16 and Supplementary Table 4). In the case of IgG, IgG1 and IgG2 the differences were significant both in the short (M21) and long (M32) term, while for IgG3 and IgG4 differences were only at M21 with *p* < 0.05 before adjustment. Curiously, overall levels diminished over follow-up with the exception of IgG4.

Age did not have a significant effect on the antibody levels against the studied blood stage antigens (Supplementary Figs. 16–23 and Supplementary Table 5). Individuals who were classified as having had a case of clinical malaria before M20 tended to have higher levels of antibody to blood stage antigens at M20-32 but this difference was not statistically significant (Supplementary Figs. 24–30 and Supplementary Table 6). The most remarkable difference was the levels of MSP1_42_ that acted as a marker for malaria exposure, showing higher levels in those who had clinical malaria, in particular for IgG, IgG1 and IgG2. Overall, the responses showed a general pattern of higher levels at all time points for all vaccination groups in individuals who subsequently presented with a malaria case but it was not significant (Supplementary Figs. 31–37 and Supplementary Table 6).

## Discussion

This study confirms that the RTS,S/AS01_E_ booster dose increases total IgG levels against vaccine antigens and elucidates its differing effect on IgG subclasses and IgM not previously studied. We describe for the first time the long-term RTS,S/AS01_E_ antibody response to different antigens and CSP epitopes. The booster dose increased total IgG, IgG1, IgG3, and IgG4 for all vaccine antigens compared to pre-booster levels, and they remained above the levels of non-vaccinated individuals during the entire follow-up period. Remarkably, the fourth dose did not induce an increase in IgG2 levels (although the primary vaccination did) but it increased IgG1 and IgG3 levels, which may explain how the booster led to higher efficacy overall. IgG1 and IgG3 can effectively fix complement and promote interactions with Fcγ-receptors on phagocytes^10^, which could be contributing to RTS,S-induced protection. IgG2 and IgG4, on the contrary, are non-cytophilic antibodies unable to fix complement and to interact with Fcγ-receptors^10^.

The profile of antibody responses seems to be epitope-specific. Previously, the bulk of studies had only evaluated NANP antibodies and have provided clear evidence that NANP antibodies are associated with protection^6^, being the established immunodominant region of the vaccine antigen^4^. However, there is evidence that antibodies against C-term are involved in phagocytic activity in US naive adults^13^, and RTS,S vaccine-induced antibodies to the C-term among children can promote complement fixation^14^. Also, in our previous work we have found that post-primary vaccination, the avidity of the IgG response to CSP C-term was associated with protection^12^. Here, we show that the booster dose increases levels of antibodies against both NANP and the C-term, and that the responses against these two regions may behave differently. Antibody levels to NANP were higher compared to C-term, but the proportional increase 1 month after the booster dose was not different.

Previously it was reported that the IgG levels to NANP were increased by the booster dose, but the peak post-booster levels were lower than following primary vaccination^6^. In this study, we found similar results for IgG subclasses—there was boosting but levels overall were lower than following primary vaccination^7^. Remarkably, IgG4 levels against CSP kept increasing with time. The RTS,S pattern differs to other vaccines in which the peak for the booster response is higher than the peak for the primary vaccination^18^. The unusual response to the booster dose could be caused by different factors. These factors include the response to primary vaccination^19^, but also the booster dose or the primary vaccination schedule^20,21^. For instance, it has been reported that for some vaccines high residual levels of vaccine antibodies have a negative effect on the post-booster response^19^; however, our study did not find evidence for a negative correlation between M20 and M21 antibody levels (Supplementary Figs. 38 and 39). There is also evidence from the response to a Meningococcal conjugated vaccine that primary vaccination administered with a short interval doses might lead to higher antibodies at the primary vaccination peak, but a higher number of doses lead to a lower post-booster response^20^. The effect of the dosing interval on the responses to the RTS,S vaccine was observed on a phase 2 trial that compared a 0, 1, 2 months vs. 0, 1, 7 months schedule and showed that the highest peak was observed following the 0, 1, 2 month schedule^21^. Also, the booster dose might induce different IgG subclass patterns to primary vaccination because it is acting on immune memory cells such as B memory cells and it might be inducing class switch and increasing antibody affinity^19,22^. The AS01_E_ adjuvant and the innate response and cytokine milieu that it elicits likely affect the evolution of antibody subclass patterns observed. In addition, it may be influenced by differences in immune development due to older age and environmental exposures^19^. For instance, it has been reported that malaria transmission affects the antibody subclasses patterns in subjects who had malaria episodes^23^. Nevertheless, it is important to note that because primary and booster antibody data were not analyzed at the same time, the comparison of antibodies between time points in our study should be interpreted carefully.

We are reporting for the first time the antibody booster response against HBsAg. Although the booster dose did increase IgG, IgG1, IgG3 and IgG4 antibodies, the increments were lower than those of anti-CSP antibodies, and were similar to the response induced by primary vaccination in the Manhiça site^7^. Interestingly, the results of a trial that analyzed the response to a hepatitis B vaccine booster after RTS,S primary vaccination showed an increased response in HBsAg IgG concentrations compared to primary vaccination^24^. The responses to HBsAg may be an indication of the quality of the immune response or the immune status of the child and the capacity to respond to vaccination and it requires further investigation.

It has been previously reported that antibodies after primary vaccination are lower in the infant group than in the children group. This may be due to the interference of maternal antibodies against CSP or intrinsic differences in the developing and functional competence of the immune system^5^. Antibodies seem to be lower in infants than children also at months 20, 21, and 32 but only for total IgG and IgG1 against CSP epitopes, not for HBsAg responses, even though maternal antibodies are no longer present at this age. At the time of the booster, both age groups have passed the critical age of 2 years for immune ontogeny and their immune system has achieved a more adult status^25,26^, therefore, major differences driven by intrinsic changes in the immune system are less likely at this timepoint. Instead, differences detected may reflect a distinct establishment of a memory response in infants and children after primary vaccination that differentially affects the boosting of responses.

One of the factors identified thus far in this and our previous studies^7^, with an impact on vaccine responses, is prior malaria exposure. Preceding malaria cases were associated with lower IgG, IgG1, and IgG3 levels to CSP constructs at the time points studied. How malaria episodes may affect immunization outcomes is another key issue, since it may affect any malaria vaccine and needs to be addressed in follow-up studies.

The study was not powered to assess association of antibodies with malaria risk but we obtained some preliminary findings. Interestingly, in children with malaria episodes post-booster dose, the IgG2 and IgG3 levels at M21 were higher than non-malaria controls, while IgG, IgG1, and IgG4 levels were lower, although with the available sample size this was not statistically significant for most comparisons. These results are in line with the findings of our previous study^7^ in which IgG2 levels to RTS,S antigens were higher in malaria cases than in controls, whereas IgG1 levels were lower. The booster dose might be increasing protection not only by increasing total IgG levels, but also by not inducing IgG2 that could be detrimental, or not protective according to our previous results. However, the tendency for malaria cases to be associated with higher levels of IgG3 and lower levels for IgG4 to CSP constructs was opposite to what was reported to occur after primary vaccination^7^ where a higher ratio between cytophilic (IgG1 and IgG3) to non-cytophilic (IgG2 and IgG4) CSP antibodies was associated with protection. The association between IgG4 levels and protection is consistent with findings of Chaudhury et al.^27^ who reported that only IgG4 levels were positively associated with increased vaccine efficacy in malaria-naive adults under the fractional dose regime. We have also previously found an association between IgG4 responses to non-RTS,S antigens after primary vaccination and protection^8^. IgG4 antibodies are associated with repeated or long-term exposure to antigens and have been linked to induction of tolerance, for instance higher IgG4/IgE ratios are associated with better food tolerance, as IgG4 competes with IgE^28^. In the context of helminth infections, high IgG4 is associated with asymptomatic infection for some parasites^10^. However, there is conflicting evidence on the role of IgG2 and IgG4 on protection against malaria, whilst more information exists on the protective role of IgG1 and IgG3. In the context of naturally acquired immunity, the ratio of cytophilic (IgG1 + IgG3) to non-cytophilic subclasses (IgG2 + IgG4) is generally higher in subjects with uncomplicated malaria compared to subjects with complicated malaria, and higher in subjects protected from malaria^7,29^. Additionally, it has been reported that the IgG2/IgG4 ratio is higher in subjects with uncomplicated malaria^30^. However, it has also been observed that IgG2 and IgG4 with high avidity are found in subjects with uncomplicated malaria compared to complicated malaria^29^. In contrast, the Chaudhury et al.^27^ study assessing avidity and opsonization reported that RTS,S protection was mediated by IgG4 against the C-term of CSP. All of this evidence indicates that not only antibody levels are important for protection but also the balance between subclasses.

We note that the pattern of higher IgG, IgG1, and IgG4 levels to CSP FL and C-term in non-malaria cases was not apparent for NANP.

We previously observed that after primary vaccination, HBsAg antibody responses were associated with malaria protection^7,12^. On the contrary, 1 month after receiving the RTS,S booster dose, anti-HBsAg IgG2 and IgG3 levels appeared as risk factors for future malaria episodes, further indicating that the nature and role of responses may differ following a primary and a booster immunization.

The analysis of the phase 3 clinical trial found that children who did not receive the booster dose were at higher risk of severe malaria than the comparator (non-RTS,S) group^3^. It was hypothesized that the primary vaccination had prevented vaccinees from acquiring natural immunity, as has been predicted for other malaria prevention tools^16^, increasing the risk of severe malaria in those individuals in whom infection reached the erythrocytic stage. However, on a longer follow-up study of up to 7 years on 3 of 11 sites, no increased risk was found for severe malaria between those groups that received the RTS,S/AS01 vaccine and the control group^31^. Antibody responses to asexual blood stage antigens have been studied previously with samples from phase 2 clinical trials and showed a reduced antibody response in RTS,S vaccinees, but these trials did not include a booster dose^17,32,33^. We detected a decreased antibody response to certain vaccine-unrelated *P. falciparum* antigens after primary vaccination in the phase 3 trial as well^8,9^. However, we also observed an induction of antibody responses to other *P. falciparum* antigens following RTS,S vaccination (MSP1-BL2, EBA140, EBA175, and Rh4.2), which could contribute to malaria protection^8,9^. Here, we did not find consistent differences in the NAI response between booster groups except for Rh5. Interestingly and in line with our previous results, Rh5 IgG and IgG1-2 levels were higher in the RTS,S booster group than in the comparator vaccine groups at M21 and M32, and R3C levels were either higher or did not differ from C3C. This finding requires future investigation to understand the basis and clinical relevance of this effect, especially since Rh5 is a leading vaccine candidate^34^, and because Rh5 antibody concentrations need to be very high to actually confer protection^35^. However, these observations are important as they may explain why an anti-sporozoite infection vaccine also protects against clinical disease in the parasite blood stage, considering that Rh5 plays an essential role during erythrocyte invasion by *P. falciparum* merozoites^36–38^. Additionally, IgG to MSP5 showed higher levels after RTS,S booster dose compared to the comparator booster group, but this was not statistically significant.

Our findings are limited because of a small sample size and because data were obtained only for Manhiça. Therefore, a larger longitudinal study with samples from different sites is necessary to corroborate these data. This is particularly important in our case because there are some special considerations about Manhiça that limits the generalization of these findings: (1) at the time of the study malaria transmission was low^3,39^, (2) there were unexpected results of VE in the phase 3 clinical trial, i.e., VE was lower in the R3R than in the R3C group, contrary to most sites, and (3) Manhiça has a high HIV prevalence^40^. HIV infection was associated with a reduced immunogenicity to the vaccine in a phase 3 trial exploratory analysis but it was concluded that HIV-infected children should not be excluded from RTS,S vaccination^41^.

Despite the constraints, our study provides new and interesting clues to the immune response elicited by the RTS,S booster dose. Additionally, avidity and functional antibody responses should be assessed, and these results integrated with cellular data to address memory responses induced by the booster. This information is necessary for a deeper understanding of the mechanisms of action of the vaccine, as well as the determination of the factors causing partial and short VE. Results of these studies are required for the rational design and deployment of improved CSP-based vaccines and other malaria vaccines with an increased and long-term efficacy.

## Methods

### Population and study design

This study was performed using plasma samples previously collected from subjects in Manhiça, Mozambique, a site of low malaria transmission intensity^3,39^, as part of the MAL067 study ancillary to the phase 3 randomized clinical trial MAL055 (NCT00866619)^3^. A subset of 50 individuals (24 children 5–17 months and 26 infants 6–12 weeks) was selected from those previously analyzed^7–9^ who had available antibody data from M0 (baseline) and M3 (one month after third dose) and plasma samples for M20 (booster dose), M21, and M32 (Supplementary Table 7). The subjects had either received three doses of the RTS,S/AS01_E_ vaccine and a RTS,S/AS01_E_ booster (R3R, *n* = 14) at M20, three doses of RTS,S/AS01_E_ and a comparator booster (R3C, *n* = 19), or three doses and a booster of a comparator vaccine (C3C, *n* = 17) (Supplementary Fig. 40). The comparator vaccines used in the primary series were a Meningococcal C Conjugate Vaccine (Menjugate™) in the 6–12 weeks age category, and a cell-culture rabies vaccine (VeroRab™) in the 5–17 months age category. The booster comparator was Menjugate™ for both age groups. Clinical malaria cases were detected by passive case detection and defined as fever of at least 37.5 °C and any asexual *P. falciparum* parasitemia by microscopy^3^. The prevalence of HIV infection in the Manhiça area was around 40% in adults^40^. HIV infection was not a protocol exclusion/inclusion criteria, but only healthy children were included in the study. HIV testing was not a trial procedure. The study protocol was approved by the Ethics Committees of PATH-MVI (REC) in the US, Hospital Clínic in Spain (CEIm) and the CNBS in Mozambique, and written informed consent was obtained from parents or guardians before recruitment.

### Antibody luminex assays

Antibody response was analyzed using a quantitative suspension array technology (qSAT). MAGPlex beads were coupled separately to: three CSP constructs (FL, C-term, NANP-repeat region) and HBsAg that are antigenic components of the RTS,S vaccine; seven *P. falciparum* blood stage antigens (MSP1 [block 2 and MSP1_42_ fragments, 3D7 strain], MSP5, EBA140, EBA175 region 3-5, Rh4.2 and Rh5) that were shown to be affected by vaccination in our previous studies^7–9^ and/or that are leading vaccine candidates; and Glutathione S-transferase (GST) as a control for antigens co-expressed with a GST tag (Supplementary Table 8)^42^. The coupling of the beads to the antigens was performed as described previously^43^.

Antigen-coupled beads were added to a 96-well μClear® flat bottom plate (Greiner Bio-One) in multiplex (1000 microspheres/analyte/well) resuspended in 50 μL of PBS, 1% BSA, 0.05% Azide pH 7.4 (PBS-BN). Fifty microliters of sample, negative or positive control were added to wells and incubated overnight at 4 ºC in a shaker protected from light. Plates were washed three times with 200 μL/well of wash buffer (PBS-Tween 20: 0.05%) using a manual magnetic washer. Then, 100 μL of biotinylated secondary antibody were added diluted in PBS-BN: anti-human IgG 1/2500 (B1140 Sigma), anti-human IgG1 1/4000 (ab99775 Abcam), anti-human IgG3 1/1000 (B3523 Sigma), and anti-human IgM 1/1000 (B1265 Sigma). For IgG2 and IG4, mouse anti-human IgG2 1/500 and IgG4 1/500 (MA1-34755 and MA5-16716 Thermo Fisher), respectively, were added, followed by biotinylated goat anti-mouse IgG 1/40,000 for IgG2 and 1/10,000 for IgG4 (B7401 Sigma) in PBS-BN. All antibody incubations were performed for 45 min, at room temperature, in agitation and protected from light. Again, plates were washed as before and 100 μL/well streptavidin-R-phycoerythrin 1/1000 (42250 Sigma) in PBS-BN was added to all wells and incubated 30 min, at room temperature, in agitation and protected from light. Plates were washed as before and resuspended in 100 μL/well of PBS-BN. Plates were stored at 4 °C overnight protected from light and read the next day using the Luminex xMAP® 100/200 analyzer; at least 50 microspheres per analyte were acquired per sample and Report Gain was set as High PMT.

For IgG, IgG1, IgG3, and IgM, 20 serial dilutions 1:2 of a positive control were used to perform antigen-subclass-specific standard curves. For IgG2, 16 serial dilutions 1:2 were used. For IgG4, no standard curve was performed and only one positive control dilution was included. The positive control consisted of a WHO Reference Reagent for anti-malaria *P. falciparum* human serum (NIBSC code: 10/198)^42,44^ at 1:50 mixed with a pool of plasmas from RTS,S/AS02 vaccinated children^42,45^ with high IgG levels against CSP at 1:200. Blanks were added to each plate in duplicates and two negative controls samples from malaria-naive adults were added in each plate. Test samples were assayed at three dilutions for IgG (500, 20,000, 500,000), IgG1, IgG3 (100, 2500, 100,000) and IgM (100, 1000, 25,000) to ensure that at least one dilution lie in the linear range of the respective standard curve, i.e., close to the highest slope between two dilution points. Owing to the low levels previously observed in these samples for IgG2 and IgG4 in Ubillos et al.^7^ only one dilution (1/50) was used. Sample distribution across plates was designed to ensure a balanced distribution of vaccination groups, sex, age cohorts, and malaria cases. The three time points for each individual and the respective dilutions were placed on the same plate. Data were captured using xPonent software, and antibody levels were measured as MFI.

#### Data pre-processing

The standard curve for each antigen-isotype/subclass-plate was estimated using the drLumi R package flow^46^, fitted in a 4- or 5-parameter logistic (4-PL or 5-PL) regression model, and data points logarithmically transformed. To select the sample dilution for IgG, IgG1, and IgG3 in the linear part of the sigmoidal curve (antigen, isotype/subclass and plate specific), an algorithm that detects the two points with the highest slope between them was used. The slope was computed as: *m* = (MFI_i_ – MFI_i+1_)/(dilution_factor_i _– dilution_factor_i+1_). The mean MFI value of the two points was computed and used as the reference value, but the standard curves were visually inspected and if the model did not converge, the *R*^2^ < 0.9 or the curve maximum values were < 15,000 MFI, a 15,000 MFI reference value was set instead of the highest slope criteria. The nearest MFI of the test sample to the reference value was determined and the corresponding dilution was selected. Since only one dilution was used for IgG2 and IgG4, the standard curves were not used to select a dilution. The MFI measurement of the selected dilution was corrected multiplying by its corresponding dilution factor and transformed to log_10_ scale to stabilize the variance. Blank and GST signals were not subtracted. Blanks were used to measure background signal, and GST to assess for unspecific binding to the GST-fused antigens (CSP FL, CSP C-term, and CSP-NANP). Background values were below 500 MFI, and no correlation was found between IgG to GST and IgG to GST-fused antigens (Supplementary Figs. 41 and 42).

### Statistical analysis

Descriptive comparisons of Ig isotype/subclass levels to specific antigens (log_10_ transformed MFI) at each visit were done by boxplots with log_10_(geometric mean), medians, interquartile ranges (IQR), upper whisker as the smallest between maximum × value and Q3 + 1.5*IQR and lower whisker as the largest between minimum × value and Q1 – 1.5*IQR. Wilcoxon-Signed Rank Test between M20 and M21 for the R3R vaccination group, and between M21 and M32 for the R3R and R3C vaccination groups were performed to determine if the antibody levels changed significantly 1 month and 1 year after the booster. Additionally, Mann–Whitney tests were performed to compare the R3R and R3C groups at each timepoint. *p-*values were adjusted for multiple testing using p.adjust on R^47^ by the Holm approach for IgG and for IgM to control for family wise error, and by the Benjamini–Hochberg approach for IgG1-4, altogether to control for the false-discovery rate since there were more tests. The comparisons between time points were corrected separately from the comparisons between vaccination groups, likewise the comparisons for vaccine antigens were corrected separately from blood stage antigens. Data for M0 and M3 from our previous study^7^ was used to analyze the kinetics of the antibody response throughout the 5 time points in the clinical trial. Adjusted *p*-values were considered significant when <0.05. The qSAT assay of M20, M21, and M32 samples was performed in the same laboratory using the same reagents and under similar conditions as the assay of the M0 and M3 samples, but they were not executed at the same time and a smaller set of antigens was used.

Stratified analyses and Mann–Whitney tests for independent groups and Wilcoxon-Signed Rank Tests for paired samples were performed between age groups and between malaria cases and controls, for each timepoint. *p-*values were adjusted following the same strategy as above. There were no reported malaria cases between M20 and M21. The change in antibody levels between M20 and M21 was calculated as log_10_MFI(M21) – log_10_MFI(M20) and compared between individuals who either did or did not present with clinical malaria after M21. All data analysis and plots were performed using R packages gridExtra^48^, dplyr^49^, ggplot2^50^, tidyr^51^, and psych^52^.

### Reporting summary

Further information on research design is available in the Nature Research Reporting Summary linked to this article.

## Supplementary information

Supplementary Information

reporting summary

## Data Availability

The datasets generated in the current study are fully available at the Dipòsit Digital de la Universitat de Barcelona at http://hdl.handle.net/2445/164777.
