# Author Correction: Antibody responses to the RTS,S/AS01_E_ vaccine and *Plasmodium falciparum* antigens after a booster dose within the phase 3 trial in Mozambique

**DOI:** 10.1038/s41541-022-00515-8

**Published:** 2022-08-08

**Authors:** Lina Sánchez, Marta Vidal, Chenjerai Jairoce, Ruth Aguilar, Itziar Ubillos, Inocencia Cuamba, Augusto J. Nhabomba, Nana Aba Williams, Núria Díez-Padrisa, David Cavanagh, Evelina Angov, Ross L. Coppel, Deepak Gaur, James G. Beeson, Sheetij Dutta, Pedro Aide, Joseph J. Campo, Gemma Moncunill, Carlota Dobaño

**Affiliations:** 1 ISGlobal, Hospital Clínic—Universitat de Barcelona, Barcelona, Catalonia Spain; 2 UnivLyon, Université Claude Bernard Lyon 1, 69100 Villeurbanne, France; 3 Centro de Investigação em Saúde de Manhiça (CISM), Maputo, Mozambique; 4 Institute of Immunology & Infection Research and Centre for Immunity, Infection & Evolution, Ashworth Laboratories, School of Biological Sciences, University of Edinburgh, King’s Buildings, Edinburgh, UK; 5 U.S. Military Malaria Vaccine Program, Walter Reed Army Institute of Research (WRAIR), Silver Spring, MD USA; 6 Infection and Immunity Program, Monash Biomedicine Discovery Institute and Department of Microbiology, Monash University, Melbourne, VIC Australia; 7 Malaria Group, International Centre for Genetic Engineering and Biotechnology (ICGEB), New Delhi, India; 8 Laboratory of Malaria and Vaccine Research, School of Biotechnology, Jawaharlal Nehru University, New Delhi, India; 9 Burnet Institute, Melbourne, VIC Australia

**Keywords:** Malaria, Adaptive immunity, Vaccines

Correction to: *npj Vaccines* 10.1038/s41541-020-0192-7, published online 04 June 2020

In the original version of this Article, the Data Availability statement was missing the link to the datasets generated during the study. The Data Availability statement has now been updated in both the HTML and PDF versions of the Article.

