# Supplementary material for: Antibody responses to the RTS,S/AS01E vaccine and Plasmodium falciparum antigens after a booster dose within the phase 3 trial in Mozambique
Source: NPJ Vaccines. 2020 Jun 4;5:46. doi: 10.1038/s41541-020-0192-7 (PMC7272643; doi:10.1038/s41541-020-0192-7)
Supplement: Supplementary file 1 — Supplementary Information [file 41541_2020_192_MOESM1_ESM.pdf]

Supplementary Figures and Tables

Supplementary Table 1. RTS,S/AS01E booster and long-term immunogenicity for total IgG, IgG1-4 subclasses and IgM against vaccine antigens: Non-parametric test p-values for comparison of the booster response (M20 vs M21) and the long-term immunogenicity (M21 vs M32), as well as to compare the R3C and R3R groups at each timepoint. for RTS,S/AS01 vaccinees with (R3R) and without (R3C) booster, and comparator (C3C). Adjusted p values are shown in parenthesis. P-values <0.05 before adjustment are highlighted in yellow and P-values <0.001 before adjustment in green. R3R: three doses of RTS,S/AS01E and a RTS,S/AS01E booster. R3C: three doses of RTS,S/AS01E and a comparator booster. C3C: three doses of a comparator vaccine and a comparator booster.

		Comparison between vaccination groups					Comparison Time points		
		R3R vs R3C			R3R vs C3C	R3C vs C3C	M20vsM21	M21vsM32	
**Isotype**	**Antigen**	**M20**	**M21**	**M32**	**M32**	**M32**	**R3R**	**R3R**	**R3C**
IgG	CSP_FL	1	<0.001	<0.001	<0.001	<0.001	0.001	0.02	0.001
IgG	Cterm	1	<0.001	<0.001	<0.001	<0.001	0.001	0.007	0.001
IgG	HBsAg	1	0.002	0.01	<0.001	1	0.007	0.03	0.007
IgG	NANP	1	<0.001	0.19	<0.001	<0.001	0.001	0.001	<0.001
IgG1	CSP_FL	0.63	<0.001	0.001	<0.001	<0.001	<0.001	0.03	<0.001
IgG1	Cterm	0.59	<0.001	<0.001	<0.001	<0.001	<0.001	0.002	0.001
IgG1	HBsAg	0.38	<0.001	<0.001	<0.001	0.14	0.002	0.08	0.005
IgG1	NANP	1	0.004	0.1	<0.001	<0.001	0.002	0.002	<0.001
IgG2	CSP_FL	0.17	0.21	0.32	<0.001	<0.001	0.84	0.27	0.048
IgG2	Cterm	0.044	0.05	0.03	<0.001	<0.001	0.88	0.63	0.26
IgG2	HBsAg	1	0.41	0.09	0.07	0.96	0.08	1	0.29
IgG2	NANP	0.16	0.09	0.79	<0.001	<0.001	0.75	0.87	0.08
IgG3	CSP_FL	0.81	0.13	0.1	<0.001	<0.001	0.007	<0.001	0.96
IgG3	Cterm	0.95	0.001	0.08	<0.001	<0.001	0.002	0.001	0.26
IgG3	HBsAg	1	<0.001	0.1	0.001	0.049	0.001	<0.001	0.30
IgG3	NANP	0.41	0.08	0.15	0.08	0.002	0.01	<0.001	0.41
IgG4	CSP_FL	0.23	0.002	0.08	<0.001	<0.001	<0.001	1	0.01
IgG4	Cterm	0.16	<0.001	0.02	<0.001	<0.001	<0.001	0.75	0.057
IgG4	HBsAg	0.54	<0.001	<0.001	<0.001	0.17	0.006	0.009	0.88
IgG4	NANP	0.21	<0.001	0.047	<0.001	<0.001	0.08	0.001	0.95
IgM	CSP_FL	1	1	1	1	1	1	1	0.96
IgM	Cterm	1	1	1	0.09	1	1	1	1
IgM	HBsAg	1	1	1	1	1	1	1	1
IgM	NANP	1	1	1	1	1	1	1	1

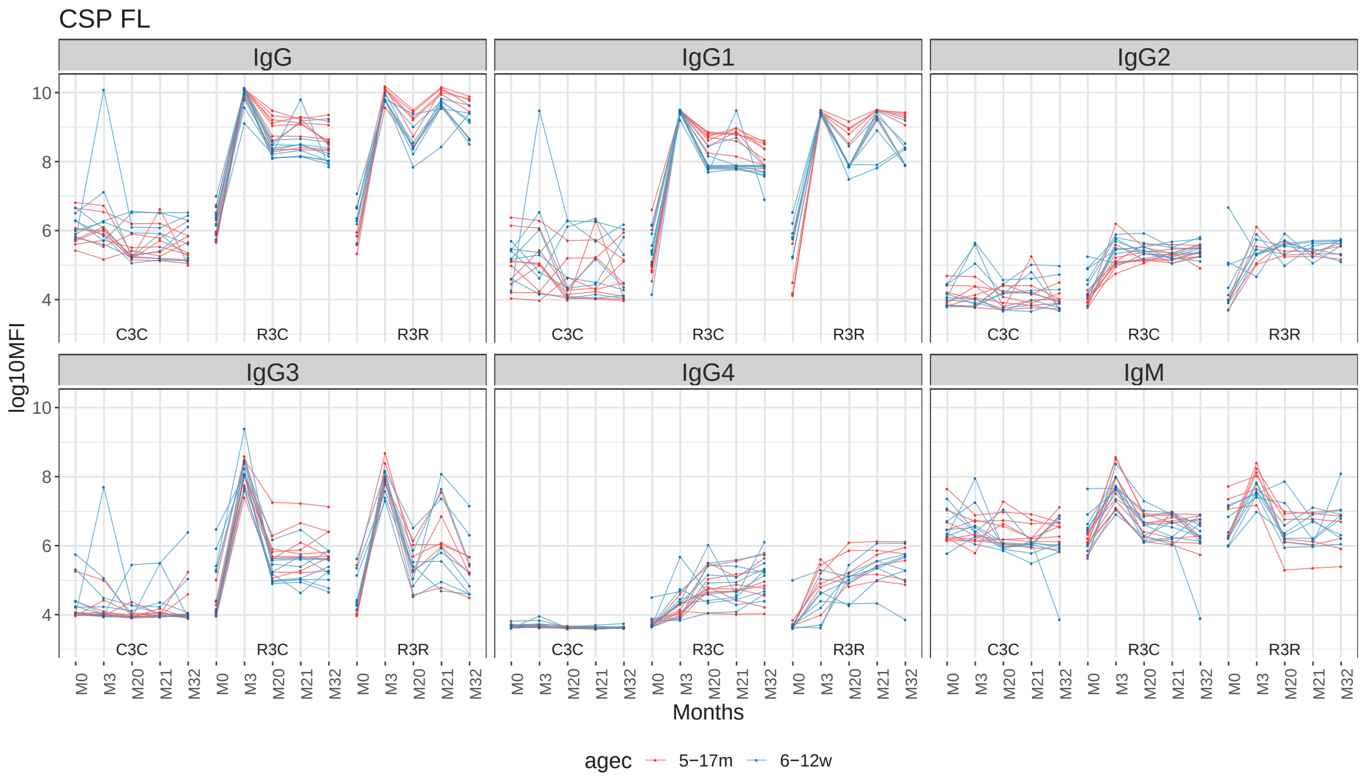

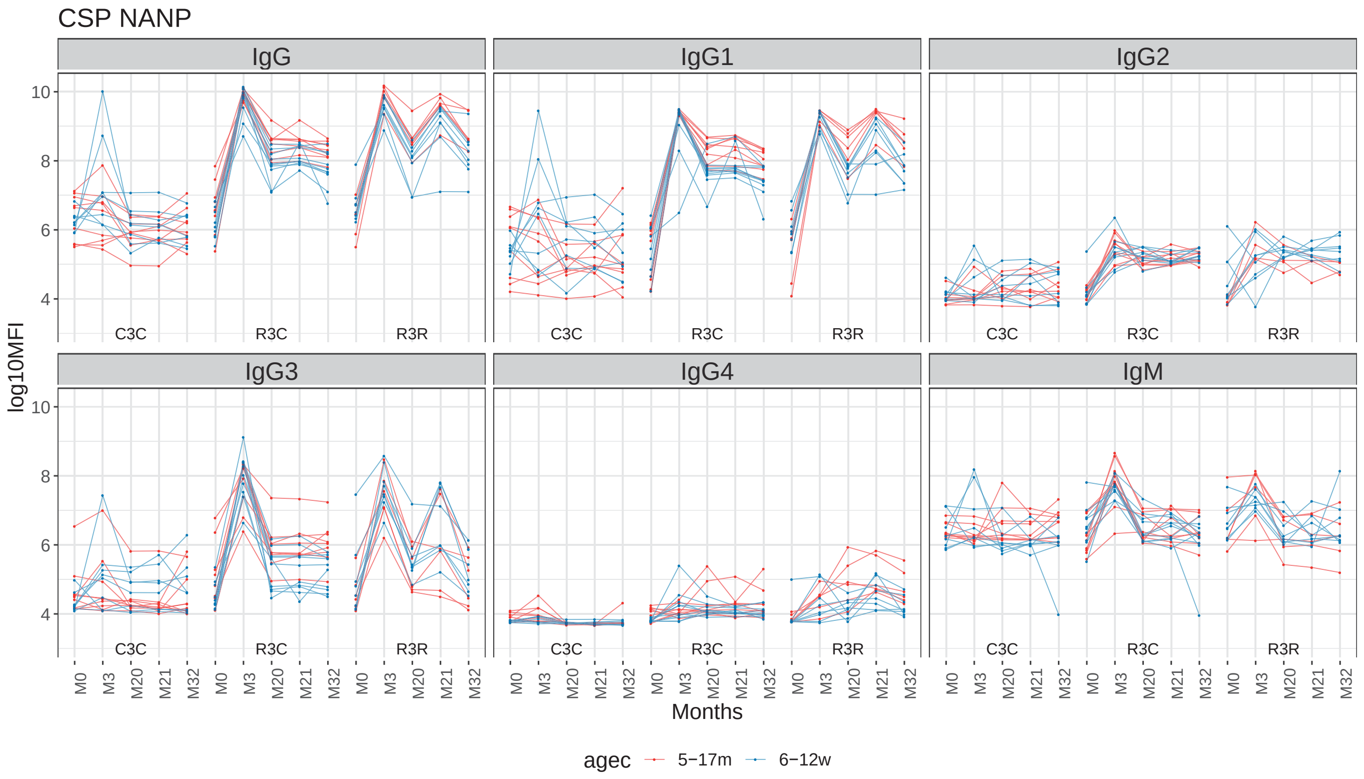

Supplementary Figure 1. Antibody responses against vaccine antigens CSP FL and CSP NANP for months (M) 0, 3, 20, 21 and 32 for IgG, IgG1, IgG2, IgG3, IgG4 and IgM. Line time plots connecting levels for each individual. The y axis is in logarithm 10 scale. Data from months 0 and 3 were obtained from a previous study in the same individuals [5], thus a batch effect might be present. R3R: three doses of RTS,S/AS01E and a RTS,S/AS01E booster at month 20. R3C: three doses of RTS,S/AS01E and a comparator booster. C3C: three doses of a comparator vaccine and a comparator booster. Children age 5-17 months are shown in orange and infants age 6-12 weeks at first immunization are shown in blue.

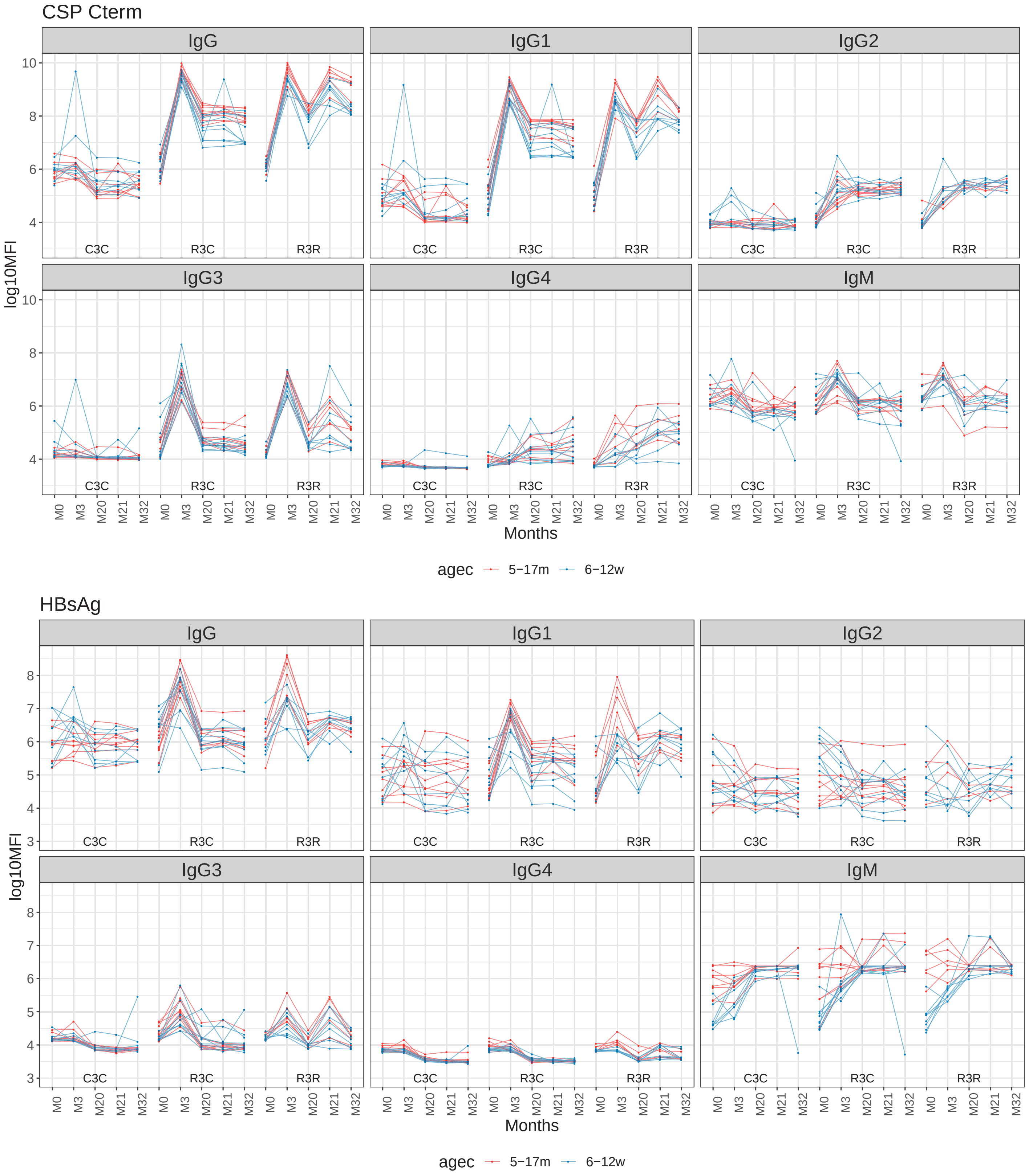

Supplementary Figure 2. Antibody responses against vaccine antigens CSP Cterm and HBsAg for months (M) 0, 3, 20, 21 and 32 for IgG, IgG1, IgG2, IgG3, IgG4 and IgM. Line time plots connecting levels for each individual. The y axis is in logarithm 10 scale. Data from months 0 and 3 were obtained from a previous study in the same individuals [5], thus a batch effect might be present. R3R: three doses of RTS,S/AS01E and a RTS,S/AS01E booster at month 20. R3C: three doses of RTS,S/AS01E and a comparator booster. C3C: three doses of a comparator vaccine and a comparator booster. Children age 5-17 months are shown in orange and infants age 6-12 weeks at first immunization are shown in blue.

Supplementary Table 2. RTS,S/AS01E booster and long-term immunogenicity for total IgG, IgG1-4 subclasses and IgM against vaccine antigens stratified by age: Non-parametric test p-values for comparison of the booster response (M20 vs M21), the long-term immunogenicity (M21 vs M32), and the effect of age (children vs infants), adjusted p values are shown in parenthesis. RTS,S/AS01 vaccinees with (R3R) and without (R3C) booster, and comparator (C3C). Children were age 5-17 months and infants 6-12 weeks at the time of first vaccination. P-values <0.05 before adjustment are highlighted in yellow.

		Comparison between timepoints						Age comparison					
		M20vsM21		M21vsM32				Children vs Infants					
		R3R		R3R		R3C		R3C			R3R		
Isotype	Antigen	Infants	Children	Infants	Children	Infants	Children	M20	M21	M32	M20	M21	M32
IgG	CSP_FL	0.17	0.44	0.44	0.44	0.09	0.44	0.07	0.63	0.20	0.39	0.17	0.39
IgG	Cterm	0.25	0.44	0.44	0.44	0.18	0.20	0.05	0.63	0.39	0.39	0.36	0.36
IgG	HBsAg	0.35	0.44	0.44	0.44	0.44	0.44	1	1	1	1	1	1
IgG	NANP	0.17	0.44	0.17	0.44	0.09	0.17	0.07	0.36	0.33	0.39	0.36	0.39
IgG1	CSP_FL	0.06	0.08	0.15	0.08	0.07	0.06	0.05	0.13	0.046	0.09	0.12	0.13
IgG1	Cterm	0.06	0.08	0.09	0.08	0.09	0.06	0.07	0.16	0.1	0.24	0.09	0.20
IgG1	HBsAg	0.08	0.08	0.45	0.08	0.08	0.18	0.16	0.38	0.29	0.68	0.84	0.68
IgG1	NANP	0.09	0.08	0.09	0.08	0.06	0.06	0.07	0.13	0.046	0.12	0.09	0.11
IgG2	CSP_FL	0.86	0.93	0.54	0.59	0.16	0.28	0.15	0.74	0.73	0.45	0.64	0.45
IgG2	Cterm	0.93	0.45	0.99	0.18	0.65	0.40	0.55	0.99	0.74	0.76	0.64	0.98
IgG2	HBsAg	0.26	0.45	0.86	0.59	0.71	0.34	0.73	0.74	0.85	0.76	0.98	0.68
IgG2	NANP	0.99	0.71	0.79	0.45	0.09	0.71	0.21	1	0.46	0.64	0.35	0.24
IgG3	CSP_FL	0.11	0.12	0.06	0.08	0.93	1	0.12	0.12	0.12	0.76	0.84	0.98
IgG3	Cterm	0.11	0.08	0.08	0.08	0.38	0.79	0.24	0.29	0.21	0.40	0.73	0.91
IgG3	HBsAg	0.08	0.08	0.06	0.08	0.65	0.45	0.45	0.59	0.68	0.45	0.51	0.76
IgG3	NANP	0.08	0.59	0.06	0.08	0.27	1	0.15	0.11	0.046	0.73	0.40	0.59
IgG4	CSP_FL	0.06	0.08	0.99	1	0.07	0.51	0.91	0.85	0.55	0.45	0.68	0.73
IgG4	Cterm	0.06	0.08	0.86	0.82	0.09	0.45	0.68	0.29	1	0.20	0.64	0.51
IgG4	HBsAg	0.06	0.27	0.26	0.08	0.93	0.99	0.35	0.75	0.75	0.29	0.68	0.68
IgG4	NANP	0.11	0.71	0.08	0.08	1	0.99	0.59	0.46	0.45	0.12	0.40	0.16
IgM	CSP_FL	1	1	1	1	1	1	1	1	1	1	1	1
IgM	Cterm	1	0.75	1	1	1	1	1	1	1	1	1	1
IgM	HBsAg	1	1	1	1	1	1	1	1	1	1	1	1
IgM	NANP	1	1	1	1	1	1	1	1	1	1	1	1

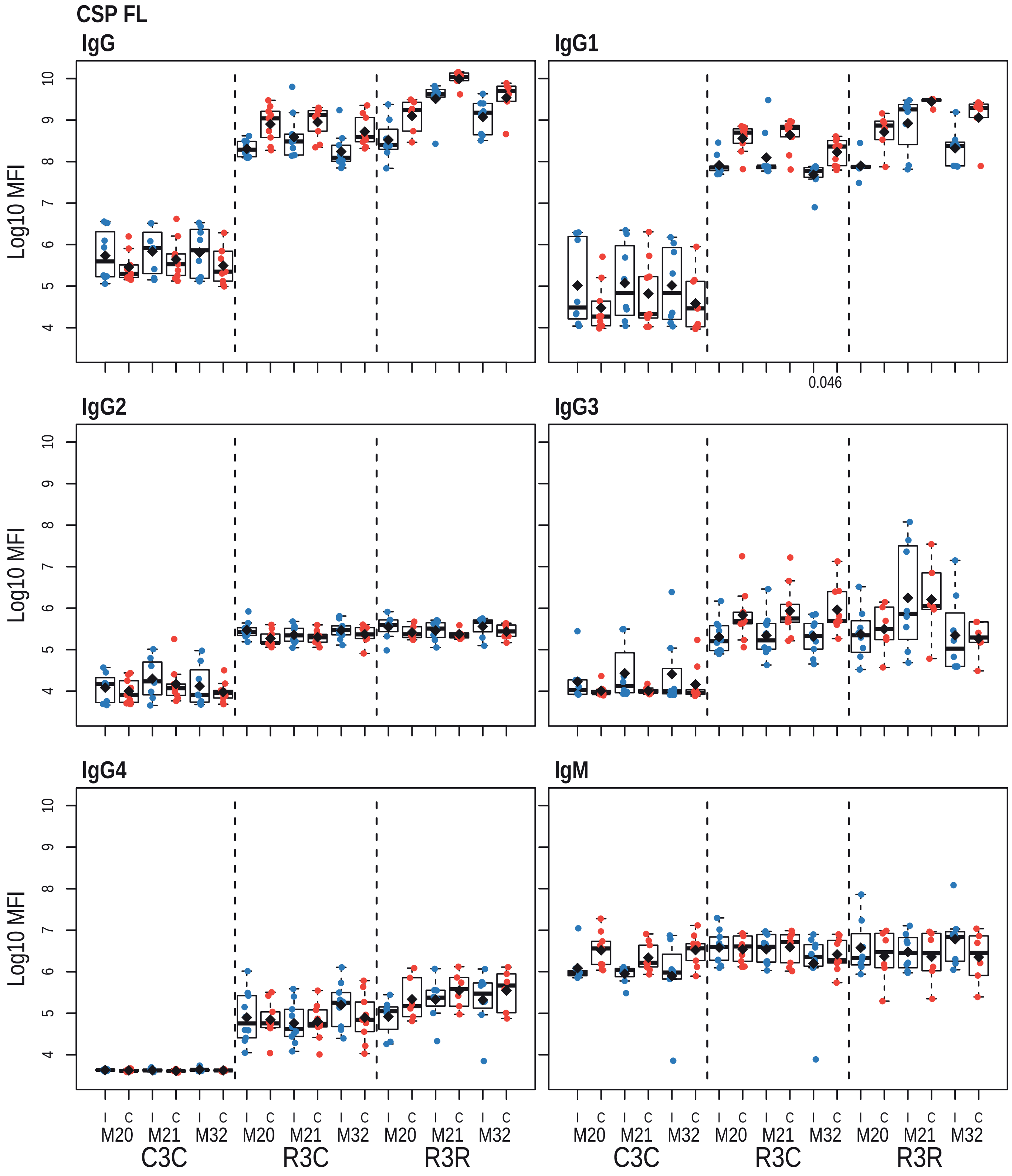

Supplementary Figure 3. RTS,S/AS01E booster and long-term immunogenicity stratified by age: Total IgG, IgG1-4 subclasses and IgM for CSP FL at month (M)20, M21 and M32 for RTS,S/AS01 vaccinees with (R3R) and without (R3C) booster, and comparator (C3C). Stratified analysis by age, children (C) 5-17 months (red) and infants (I) 6-12 weeks (blue) at the moment of first vaccination. Boxplots with medians, interquartile ranges (IQR), upper whisker as the smallest between maximum x value and Q3 + 1.5 * IQR, lower whisker as the largest between minimum x value and Q1 – 1.5 * IQR, and log10(geometric mean(MFI)) (diamond). Non-parametric tests were used to compare the booster response (M20 vs M21), the long-term immunogenicity (M21 vs M32), and the effect of age (children vs infants). P values are reported in supplementary tables. The y axis is in logarithm 10 scale. R3R: three doses of RTS,S/AS01E and a RTS,S/AS01E booster. R3C: three doses of RTS,S/AS01E and a comparator booster. C3C: three doses of a comparator vaccine and a comparator booster.

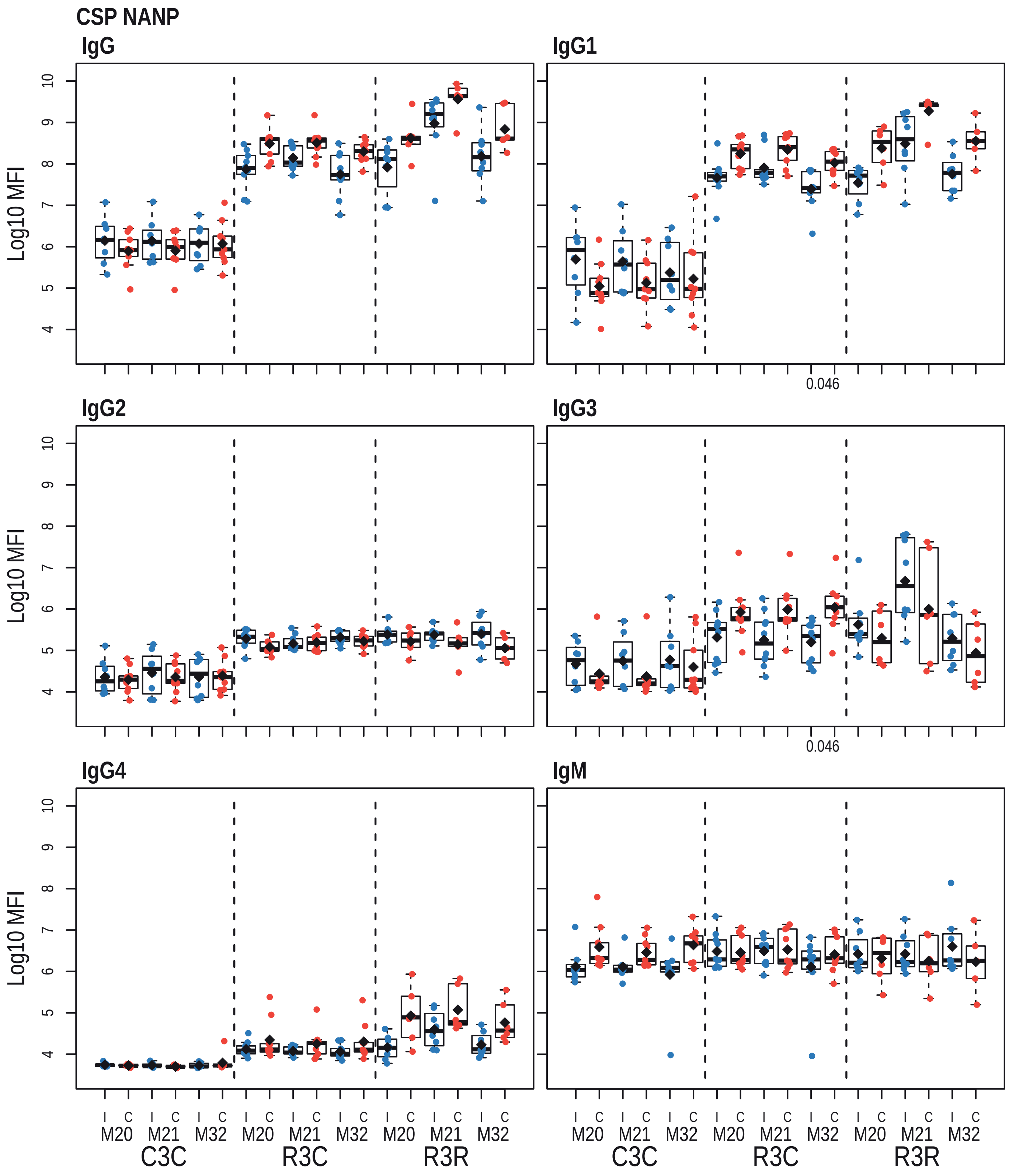

Supplementary Figure 4. RTS,S/AS01E booster and long-term immunogenicity stratified by age: Total IgG, IgG1-4 subclasses and IgM for CSP NANP at month (M)20, M21 and M32 for RTS,S/AS01 vaccinees with (R3R) and without (R3C) booster, and comparator (C3C). Stratified analysis by age, children (C) 5-17 months (red) and infants (I) 6-12 weeks (blue) at the moment of first vaccination. Boxplots with medians, interquartile ranges (IQR), upper whisker as the smallest between maximum x value and Q3 + 1.5 * IQR, lower whisker as the largest between minimum x value and Q1 – 1.5 * IQR, and log10(geometric mean(MFI)) (diamond). Non-parametric tests were used to compare the booster response (M20 vs M21), the long-term immunogenicity (M21 vs M32), and the effect of age (children vs infants). P values are reported in supplementary tables. The y axis is in logarithm 10 scale. R3R: three doses of RTS,S/AS01E and a RTS,S/AS01E booster. R3C: three doses of RTS,S/AS01E and a comparator booster. C3C: three doses of a comparator vaccine and a comparator booster.

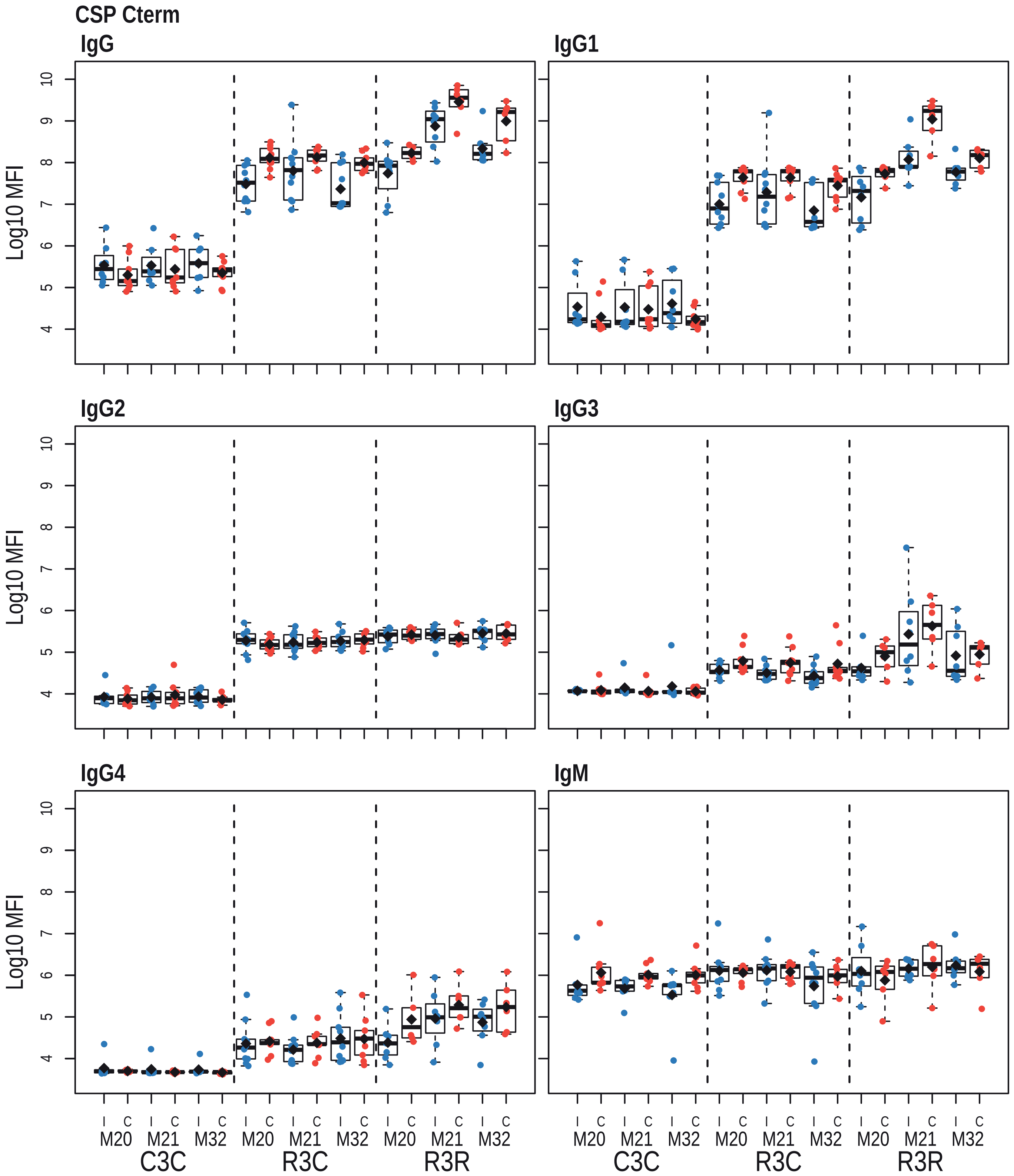

Supplementary Figure 5. RTS,S/AS01E booster and long-term immunogenicity stratified by age: Total IgG, IgG1-4 subclasses and IgM for CSP Cterm at month (M)20, M21 and M32 for RTS,S/AS01 vaccinees with (R3R) and without (R3C) booster, and comparator (C3C). Stratified analysis by age, children (C) 5-17 months (red) and infants (I) 6-12 weeks (blue) at the moment of first vaccination. Boxplots with medians, interquartile ranges (IQR), upper whisker as the smallest between maximum x value and Q3 + 1.5 * IQR, lower whisker as the largest between minimum x value and Q1 – 1.5 * IQR, and log10(geometric mean(MFI)) (diamond). Non-parametric tests were used to compare the booster response (M20 vs M21), the long-term immunogenicity (M21 vs M32), and the effect of age (children vs infants). P values are reported in supplementary tables. The y axis is in logarithm 10 scale. R3R: three doses of RTS,S/AS01E and a RTS,S/AS01E booster. R3C: three doses of RTS,S/AS01E and a comparator booster. C3C: three doses of a comparator vaccine and a comparator booster.

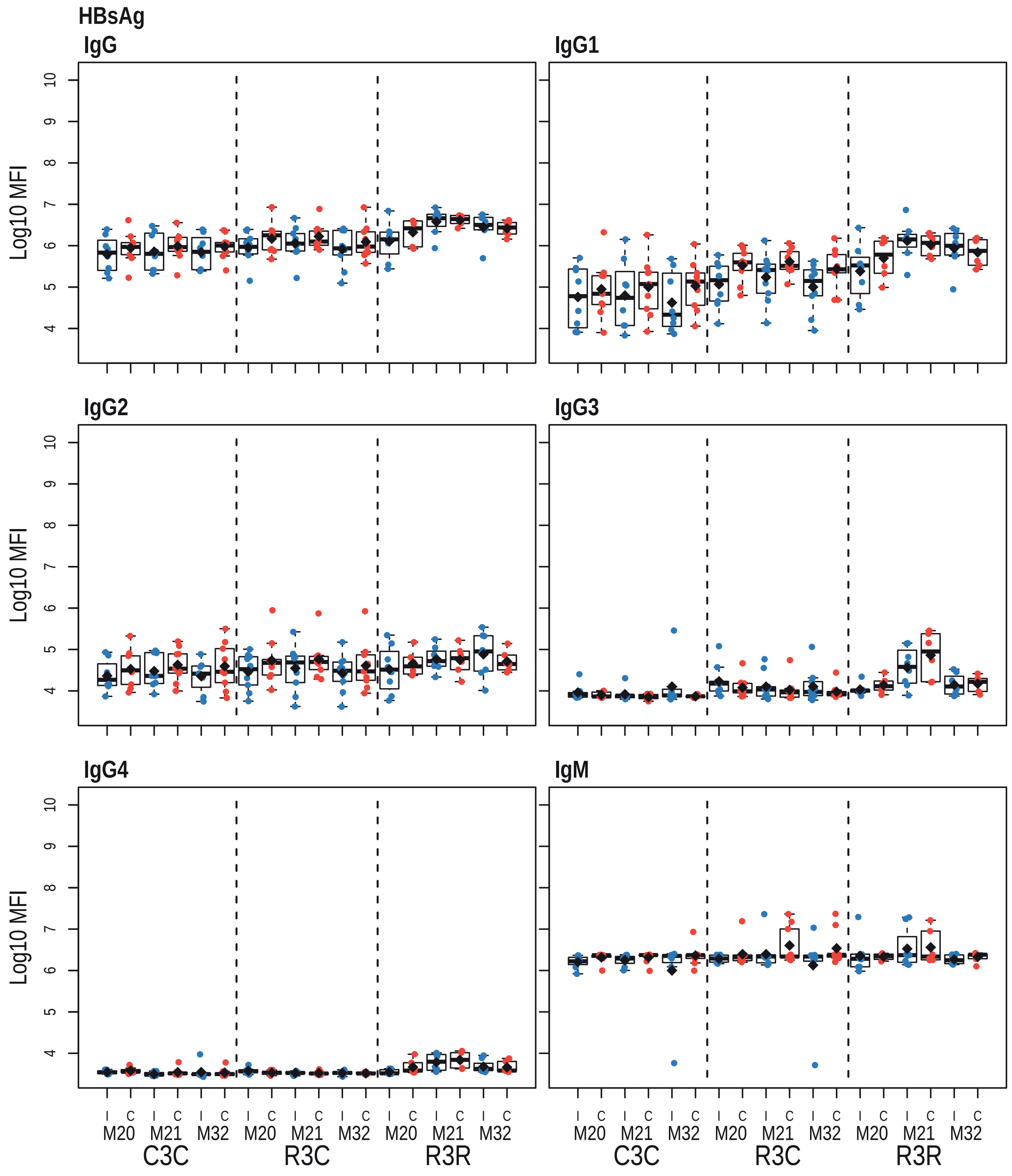

Supplementary Figure 6. RTS,S/AS01E booster and long-term immunogenicity stratified by age: Total IgG, IgG1-4 subclasses and IgM for HBsAg at month (M)20, M21 and M32 for RTS,S/AS01 vaccinees with (R3R) and without (R3C) booster, and comparator (C3C). Stratified analysis by age, children (C) 5-17 months (red) and infants (I) 6-12 weeks (blue) at the moment of first vaccination. Boxplots with medians, interquartile ranges (IQR), upper whisker as the smallest between maximum x value and Q3 + 1.5 * IQR, lower whisker as the largest between minimum x value and Q1 – 1.5 * IQR, and log10(geometric mean(MFI)) (diamond). Non-parametric tests were used to compare the booster response (M20 vs M21), the long-term immunogenicity (M21 vs M32), and the effect of age (children vs infants). P values are reported in supplementary tables. The y axis is in logarithm 10 scale. R3R: three doses of RTS,S/AS01E and a RTS,S/AS01E booster. R3C: three doses of RTS,S/AS01E and a comparator booster. C3C: three doses of a comparator vaccine and a comparator booster.

Supplementary Table 3. Immunogenicity to vaccine antigens stratified by previous clinical malaria and by clinical malaria after a booster dose (M21): Non-parametric test p-values for comparison of levels with or without clinical malaria (M vs NM) for IgG, IgG1-4 subclasses and IgM at month (M)20, 21 and 32 for RTS,S/AS01 vaccinees with (R3R) and without (R3C) booster, and comparator (C3C). P-values <0.05 are highlighted in yellow. P-values were adjusted for multiple comparisons, but none was significant and data are not shown.

		Prebooster cases: Malaria vs No Malaria								
		C3C			R3C			R3R		
Isotype	Antigen	M20	M21	M32	M20	M21	M32	M20	M21	M32
IgG	CSP_FL	0.06	0.30	0.045	0.07	0.07	0.14	0.70	0.44	0.90
IgG	Cterm	0.87	0.16	0.87	0.11	0.07	0.02	0.61	0.30	0.44
IgG	HBsAg	0.55	0.70	0.25	0.02	0.01	0.01	0.70	0.61	0.70
IgG	NANP	0.41	0.55	0.35	0.57	0.42	0.35	0.61	0.90	0.70
IgG1	CSP_FL	0.25	0.55	0.30	0.14	0.14	0.14	0.61	0.15	0.70
IgG1	Cterm	0.78	0.06	0.70	0.07	0.047	0.29	0.80	0.06	0.19
IgG1	HBsAg	0.62	0.87	0.48	0.02	0.01	0.01	0.44	0.80	0.70
IgG1	NANP	0.1	0.70	0.20	0.29	0.19	0.11	0.90	0.52	0.90
IgG2	CSP_FL	<0.001	0.08	0.006	0.84	0.95	0.35	0.44	1	0.61
IgG2	Cterm	1	0.70	0.19	0.35	0.14	0.42	0.30	0.61	0.90
IgG2	HBsAg	0.96	0.62	0.30	0.75	0.65	0.07	0.44	0.90	0.70
IgG2	NANP	0.045	0.30	0.16	0.65	0.55	0.57	0.90	0.36	0.30
IgG3	CSP_FL	0.02	0.08	0.16	0.07	0.19	0.23	1	0.61	0.84
IgG3	Cterm	0.19	0.78	0.30	0.84	0.84	0.75	0.52	0.44	1
IgG3	HBsAg	0.008	0.07	0.73	0.57	0.46	0.01	1	0.44	0.80
IgG3	NANP	0.16	0.70	0.48	0.65	0.65	0.29	1	0.90	0.61
IgG4	CSP_FL	0.46	0.09	0.17	0.95	0.75	0.65	0.24	0.30	0.19
IgG4	Cterm	0.28	0.57	0.23	0.65	0.57	0.84	0.11	0.70	0.24
IgG4	HBsAg	0.13	0.19	0.82	0.26	0.89	0.39	0.31	0.29	0.59
IgG4	NANP	0.95	0.53	0.73	0.95	0.95	0.75	0.30	0.06	0.30
IgM	CSP_FL	0.62	0.30	0.87	0.42	0.42	0.35	0.80	0.30	0.70
IgM	Cterm	0.30	0.35	0.35	0.95	0.84	1	0.80	0.24	0.61
IgM	HBsAg	0.48	0.1	1	0.65	0.11	0.42	0.90	0.24	0.80
IgM	NANP	0.78	0.96	0.70	0.42	0.49	0.29	0.36	0.15	0.30

		Postbooster cases: Malaria vs No Malaria								
		C3C			R3C			R3R		
Isotype	Antigen	M20	M21	M32	M20	M21	M32	M20	M21	M32
IgG	CSP_FL	0.36	0.86	0.09	0.81	0.36	0.53	0.64	0.54	0.45
IgG	Cterm	0.43	0.07	0.86	0.89	0.18	0.60	0.37	0.45	0.14
IgG	HBsAg	0.51	0.36	0.30	0.53	0.74	0.47	0.73	0.14	0.73
IgG	NANP	0.43	0.43	0.77	0.81	0.36	0.60	0.95	0.95	0.84
IgG1	CSP_FL	0.20	0.68	0.047	0.41	0.18	0.47	0.95	0.64	0.37
IgG1	Cterm	0.95	0.20	0.77	0.89	0.31	0.66	0.54	0.30	0.19
IgG1	HBsAg	0.59	0.66	0.68	0.22	0.96	1	0.37	0.19	0.45
IgG1	NANP	1	0.43	0.43	0.41	0.74	0.36	0.95	1	1
IgG2	CSP_FL	0.16	0.86	0.047	0.53	0.89	0.26	0.64	0.64	0.54
IgG2	Cterm	0.38	0.30	0.57	0.81	0.81	0.74	0.54	0.30	0.11
IgG2	HBsAg	0.36	0.24	0.20	0.66	0.89	0.31	0.37	0.04	0.14
IgG2	NANP	0.86	0.68	0.86	0.96	0.19	0.1	0.64	0.002	0.004
IgG3	CSP_FL	0.07	0.85	0.02	0.26	0.22	0.89	0.95	0.19	0.14
IgG3	Cterm	0.90	1	0.12	0.31	0.53	0.60	0.45	0.14	0.19
IgG3	HBsAg	0.09	0.34	0.90	0.66	0.73	1	0.19	0.054	0.02
IgG3	NANP	1	0.68	0.51	0.36	0.18	1	0.95	0.45	0.64
IgG4	CSP_FL	0.07	0.75	0.34	0.08	0.36	0.1	0.45	0.45	0.45
IgG4	Cterm	0.34	0.19	0.21	0.08	0.53	0.22	0.73	0.64	0.30
IgG4	HBsAg	0.05	0.80	0.75	0.65	0.51	0.88	0.78	0.32	0.72
IgG4	NANP	1	0.57	0.95	0.89	0.74	0.96	0.95	0.73	0.84
IgM	CSP_FL	0.95	0.36	1	0.41	0.15	1	0.19	1	0.84
IgM	Cterm	0.51	0.36	0.77	0.26	0.08	0.96	0.054	1	0.64
IgM	HBsAg	0.68	0.77	0.77	0.22	0.81	1	0.84	0.54	0.64
IgM	NANP	0.95	0.95	0.95	0.47	0.1	0.89	0.11	0.64	0.54

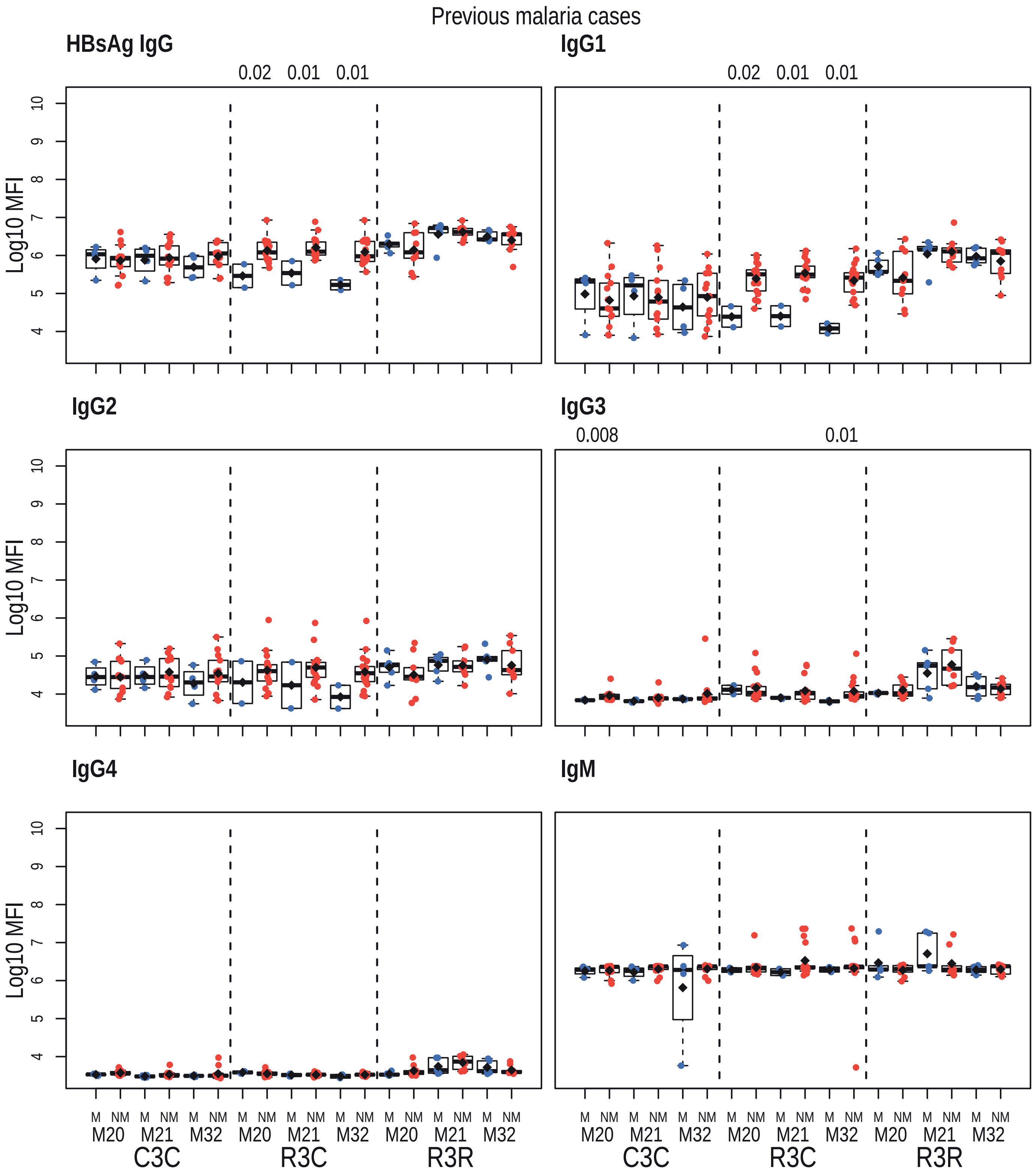

Supplementary Figure 7. Immunogenicity to HBsAg stratified by previous clinical malaria: IgG, IgG1-4 subclasses and IgM at month (M)20, 21 and 32 for RTS,S/AS01 vaccinees with (R3R) and without (R3C) booster, and comparator (C3C). Stratified analysis by malaria cases before M20, subjects who presented with clinical malaria (M=blue) and subjects without malaria (NM=red). Boxplots with medians, interquartile ranges (IQR), upper whisker as the smallest between maximum x value and Q3 + 1.5 * IQR, lower whisker as the largest between minimum x value and Q1 – 1.5 * IQR, and log10(geometric mean(MFI)) (diamond). Non-parametric tests were used to compare levels with or without clinical malaria (M vs NM). P-values were adjusted for multiple comparisons, but none was significant. Only p-values <0.05 before adjustment are shown. The y axis is in logarithm 10 scale. R3R: three doses of RTS,S/AS01E and a RTS,S/AS01E booster. R3C: three doses of RTS,S/AS01E and a comparator booster. C3C: three doses of a comparator vaccine and a comparator booster.

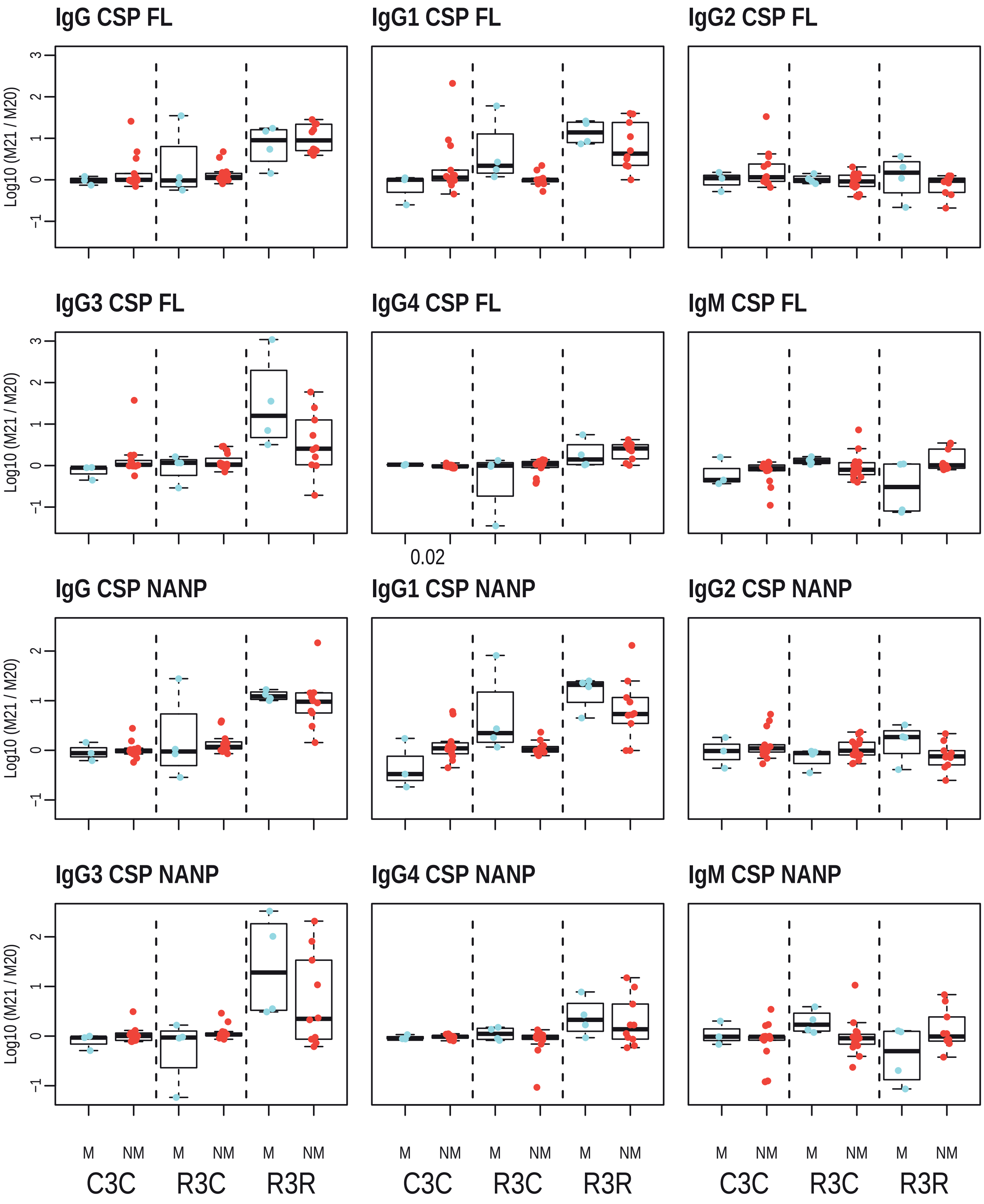

Supplementary Figure 8. Fold change in immunogenicity against vaccine antigens from month (M) 20 to M21 stratified by clinical malaria after M21: Antibody response, IgG, IgG1-4 subclasses and IgM, for CSP FL and CSP NANP, for RTS,S/AS01 vaccinees with (R3R) and without (R3C) booster, and comparator (C3C). Stratified analysis by malaria after M21, subjects who presented with clinical malaria (M=blue) and subjects without malaria cases (NM=red). Boxplots with medians, interquartile ranges (IQR), upper whisker as the smallest between maximum x value and Q3 + 1.5 * IQR, lower whisker as the largest between minimum x value and Q1 – 1.5 * IQR, and log10(geometric mean(MFI)) (diamond). Non-parametric tests were used to compare fold change with or without clinical malaria. P-values were adjusted for multiple comparisons, but none was significant. Only p-values <0.05 before adjustment are shown. R3R: three doses of RTS,S/AS01E and a RTS,S/AS01E booster. R3C: three doses of RTS,S/AS01E and a comparator booster. C3C: three doses of a comparator vaccine and a comparator booster.

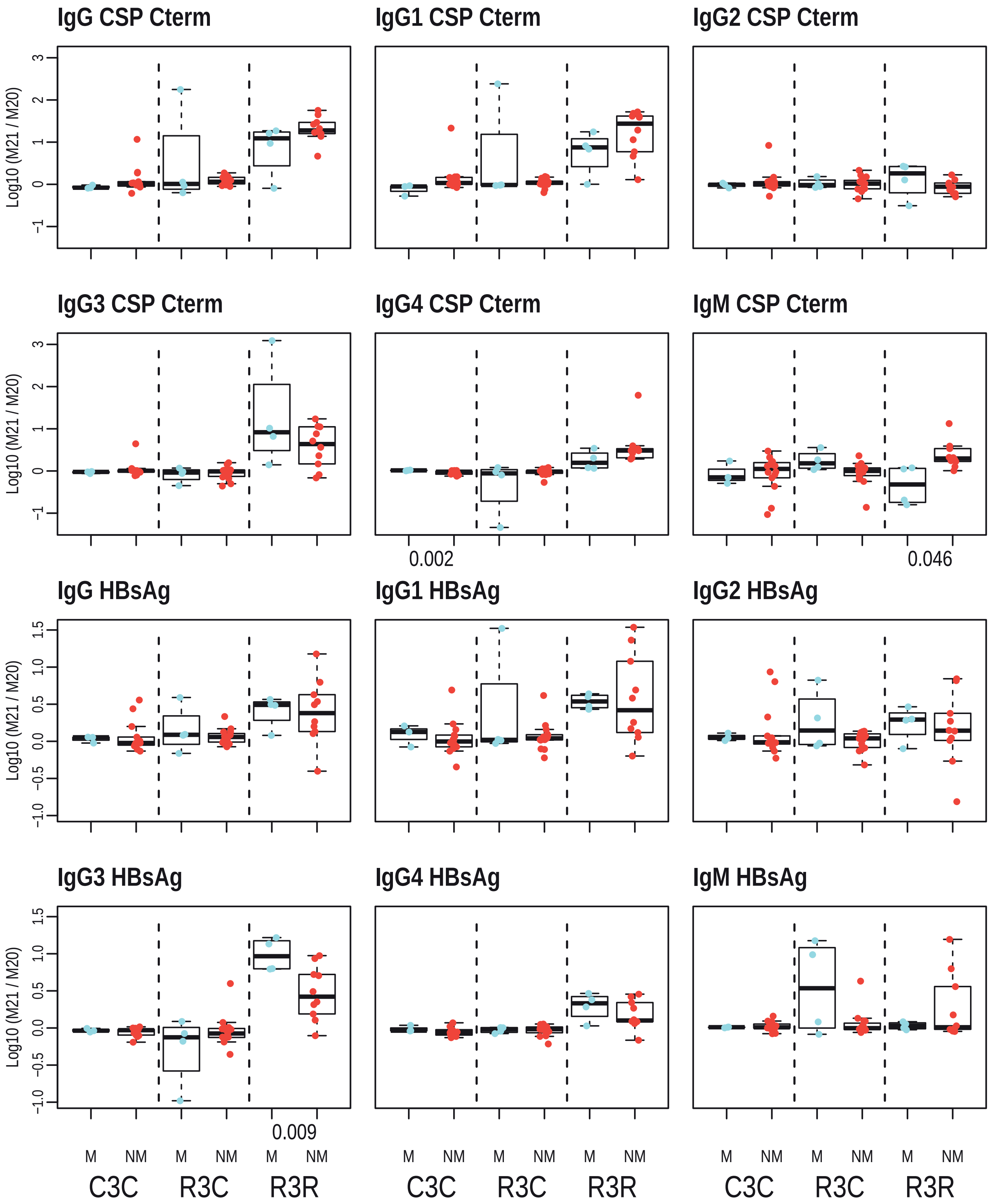

Supplementary Figure 9. Fold change in immunogenicity against vaccine antigens from month (M) 20 to M21 stratified by clinical malaria after M21: Antibody response, IgG, IgG1-4 subclasses and IgM, for CSP Cterm and HBsAg, for RTS,S/AS01 vaccinees with (R3R) and without (R3C) booster, and comparator (C3C). Stratified analysis by malaria after M21, subjects who presented with clinical malaria (M=blue) and subjects without malaria cases (NM=red). Boxplots with medians, interquartile ranges (IQR), upper whisker as the smallest between maximum x value and Q3 + 1.5 * IQR, lower whisker as the largest between minimum x value and Q1 – 1.5 * IQR, and log10(geometric mean(MFI)) (diamond). Non-parametric tests were used to compare fold change with or without clinical malaria. P-values were adjusted for multiple comparisons, but none was significant. Only p-values <0.05 before adjustment are shown. R3R: three doses of RTS,S/AS01E and a RTS,S/AS01E booster. R3C: three doses of RTS,S/AS01E and a comparator booster. C3C: three doses of a comparator vaccine and a comparator booster.

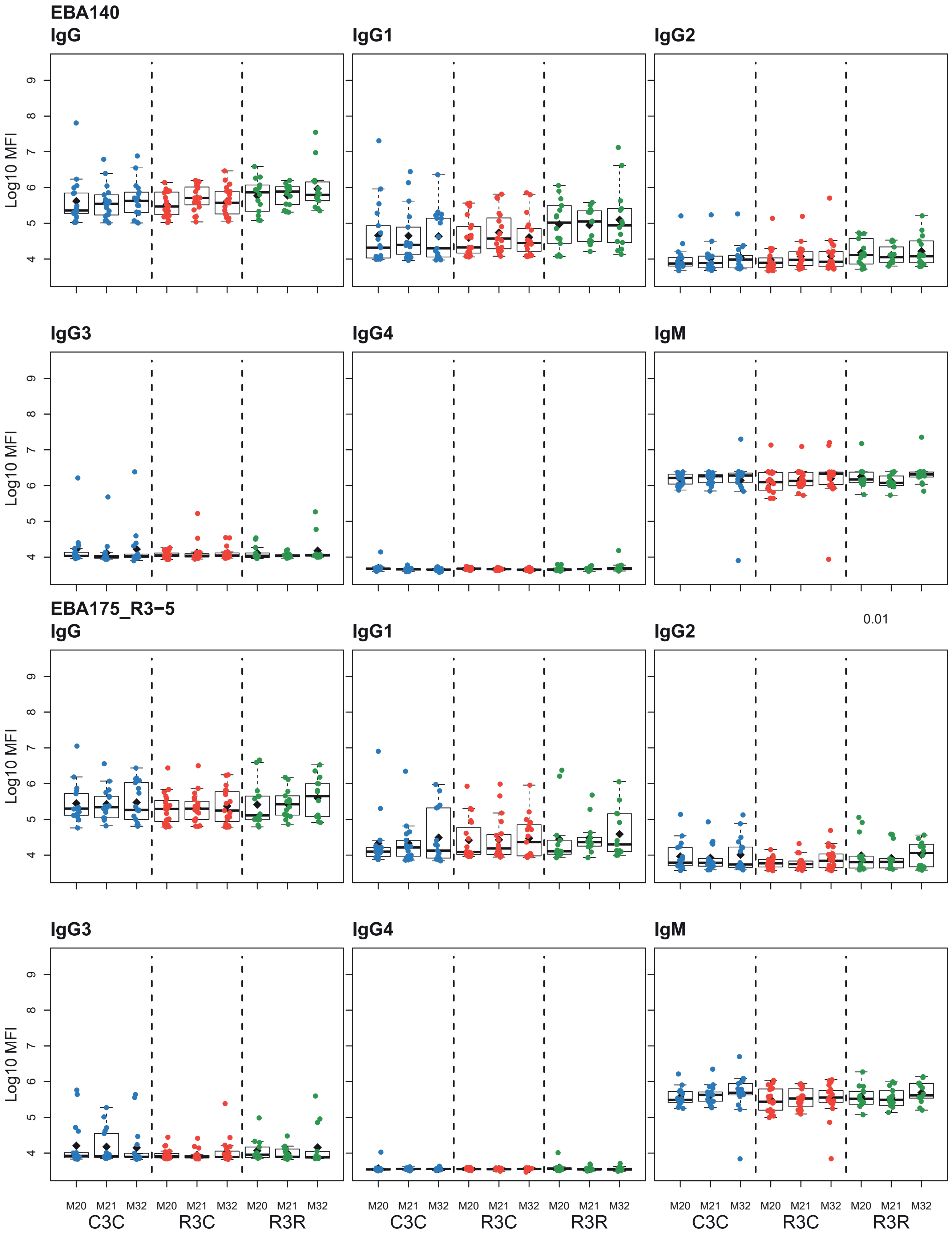

Supplementary Figure 10. RTS,S/AS01E booster and long-term immunogenicity against blood stage antigens: Total IgG, IgG1-4 subclasses and IgM for EBA140 and EBA175 R3-5 at month (M) 20, 21 and 32 for RTS,S/AS01 vaccinees with (R3R) and without (R3C) booster, and comparator (C3C). Boxplots with medians, interquartile ranges (IQR), upper whisker as the smallest between maximum x value and Q3 + 1.5 * IQR, lower whisker as the largest between minimum x value and Q1 – 1.5 * IQR, and log10(geometric mean(MFI)) (diamond). Non-parametric tests were used to compare the booster response (M20 vs M21) and the long-term immunogenicity (M21 vs M32), as well as to compare the R3C and R3R groups at each timepoint. P-values were not significant after adjustment for multiple testing. The y axis is in logarithm 10 scale. R3R (green): three doses of RTS,S/AS01E and a RTS,S/AS01E booster. R3C (red): three doses of RTS,S/AS01E and a comparator booster. C3C (blue): three doses of a comparator vaccine and a comparator booster.

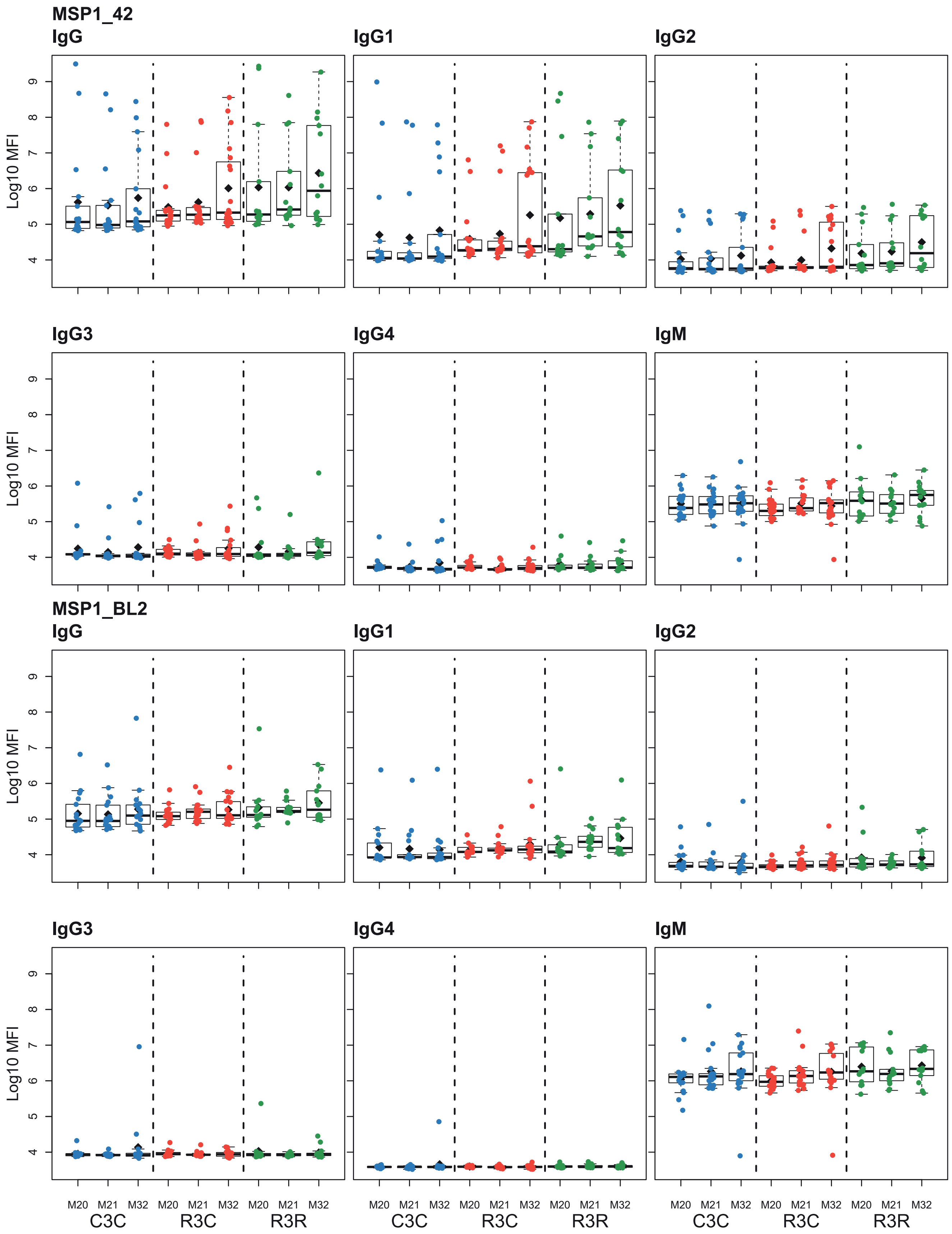

Supplementary Figure 11. RTS,S/AS01E booster and long-term immunogenicity against blood stage antigens: Total IgG, IgG1-4 subclasses and IgM for $MSP1_{42}$ and MSP1 Block2 at month (M) 20, 21 and 32 for RTS,S/AS01 vaccinees with (R3R) and without (R3C) booster, and comparator (C3C). Boxplots with medians, interquartile ranges (IQR), upper whisker as the smallest between maximum x value and Q3 + 1.5 * IQR, lower whisker as the largest between minimum x value and Q1 – 1.5 * IQR, and log10(geometric mean(MFI)) (diamond). Non-parametric tests were used to compare the booster response (M20 vs M21) and the long-term immunogenicity (M21 vs M32), as well as to compare the R3C and R3R groups at each timepoint. P-values were not significant after adjustment for multiple testing. The y axis is in logarithm 10 scale. R3R (green): three doses of RTS,S/AS01E and a RTS,S/AS01E booster. R3C (red): three doses of RTS,S/AS01E and a comparator booster. C3C (blue): three doses of a comparator vaccine and a comparator booster.

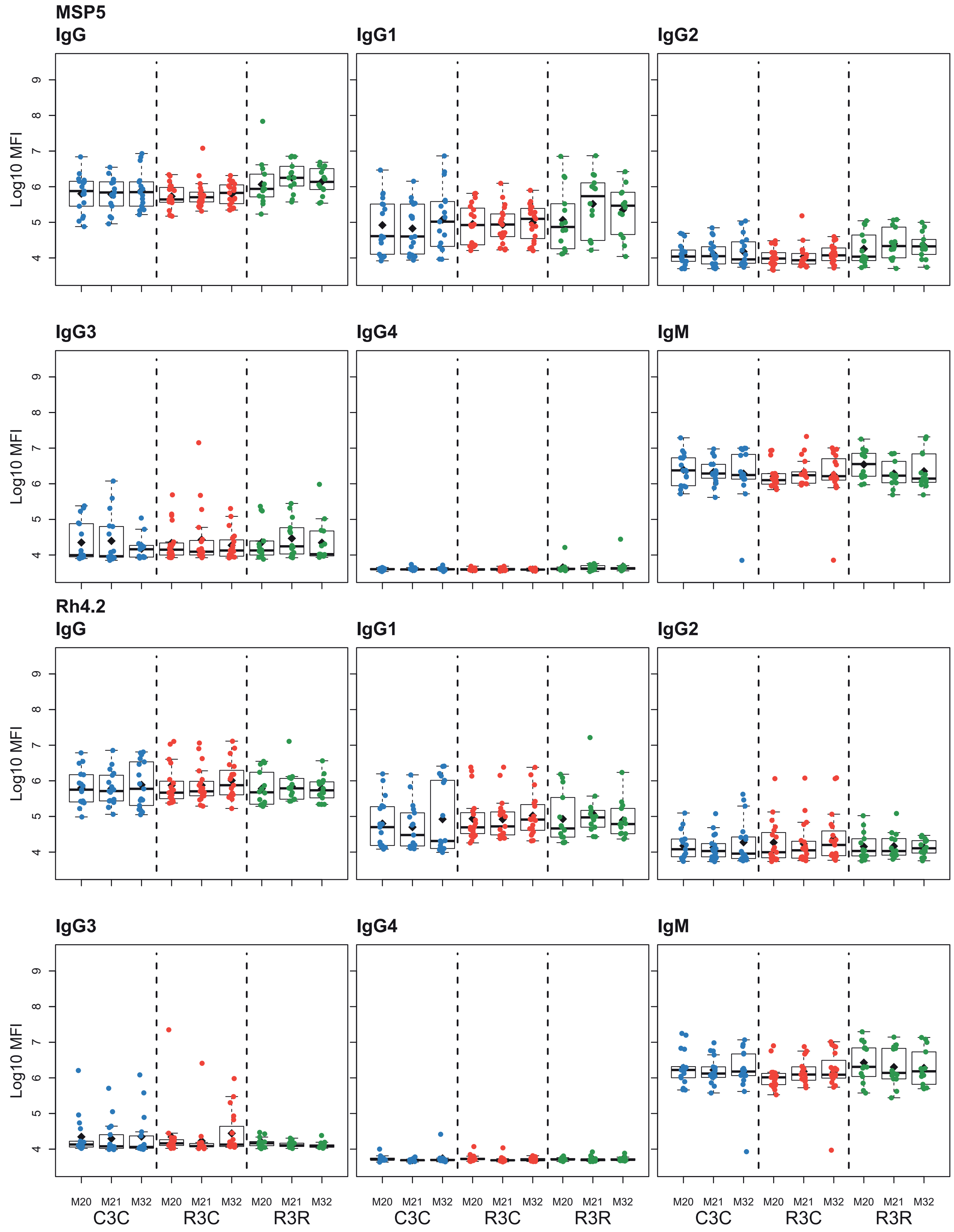

Supplementary Figure 12. RTS,S/AS01E booster and long-term immunogenicity against blood stage antigens: Total IgG, IgG1-4 subclasses and IgM for MSP5 and Rh4.2 at month (M) 20, 21 and 32 for RTS,S/AS01 vaccinees with (R3R) and without (R3C) booster, and comparator (C3C). Boxplots with medians, interquartile ranges (IQR), upper whisker as the smallest between maximum x value and Q3 + 1.5 * IQR, lower whisker as the largest between minimum x value and Q1 – 1.5 * IQR, and log10(geometric mean(MFI)) (diamond). Non-parametric tests were used to compare the booster response (M20 vs M21) and the long-term immunogenicity (M21 vs M32), as well as to compare the R3C and R3R groups at each timepoint. P-values were not significant after adjustment for multiple testing. The y axis is in logarithm 10 scale. R3R (green): three doses of RTS,S/AS01E and a RTS,S/AS01E booster. R3C (red): three doses of RTS,S/AS01E and a comparator booster. C3C (blue): three doses of a comparator vaccine and a comparator booster.

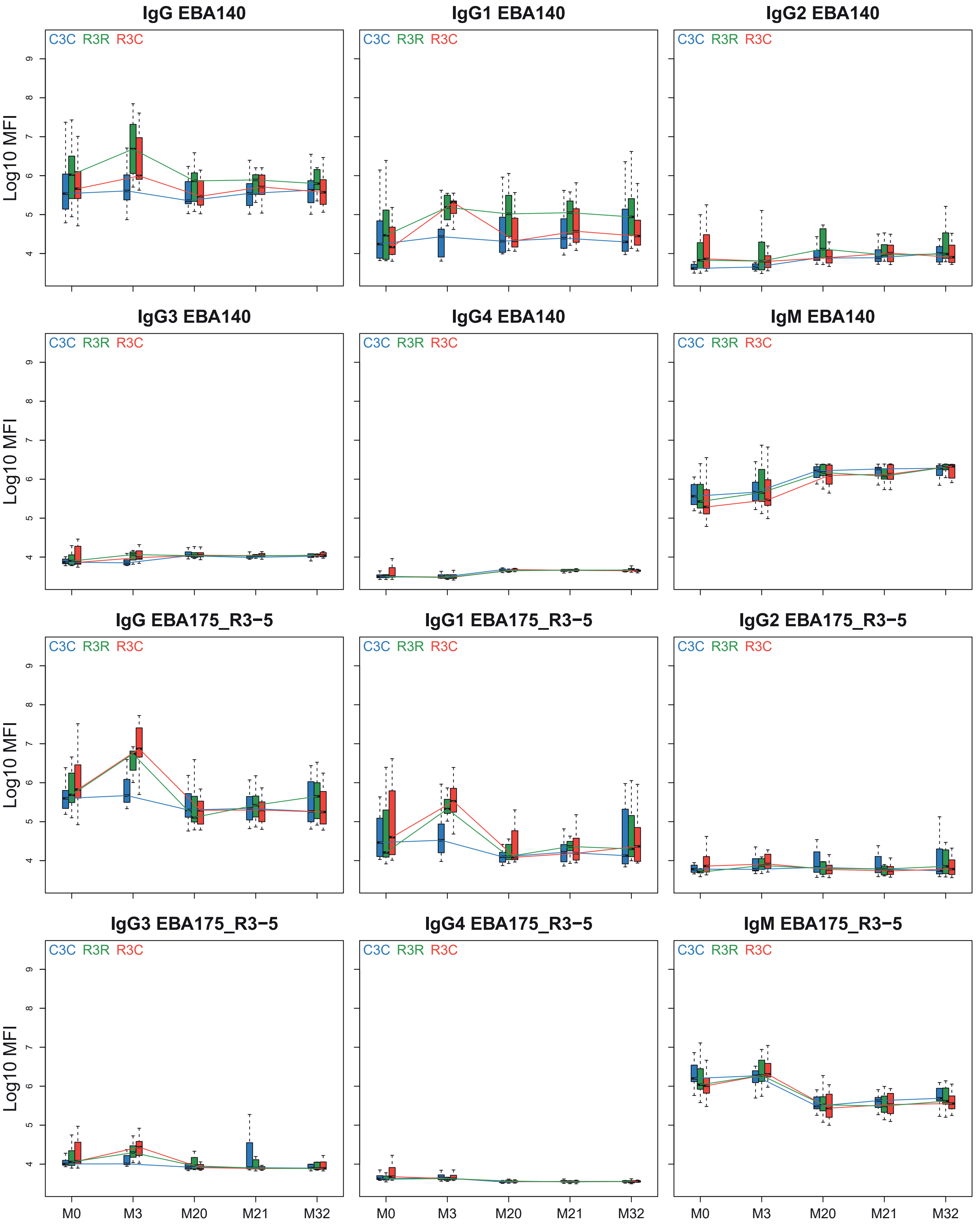

Supplementary Figure 13. Antibody responses against blood stage antigens (EBA140 and EBA175 R3-5) for months (M) 0, 3, 20, 21 and 32 for IgG, IgG1-4 and IgM. Boxplots with median and interquartile ranges. The y axis is in logarithm 10 scale. Data from M 0 and 3 were obtained from a previous study in the same individuals [5], thus a batch effect might be present. R3R (green): three doses of RTS,S/AS01E and a RTS,S/AS01E booster at month 20. R3C (red): three doses of RTS,S/AS01E and a comparator booster. C3C (blue): three doses of a comparator vaccine and a comparator booster.

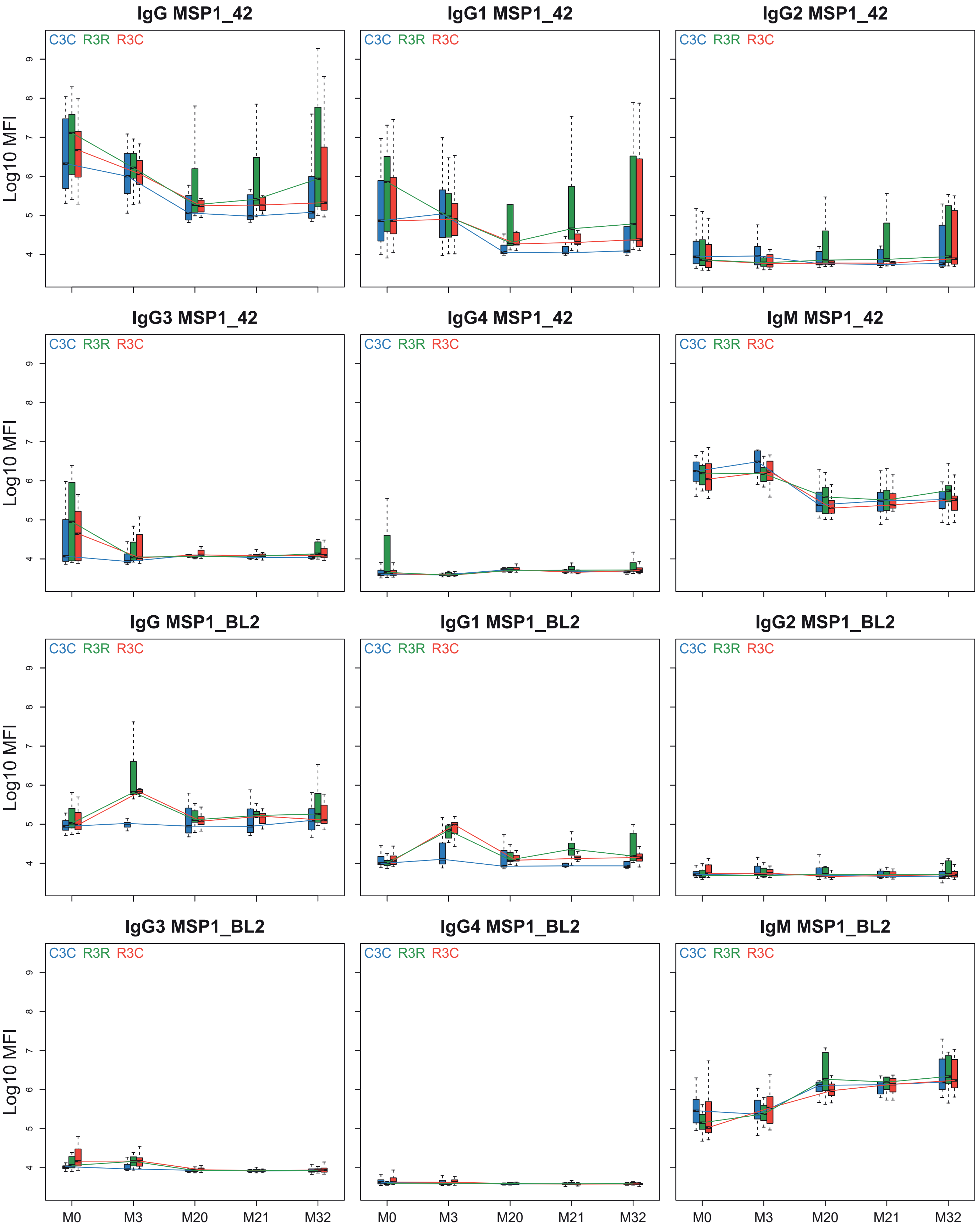

Supplementary Figure 14. Antibody responses against blood stage antigens ($MSP1_{42}$ and MSP1 Block2) for months (M) 0, 3, 20, 21 and 32 for IgG, IgG1-4 and IgM. Boxplots with median and interquartile ranges. The y axis is in logarithm 10 scale. Data from M 0 and 3 were obtained from a previous study in the same individuals [5], thus a batch effect might be present. R3R (green): three doses of RTS,S/AS01E and a RTS,S/AS01E booster at month 20. R3C (red): three doses of RTS,S/AS01E and a comparator booster. C3C (blue): three doses of a comparator vaccine and a comparator booster.

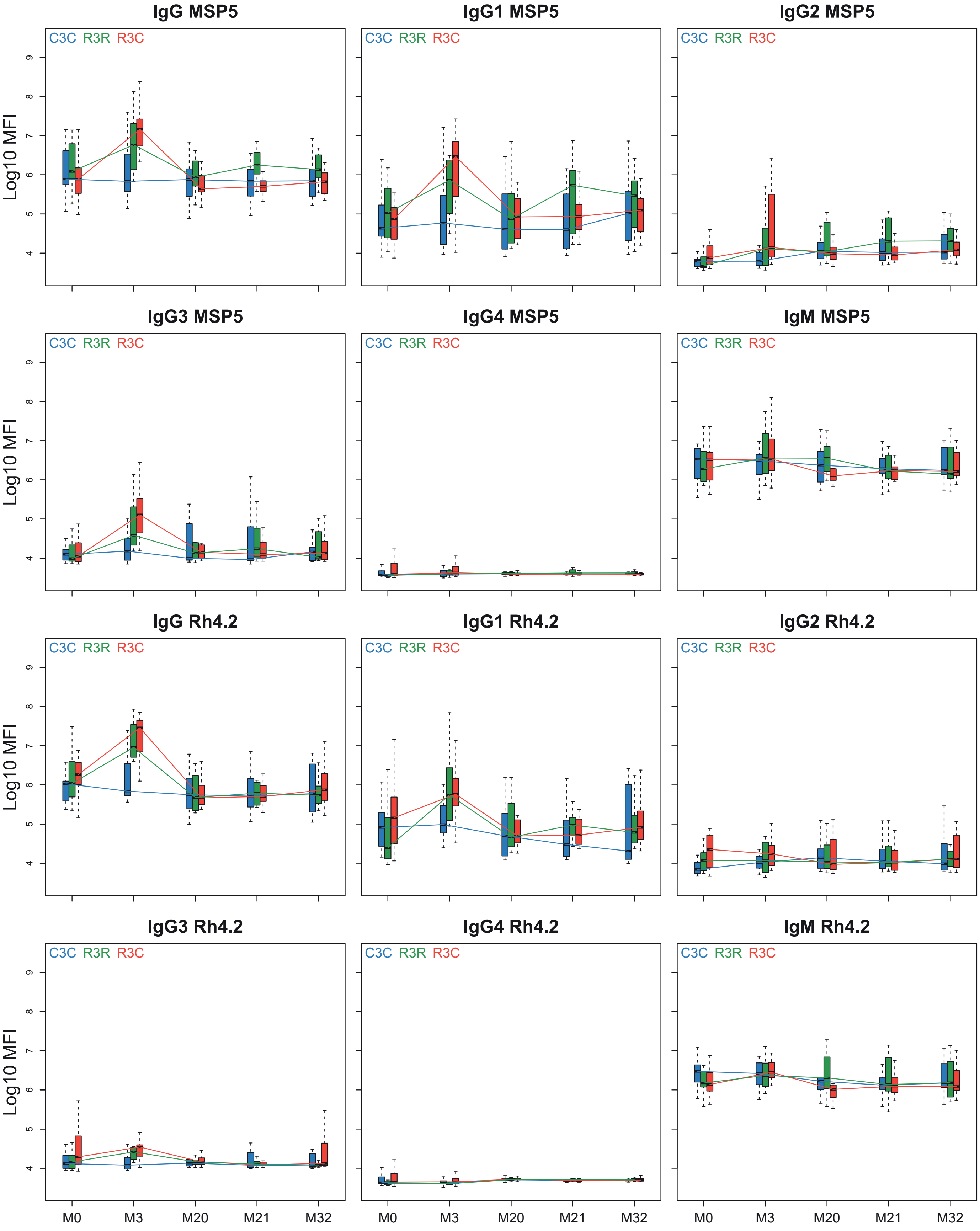

Supplementary Figure 15. Antibody responses against blood stage antigens (MSP5 and Rh4.2) for months (M) 0, 3, 20, 21 and 32 for IgG, IgG1-4 and IgM. Boxplots with median and interquartile ranges. The y axis is in logarithm 10 scale. Data from M 0 and 3 were obtained from a previous study in the same individuals [5], thus a batch effect might be present. R3R (green): three doses of RTS,S/AS01E and a RTS,S/AS01E booster at month 20. R3C (red): three doses of RTS,S/AS01E and a comparator booster. C3C (blue): three doses of a comparator vaccine and a comparator booster.

Supplementary Table 4. RTS,S/AS01E booster and long-term immunogenicity against Plasmodium falciparum blood stage antigens: Comparisons of overall antibody levels between vaccination groups and timepoints. Total IgG, IgG1-4 subclasses and IgM for RTS,S/AS01 vaccinees with (R3R) and without (R3C) booster, and comparator (C3C). Non-parametric tests were used to compare the booster response (M20 vs M21) and the long-term immunogenicity (M21 vs M32), as well as to compare the R3C and R3R groups at each timepoint. Adjusted p values are shown in parenthesis. P-values <0.05 before adjustment are highlighted in yellow and P-values <0.001 before adjustment in green. R3R: three doses of RTS,S/AS01E and a RTS,S/AS01E booster. R3C: three doses of RTS,S/AS01E and a comparator booster. C3C: three doses of a comparator vaccine and a comparator booster.

		Comparison between vaccination groups					Comparison Time points		
		R3R vs R3C			R3R vs C3C	R3C vs C3C	M20vsM21	M21vsM32	
**Isotype**	**Antigen**	**M20**	**M21**	**M32**	**M32**	**M32**	**R3R**	**R3R**	**R3C**
IgG	EBA140	1	1	1	1	1	1	1	1
IgG	EBA175_R3−5	1	1	1	1	1	1	1	1
IgG	MSP1_42	1	1	1	1	1	1	1	1
IgG	MSP1_BL2	1	1	1	1	1	0.33	1	1
IgG	MSP5	1	0.055	0.45	1	1	1	1	1
IgG	Rh4.2	1	1	1	1	1	1	1	0.65
IgG	Rh5	1	<0.001	1	0.13	0.64	0.008	0.33	0.93
IgG1	EBA140	0.56	0.56	0.31	0.33	0.87	0.95	1	0.86
IgG1	EBA175_R3−5	1	0.58	0.58	0.58	0.80	0.69	1	1
IgG1	MSP1_42	0.95	0.11	0.58	0.11	0.18	0.69	1	0.86
IgG1	MSP1_BL2	0.95	0.07	0.62	0.051	0.09	0.28	0.92	1
IgG1	MSP5	1	0.26	0.43	0.58	1	0.69	0.84	1
IgG1	Rh4.2	0.83	0.73	0.83	0.58	0.56	0.80	0.80	0.73
IgG1	Rh5	0.83	0.001	0.11	0.003	0.003	0.01	0.03	0.17
IgG2	EBA140	0.33	0.80	0.55	0.55	1	0.80	1	1
IgG2	EBA175_R3−5	0.87	0.65	0.58	0.92	0.85	0.80	0.86	0.80
IgG2	MSP1_42	0.33	0.11	0.55	0.21	0.56	0.86	0.86	0.69
IgG2	MSP1_BL2	0.21	0.80	0.75	0.22	0.46	1	0.86	1
IgG2	MSP5	0.55	0.11	0.28	0.52	1	0.69	0.86	0.86
IgG2	Rh4.2	1	0.87	0.93	0.93	0.80	0.92	1	0.69
IgG2	Rh5	0.11	0.003	0.03	0.004	0.11	0.34	0.80	1
IgG3	EBA140	1	0.87	1	0.56	0.80	0.80	0.80	0.80
IgG3	EBA175_R3−5	0.56	0.73	1	0.95	0.88	0.80	1	0.86
IgG3	MSP1_42	0.56	1	0.80	0.33	0.56	0.92	0.69	0.83
IgG3	MSP1_BL2	0.58	0.80	1	1	1	1	0.92	1
IgG3	MSP5	1	0.73	1	0.58	0.70	1	0.69	0.83
IgG3	Rh4.2	0.96	0.80	0.22	0.85	0.33	0.72	0.86	0.69
IgG3	Rh5	0.58	0.11	0.62	1	0.56	0.69	0.41	0.80
IgG4	EBA140	0.31	1	0.18	0.33	0.87	0.86	0.80	0.69
IgG4	EBA175_R3−5	0.83	1	1	1	1	0.80	0.86	0.97
IgG4	MSP1_42	1	0.17	0.69	0.55	0.68	0.86	1	0.69
IgG4	MSP1_BL2	0.93	0.58	0.55	0.68	0.95	1	1	0.92
IgG4	MSP5	0.55	0.48	0.11	0.49	0.58	0.89	1	0.83
IgG4	Rh4.2	0.69	0.73	0.80	0.85	1	0.92	0.89	0.80
IgG4	Rh5	1	0.15	0.75	0.83	1	0.95	0.69	0.86
IgM	EBA140	1	1	1	1	1	0.01	0.17	1
IgM	EBA175_R3−5	1	1	1	1	1	1	0.99	1
IgM	MSP1_42	1	1	1	1	1	1	1	1
IgM	MSP1_BL2	0.49	1	1	1	1	1	1	1
IgM	MSP5	0.80	1	1	1	1	0.17	1	1
IgM	Rh4.2	0.86	1	1	1	1	0.24	1	1
IgM	Rh5	1	1	1	1	1	1	1	1

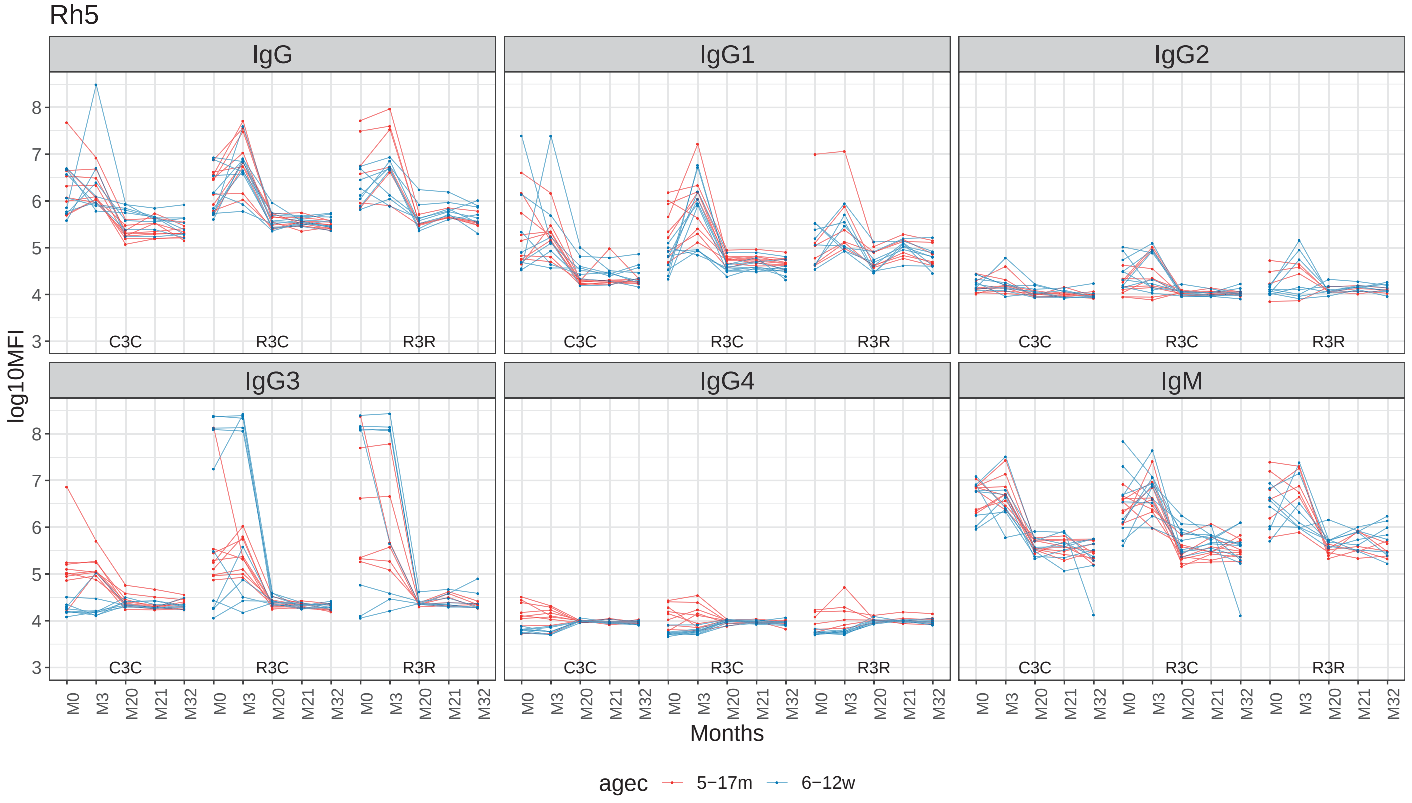

Supplementary Figure 16. Antibody responses against the blood stage antigen Rh5 for months (M) 0, 3, 20, 21 and 32 for IgG, IgG1, IgG2, IgG3, IgG4 and IgM. Line time plots connecting levels for each individual. The y axis is in logarithm 10 scale. Data from months 0 and 3 were obtained from a previous study in the same individuals [5], thus a batch effect might be present. R3R: three doses of RTS,S/AS01E and a RTS,S/AS01E booster at month 20. R3C: three doses of RTS,S/AS01E and a comparator booster. C3C: three doses of a comparator vaccine and a comparator booster. Children age 5-17 months are shown in orange and infants age 6-12 weeks old at first immunization are shown in blue.

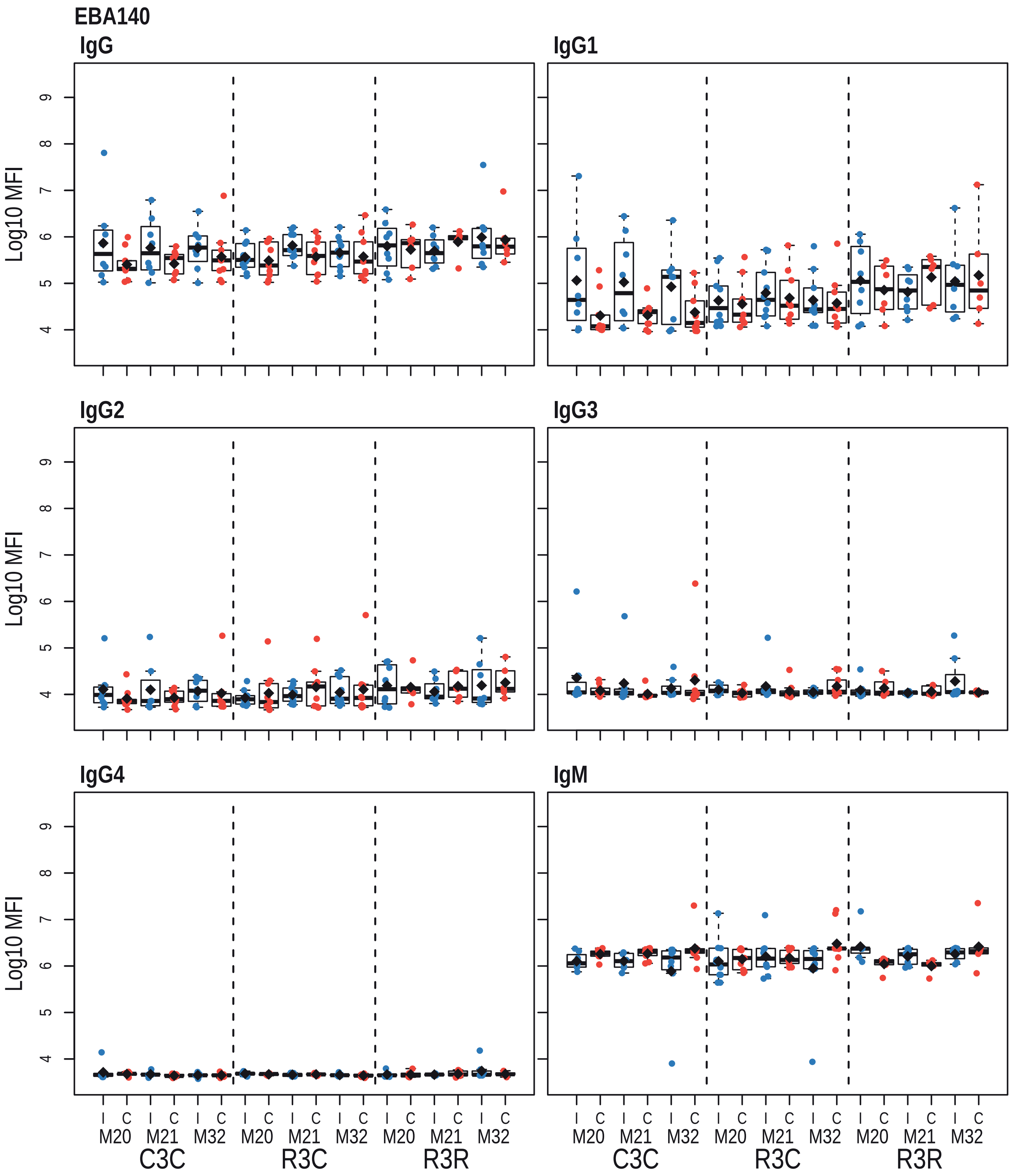

Supplementary Figure 17. RTS,S/AS01E booster and long-term immunogenicity stratified by age against blood stage antigens: IgG, IgG1-4 subclasses and IgM for EBA140 at month (M) 20, M21 and M32 for RTS,S/AS01 vaccinees with (R3R) and without (R3C) booster, and comparator (C3C). Stratified analysis by age, children (C) age 5-17 months (red) and infants (I) age 6-12 weeks (blue) at the time of first vaccination. Boxplots with medians, interquartile ranges (IQR), upper whisker as the smallest between maximum x value and Q3 + 1.5 * IQR, lower whisker as the largest between minimum x value and Q1 – 1.5 * IQR, and log10(geometric mean(MFI)) (diamond). Non-parametric tests were used to compare the booster response (M20 vs M21), the long-term immunogenicity (M21 vs M32), and the effect of age (children vs infants). P-values were not significant after adjustment for multiple testing. The y axis is in logarithm 10 scale. R3R: three doses of RTS,S/AS01E and a RTS,S/AS01E booster. R3C: three doses of RTS,S/AS01E and a comparator booster. C3C: three doses of a comparator vaccine and a comparator booster.

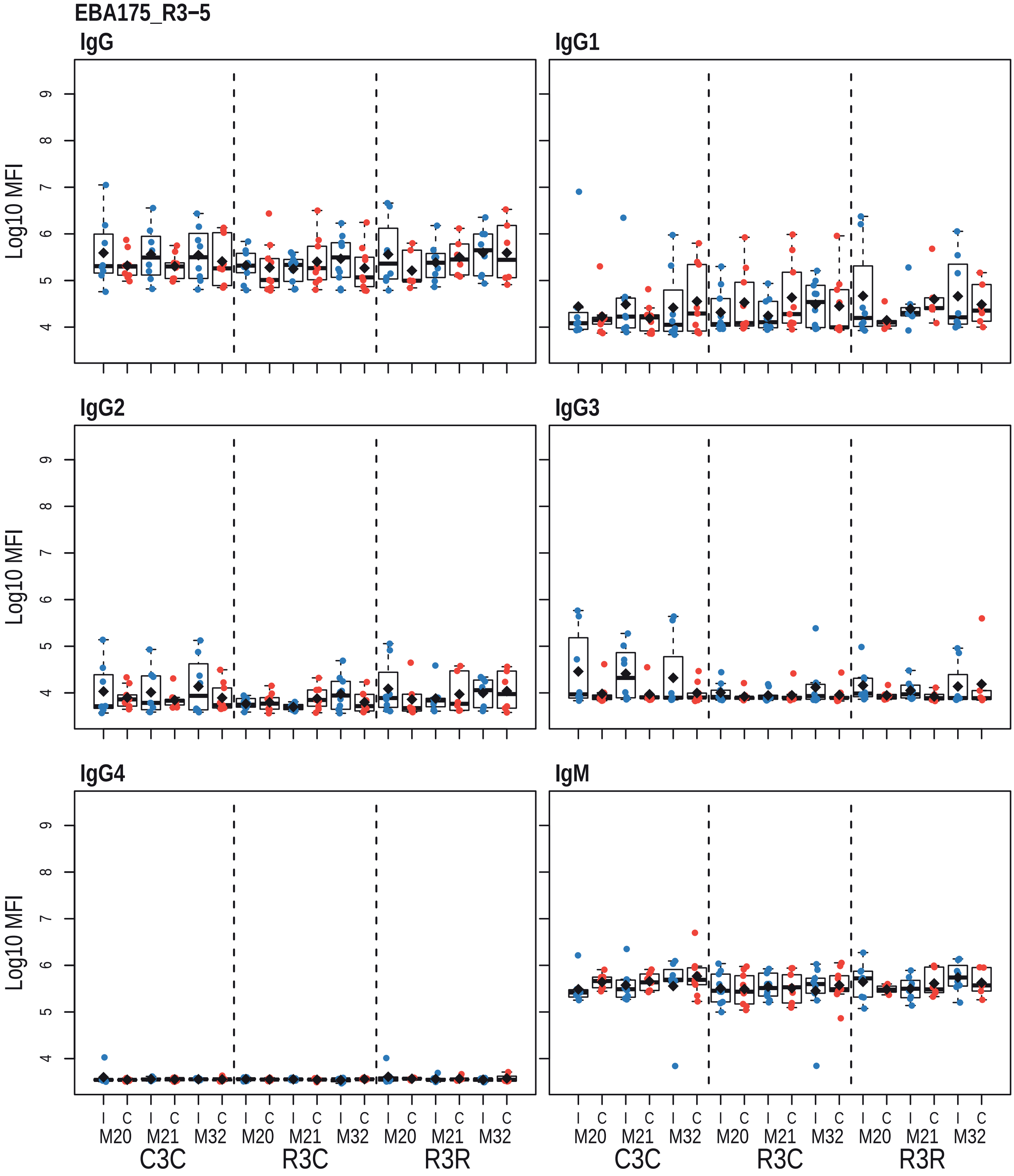

Supplementary Figure 18. RTS,S/AS01E booster and long-term immunogenicity stratified by age against blood stage antigens: IgG, IgG1-4 subclasses and IgM for EBA175 R3-5 at month (M) 20, M21 and M32 for RTS,S/AS01 vaccinees with (R3R) and without (R3C) booster, and comparator (C3C). Stratified analysis by age, children (C) age 5-17 months (red) and infants (I) age 6-12 weeks (blue) at the time of first vaccination. Boxplots with medians, interquartile ranges (IQR), upper whisker as the smallest between maximum x value and Q3 + 1.5 * IQR, lower whisker as the largest between minimum x value and Q1 – 1.5 * IQR, and log10(geometric mean(MFI)) (diamond). Non-parametric tests were used to compare the booster response (M20 vs M21), the long-term immunogenicity (M21 vs M32), and the effect of age (children vs infants). P-values were not significant after adjustment for multiple testing. The y axis is in logarithm 10 scale. R3R: three doses of RTS,S/AS01E and a RTS,S/AS01E booster. R3C: three doses of RTS,S/AS01E and a comparator booster. C3C: three doses of a comparator vaccine and a comparator booster.

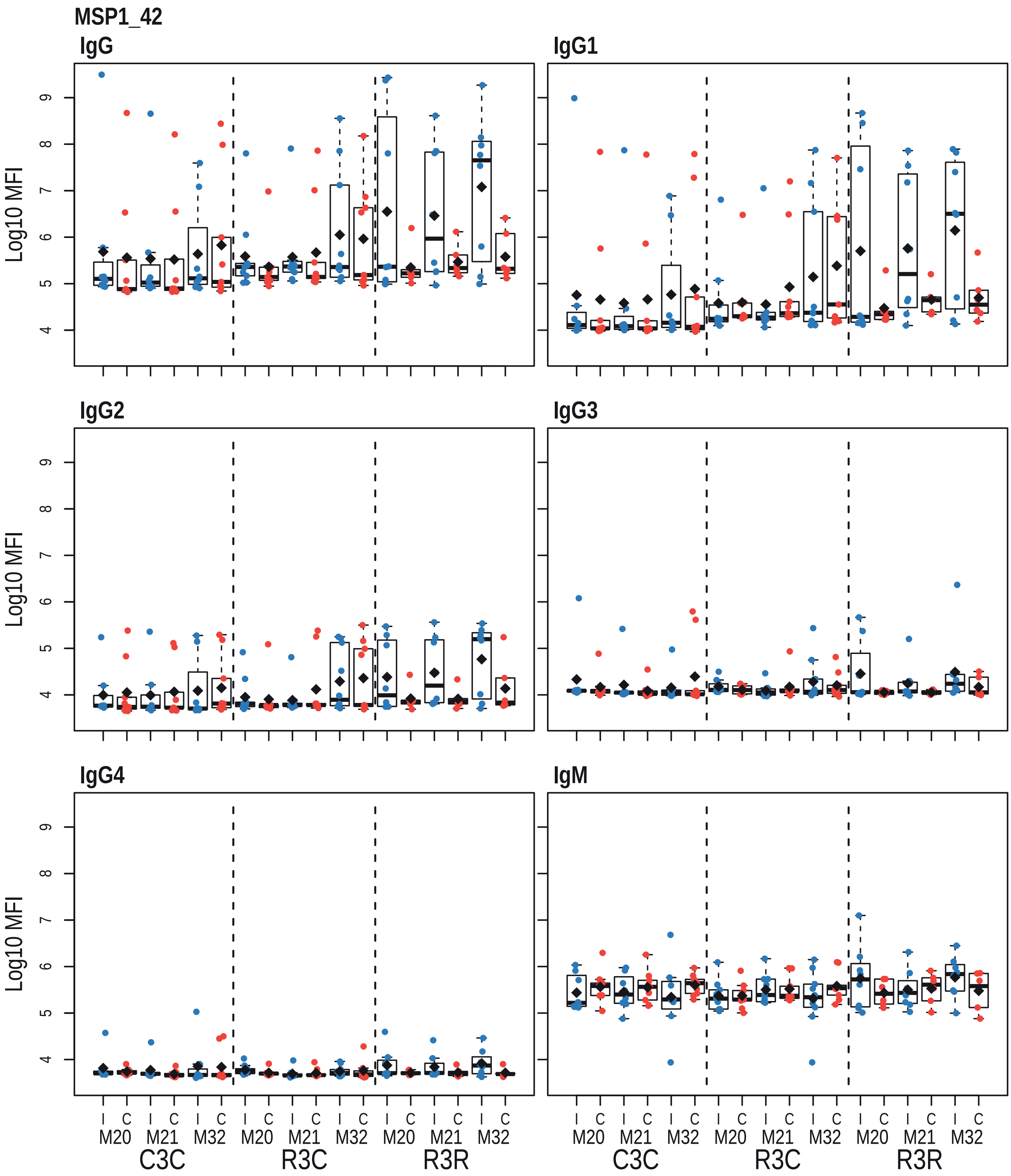

Supplementary Figure 19. RTS,S/AS01E booster and long-term immunogenicity stratified by age against blood stage antigens: IgG, IgG1-4 subclasses and IgM for MSP1_{42} at month (M) 20, M21 and M32 for RTS,S/AS01 vaccinees with (R3R) and without (R3C) booster, and comparator (C3C). Stratified analysis by age, children (C) age 5-17 months (red) and infants (I) age 6-12 weeks (blue) at the time of first vaccination. Boxplots with medians, interquartile ranges (IQR), upper whisker as the smallest between maximum x value and Q3 + 1.5 * IQR, lower whisker as the largest between minimum x value and Q1 – 1.5 * IQR, and log10(geometric mean(MFI)) (diamond). Non-parametric tests were used to compare the booster response (M20 vs M21), the long-term immunogenicity (M21 vs M32), and the effect of age (children vs infants). P-values were not significant after adjustment for multiple testing. The y axis is in logarithm 10 scale. R3R: three doses of RTS,S/AS01E and a RTS,S/AS01E booster. R3C: three doses of RTS,S/AS01E and a comparator booster. C3C: three doses of a comparator vaccine and a comparator booster.

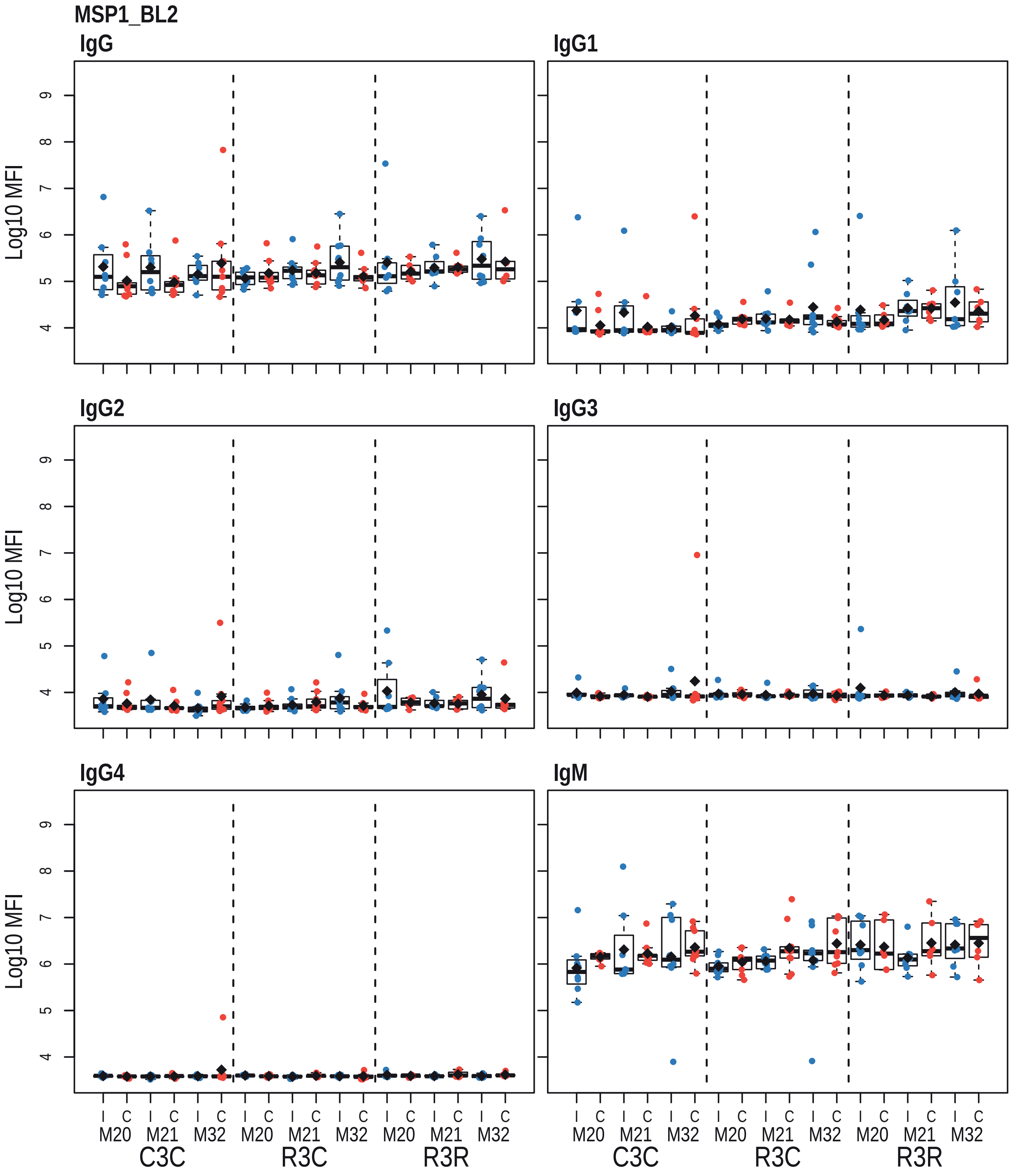

Supplementary Figure 20. RTS,S/AS01E booster and long-term immunogenicity stratified by age against blood stage antigens: IgG, IgG1-4 subclasses and IgM for MSP1 Block2 at month (M) 20, M21 and M32 for RTS,S/AS01 vaccinees with (R3R) and without (R3C) booster, and comparator (C3C). Stratified analysis by age, children (C) age 5-17 months (red) and infants (I) age 6-12 weeks (blue) at the time of first vaccination. Boxplots with medians, interquartile ranges (IQR), upper whisker as the smallest between maximum x value and Q3 + 1.5 * IQR, lower whisker as the largest between minimum x value and Q1 – 1.5 * IQR, and log10(geometric mean(MFI)) (diamond). Non-parametric tests were used to compare the booster response (M20 vs M21), the long-term immunogenicity (M21 vs M32), and the effect of age (children vs infants). P-values were not significant after adjustment for multiple testing. The y axis is in logarithm 10 scale. R3R: three doses of RTS,S/AS01E and a RTS,S/AS01E booster. R3C: three doses of RTS,S/AS01E and a comparator booster. C3C: three doses of a comparator vaccine and a comparator booster.

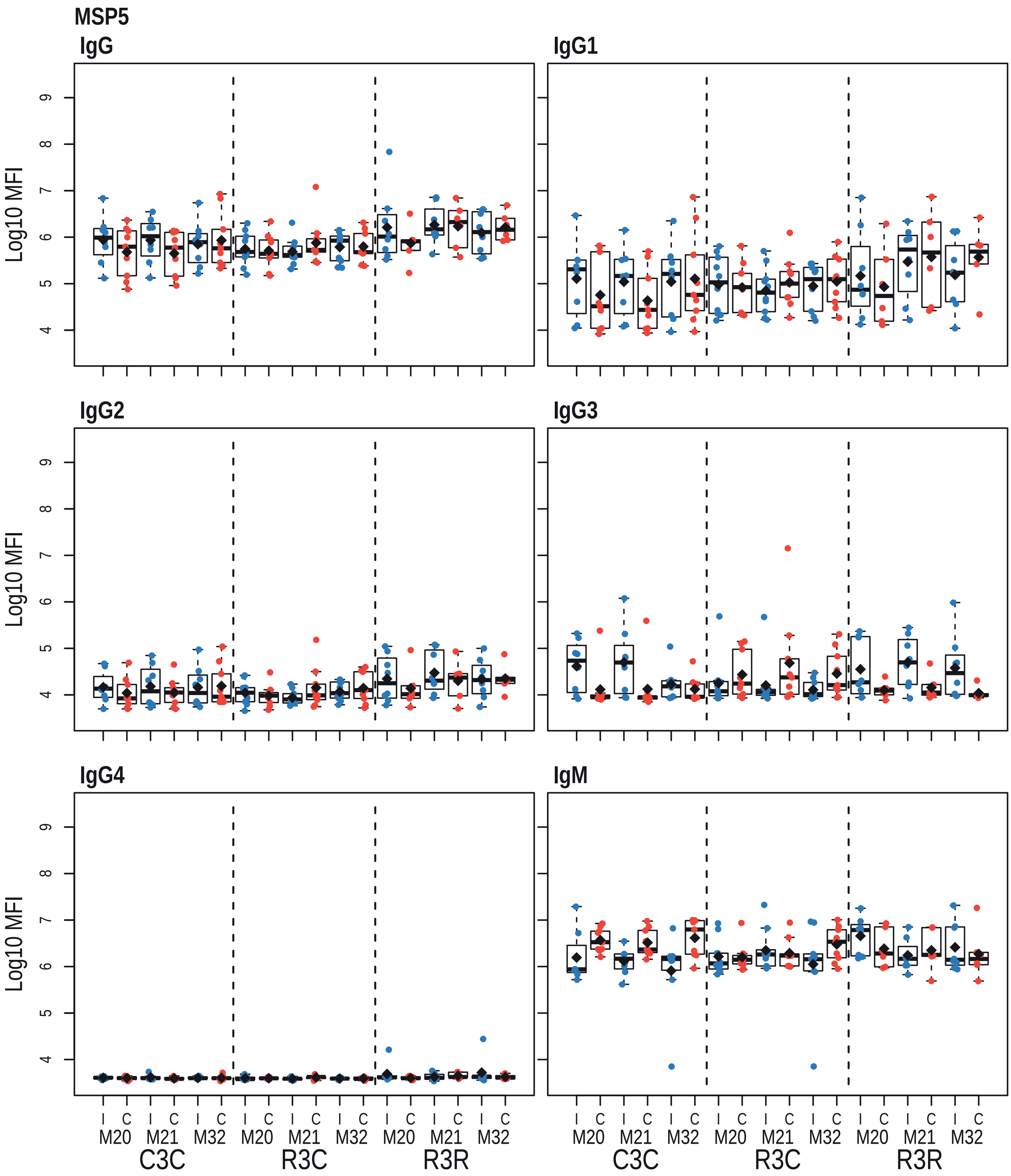

Supplementary Figure 21. RTS,S/AS01E booster and long-term immunogenicity stratified by age against blood stage antigens: IgG, IgG1-4 subclasses and IgM for MSP5 at month (M) 20, M21 and M32 for RTS,S/AS01 vaccinees with (R3R) and without (R3C) booster, and comparator (C3C). Stratified analysis by age, children (C) age 5-17 months (red) and infants (I) age 6-12 weeks (blue) at the time of first vaccination. Boxplots with medians, interquartile ranges (IQR), upper whisker as the smallest between maximum x value and Q3 + 1.5 * IQR, lower whisker as the largest between minimum x value and Q1 – 1.5 * IQR, and log10(geometric mean(MFI)) (diamond). Non-parametric tests were used to compare the booster response (M20 vs M21), the long-term immunogenicity (M21 vs M32), and the effect of age (children vs infants). P-values were not significant after adjustment for multiple testing. The y axis is in logarithm 10 scale. R3R: three doses of RTS,S/AS01E and a RTS,S/AS01E booster. R3C: three doses of RTS,S/AS01E and a comparator booster. C3C: three doses of a comparator vaccine and a comparator booster.

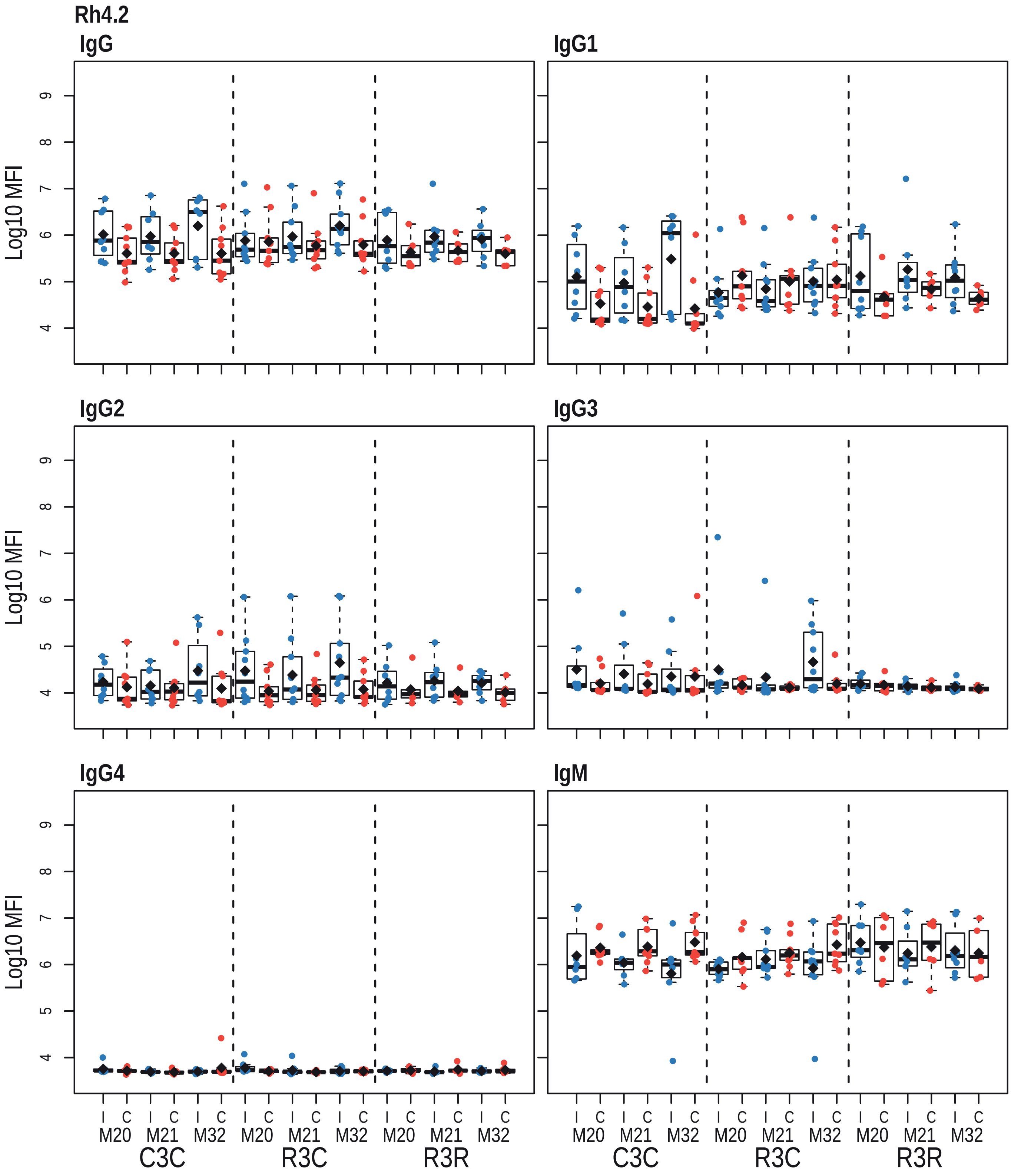

Supplementary Figure 22. RTS,S/AS01E booster and long-term immunogenicity stratified by age against blood stage antigens: IgG, IgG1-4 subclasses and IgM for Rh4.2 at month (M) 20, M21 and M32 for RTS,S/AS01 vaccinees with (R3R) and without (R3C) booster, and comparator (C3C). Stratified analysis by age, children (C) age 5-17 months (red) and infants (I) age 6-12 weeks (blue) at the time of first vaccination. Boxplots with medians, interquartile ranges (IQR), upper whisker as the smallest between maximum x value and Q3 + 1.5 * IQR, lower whisker as the largest between minimum x value and Q1 – 1.5 * IQR, and log10(geometric mean(MFI)) (diamond). Non-parametric tests were used to compare the booster response (M20 vs M21), the long-term immunogenicity (M21 vs M32), and the effect of age (children vs infants). P-values were not significant after adjustment for multiple testing. The y axis is in logarithm 10 scale. R3R: three doses of RTS,S/AS01E and a RTS,S/AS01E booster. R3C: three doses of RTS,S/AS01E and a comparator booster. C3C: three doses of a comparator vaccine and a comparator booster.

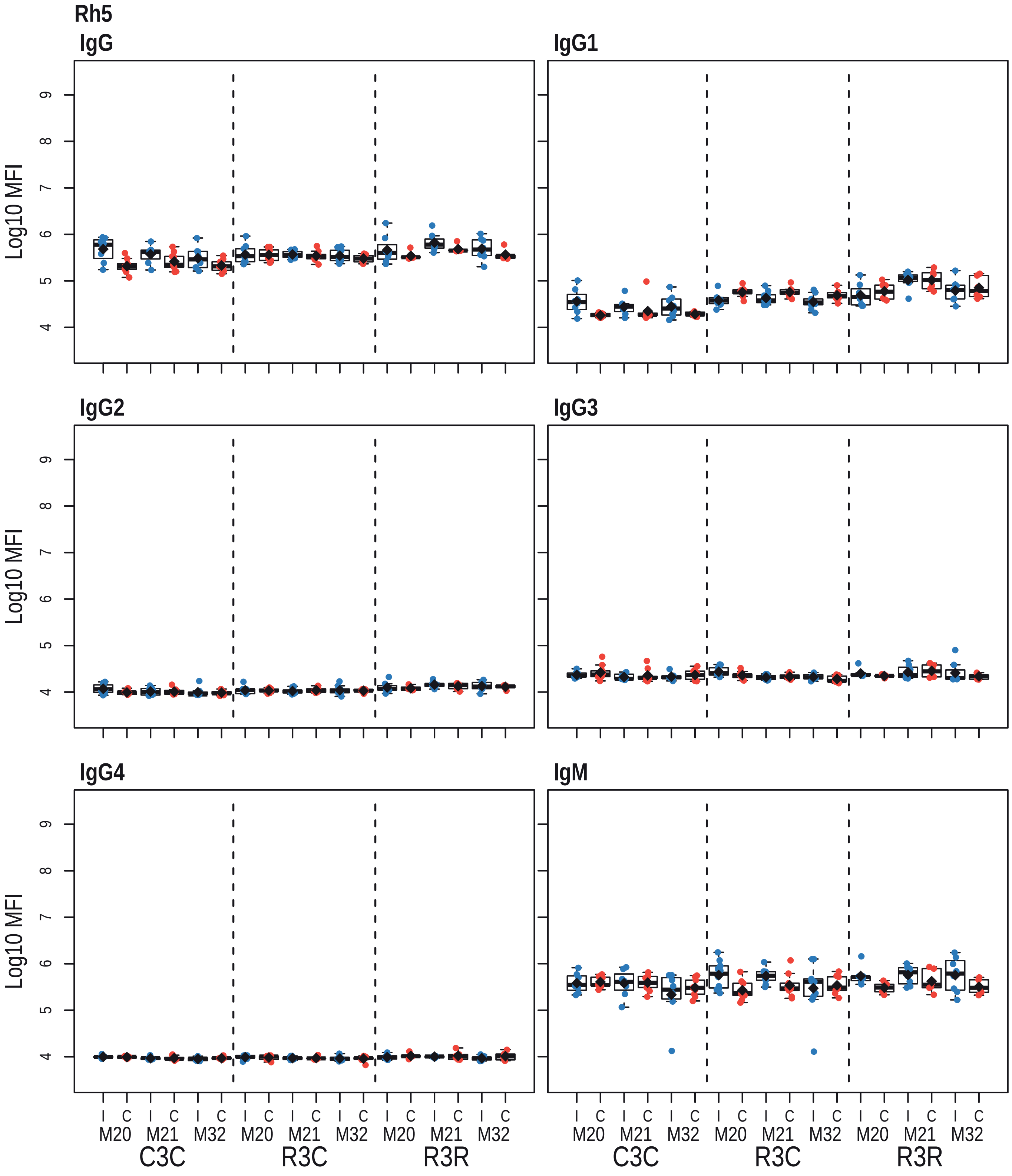

Supplementary Figure 23. RTS,S/AS01E booster and long-term immunogenicity stratified by age against blood stage antigens: IgG, IgG1-4 subclasses and IgM for Rh5 at month (M) 20, M21 and M32 for RTS,S/AS01 vaccinees with (R3R) and without (R3C) booster, and comparator (C3C). Stratified analysis by age, children (C) age 5-17 months (red) and infants (I) age 6-12 weeks (blue) at the time of first vaccination. Boxplots with medians, interquartile ranges (IQR), upper whisker as the smallest between maximum x value and Q3 + 1.5 * IQR, lower whisker as the largest between minimum x value and Q1 – 1.5 * IQR, and log10(geometric mean(MFI)) (diamond). Non-parametric tests were used to compare the booster response (M20 vs M21), the long-term immunogenicity (M21 vs M32), and the effect of age (children vs infants). P-values were not significant after adjustment for multiple testing. The y axis is in logarithm 10 scale. R3R: three doses of RTS,S/AS01E and a RTS,S/AS01E booster. R3C: three doses of RTS,S/AS01E and a comparator booster. C3C: three doses of a comparator vaccine and a comparator booster.

Supplementary Table 5. RTS,S/AS01E booster and long-term immunogenicity against Plasmodium falciparum blood stage antigens stratified by age: Comparisons of antibody levels between timepoints and age groups for total IgG, IgG1-4 subclasses and IgM. RTS,S/AS01 vaccinees with (R3R) and without (R3C) booster, and comparator (C3C). Stratified analysis by age, children 5-17 months and infants 6-12 weeks at the time of first vaccination. Non-parametric tests were used to compare the booster response (M20 vs M21), the long-term immunogenicity (M21 vs M32), and the effect of age (children vs infants). Adjusted p values are shown parenthesis. P-values <0.05 before adjustment are highlighted in yellow.

		Comparison between timepoints						Age comparison					
		M20vsM21		M21vsM32				Children vs Infants					
		R3R		R3R		R3C		R3C			R3R		
Isotype	Antigen	Infants	Children	Infants	Children	Infants	Children	M20	M21	M32	M20	M21	M32
IgG	EBA140	1	1	1	1	1	1	1	1	1	1	1	1
IgG	EBA175_R3−5	1	1	1	1	1	1	1	1	1	1	1	1
IgG	MSP1_42	1	1	1	1	1	1	1	1	1	1	1	1
IgG	MSP1_BL2	1	1	1	1	1	1	1	1	1	1	1	1
IgG	MSP5	1	1	1	1	1	1	1	1	1	1	1	1
IgG	Rh4.2	1	1	1	1	0.08	1	1	1	1	1	1	1
IgG	Rh5	0.96	1	1	1	1	1	1	1	1	1	1	1
IgG1	EBA140	0.95	0.88	0.95	0.95	0.95	0.95	0.99	0.99	0.99	0.99	0.92	1
IgG1	EBA175_R3−5	1	0.65	0.95	0.91	0.91	0.74	0.99	0.99	0.99	0.99	0.99	0.99
IgG1	MSP1_42	0.95	0.68	0.95	0.91	0.95	1	0.92	0.92	0.99	1	0.99	0.98
IgG1	MSP1_BL2	0.78	0.65	1	0.91	0.88	0.74	0.92	1	0.99	0.99	1	0.99
IgG1	MSP5	0.91	0.68	0.84	1	0.95	0.95	1	0.99	0.99	0.99	0.99	0.99
IgG1	Rh4.2	0.91	0.88	0.95	0.74	0.68	1	0.99	0.99	1	0.99	0.99	0.92
IgG1	Rh5	0.65	0.65	0.65	0.65	0.78	0.65	0.92	0.92	0.92	0.99	1	0.99
IgG2	EBA140	0.91	0.91	1	1	0.95	1	0.99	0.99	0.99	0.99	0.99	0.99
IgG2	EBA175_R3−5	0.84	1	0.91	0.95	0.65	0.91	1	0.92	0.99	0.99	0.99	0.99
IgG2	MSP1_42	0.95	0.95	0.95	0.91	0.74	0.95	0.99	0.99	0.99	0.99	0.92	0.98
IgG2	MSP1_BL2	0.95	0.95	0.88	1	0.88	0.74	0.99	0.99	0.99	1	1	0.99
IgG2	MSP5	0.91	0.91	0.88	1	0.74	0.95	0.99	0.99	0.99	0.99	0.99	1
IgG2	Rh4.2	0.84	0.95	1	0.95	0.65	1	0.94	0.99	0.92	0.99	0.99	0.92
IgG2	Rh5	0.73	0.74	0.91	0.91	0.95	0.95	0.99	0.99	1	0.99	0.99	0.99
IgG3	EBA140	1	0.74	0.74	1	0.95	0.74	0.92	0.92	0.99	1	0.99	0.99
IgG3	EBA175_R3−5	0.95	0.84	1	0.95	0.95	0.91	0.99	0.99	0.99	0.98	0.92	1
IgG3	MSP1_42	0.95	0.95	0.88	0.91	0.78	0.95	0.99	0.99	0.99	0.99	0.99	0.99
IgG3	MSP1_BL2	0.91	0.91	0.95	0.95	0.95	0.91	1	0.92	0.99	0.99	0.99	0.99
IgG3	MSP5	0.95	0.91	0.91	0.74	0.95	0.88	0.99	0.99	0.92	0.99	0.92	0.92
IgG3	Rh4.2	0.91	0.84	0.95	0.95	0.74	0.95	0.99	0.99	0.92	0.99	0.99	0.99
IgG3	Rh5	0.95	0.74	0.84	0.68	0.95	0.65	0.92	0.99	0.99	0.94	0.99	1
IgG4	EBA140	0.95	0.95	0.74	0.95	0.95	0.65	0.99	0.99	0.99	0.99	1	1
IgG4	EBA175_R3−5	0.88	0.95	0.95	0.95	0.74	0.95	0.99	0.99	0.99	0.99	0.99	0.99
IgG4	MSP1_42	0.84	0.95	0.95	0.95	0.74	0.95	0.92	0.99	0.99	0.99	0.99	0.99
IgG4	MSP1_BL2	0.91	0.88	1	1	0.78	0.95	0.92	0.99	0.99	0.99	0.99	0.99
IgG4	MSP5	0.95	0.74	0.91	0.68	0.95	0.74	0.99	0.92	1	0.99	0.99	0.99
IgG4	Rh4.2	0.84	0.95	0.78	0.95	0.95	0.74	0.92	0.99	1	1	0.92	1
IgG4	Rh5	0.95	1	0.73	0.95	0.95	0.95	0.99	1	0.99	0.99	1	0.99
IgM	EBA140	1	1	1	1	1	1	1	1	1	0.19	1	1
IgM	EBA175_R3−5	1	1	1	1	1	1	1	1	1	1	1	1
IgM	MSP1_42	1	1	1	1	1	1	1	1	1	1	1	1
IgM	MSP1_BL2	1	1	1	1	1	1	1	1	1	1	1	1
IgM	MSP5	0.33	1	1	1	1	1	1	1	1	1	1	1
IgM	Rh4.2	0.33	1	1	1	1	1	1	1	1	1	1	1
IgM	Rh5	1	1	1	1	1	1	0.53	1	1	0.11	1	1

Supplementary Table 6. Immunogenicity to Plasmodium falciparum blood stage antigens stratified by previous clinical malaria (left) and by clinical malaria after booster dose (right): Non-parametric test p-values for comparison of levels with or without clinical malaria (M vs NM) for IgG, IgG1-4 subclasses and IgM at month (M)20, 21 and 32 for RTS,S/AS01 vaccinees with (R3R) and without (R3C) booster, and comparator (C3C). P-values <0.05 are highlighted in yellow. P-values were adjusted for multiple comparisons, but none was significant and are not shown.

		Prebooster cases: Malaria vs No Malaria								
		C3C			R3C			R3R		
Isotype	Antigen	M20	M21	M32	M20	M21	M32	M20	M21	M32
IgG	EBA140	0.30	0.41	0.78	0.95	0.84	0.49	0.61	0.84	0.80
IgG	EBA175_R3-5	0.1	0.20	0.70	0.89	1	0.65	0.08	0.80	0.08
IgG	MSP1_42	<0.001	<0.001	0.006	0.19	0.19	0.65	0.03	0.11	0.004
IgG	MSP1_BL2	0.48	0.41	0.48	1	0.84	0.11	0.08	0.90	0.24
IgG	MSP5	0.01	0.03	0.70	0.65	0.49	0.49	0.61	0.61	0.61
IgG	Rh4.2	0.30	0.41	0.48	0.57	0.95	0.75	0.44	0.24	0.52
IgG	Rh5	0.48	0.13	0.61	0.42	0.57	0.49	0.61	0.70	0.19
IgG1	EBA140	0.34	0.23	0.62	0.49	0.65	0.23	0.15	1	0.52
IgG1	EBA175_R3-5	0.16	0.41	0.48	0.59	0.57	0.55	0.08	0.80	0.02
IgG1	MSP1_42	0.004	<0.001	0.006	0.84	0.95	0.95	0.004	0.11	0.004
IgG1	MSP1_BL2	0.33	0.39	0.70	0.1	0.55	0.46	0.08	0.80	0.02
IgG1	MSP5	0.08	0.30	0.87	0.29	0.23	0.65	0.19	0.70	0.44
IgG1	Rh4.2	0.96	0.62	0.70	0.01	0.19	0.23	0.52	0.30	0.19
IgG1	Rh5	0.73	0.09	0.70	0.01	0.07	0.29	0.36	0.90	0.42
IgG2	EBA140	0.16	0.19	1	1	0.29	0.46	1	1	0.80
IgG2	EBA175_R3-5	0.13	0.34	0.53	1	0.35	0.84	0.11	0.44	0.36
IgG2	MSP1_42	0.004	0.004	0.01	0.07	0.84	0.42	0.042	0.007	0.06
IgG2	MSP1_BL2	0.17	1	0.28	0.84	0.75	0.13	0.24	0.61	0.11
IgG2	MSP5	0.004	0.006	0.57	0.51	0.51	0.65	0.44	0.61	0.90
IgG2	Rh4.2	0.26	0.30	0.23	0.65	0.49	1	0.52	0.30	0.61
IgG2	Rh5	0.65	0.03	0.53	0.69	0.07	0.84	1	0.84	0.80
IgG3	EBA140	0.73	0.78	0.23	0.32	0.55	1	0.44	0.50	0.84
IgG3	EBA175_R3-5	0.16	0.69	0.73	0.1	0.13	0.95	0.36	0.08	1
IgG3	MSP1_42	0.14	0.03	0.36	0.03	0.07	0.95	0.01	0.08	0.02
IgG3	MSP1_BL2	0.73	0.86	0.14	0.21	0.16	0.04	0.44	0.80	0.46
IgG3	MSP5	0.13	0.21	0.87	0.65	0.95	0.29	0.80	0.90	0.84
IgG3	Rh4.2	0.16	0.13	0.62	0.23	0.1	1	0.19	0.44	0.42
IgG3	Rh5	0.69	0.73	0.87	0.35	0.054	0.32	0.39	0.61	0.79
IgG4	EBA140	0.26	0.78	0.73	0.59	0.55	0.64	0.95	0.59	0.79
IgG4	EBA175_R3-5	0.65	0.48	0.03	0.32	0.79	0.69	0.03	1	1
IgG4	MSP1_42	0.054	0.01	0.02	0.07	0.054	0.55	0.11	0.23	0.02
IgG4	MSP1_BL2	0.36	0.28	0.73	0.18	0.79	0.16	0.79	0.35	0.64
IgG4	MSP5	0.08	0.39	0.13	0.26	0.32	0.46	0.89	0.19	0.69
IgG4	Rh4.2	0.91	0.19	0.57	0.59	0.32	0.55	0.46	1	0.50
IgG4	Rh5	0.31	0.65	0.87	0.64	0.35	0.23	0.70	0.36	0.39
IgM	EBA140	0.62	0.78	0.55	0.42	0.42	0.49	0.15	0.90	0.36
IgM	EBA175_R3-5	0.62	0.1	0.70	1	0.95	0.14	0.44	1	0.90
IgM	MSP1_42	0.16	0.25	0.62	0.19	1	0.23	0.02	0.61	0.02
IgM	MSP1_BL2	0.62	0.87	0.03	0.75	0.95	0.84	0.30	0.61	0.70
IgM	MSP5	0.16	0.55	0.96	0.42	0.29	0.42	0.52	0.52	0.70
IgM	Rh4.2	0.35	0.35	0.30	0.42	0.35	0.95	0.80	0.44	1
IgM	Rh5	0.20	0.16	0.55	0.07	0.14	0.57	0.61	0.19	0.24

		Postbooster cases: Malaria vs No Malaria								
		C3C			R3C			R3R		
Isotype	Antigen	M20	M21	M32	M20	M21	M32	M20	M21	M32
IgG	EBA140	0.68	0.43	0.59	0.81	0.41	0.81	0.73	0.1	0.02
IgG	EBA175_R3-5	0.07	0.12	0.01	0.15	0.26	0.009	0.95	0.14	0.37
IgG	MSP1_42	0.047	0.047	0.03	0.22	0.89	0.08	0.73	0.37	0.054
IgG	MSP1_BL2	0.68	0.86	0.047	0.049	0.53	0.60	0.37	0.19	0.30
IgG	MSP5	0.16	0.51	0.16	0.41	0.81	0.08	0.64	0.08	0.64
IgG	Rh4.2	0.95	0.68	0.12	0.96	0.96	0.74	0.64	0.73	0.45
IgG	Rh5	0.24	0.16	0.15	0.53	0.41	0.1	0.37	0.30	0.73
IgG1	EBA140	1	0.90	0.59	0.74	0.96	0.36	0.64	0.64	0.30
IgG1	EBA175_R3-5	0.09	0.43	0.003	0.65	0.74	0.01	0.48	0.054	0.30
IgG1	MSP1_42	0.07	0.047	0.09	0.03	0.31	0.15	0.84	0.30	0.054
IgG1	MSP1_BL2	0.66	0.57	0.16	0.23	0.45	0.96	1	0.30	0.19
IgG1	MSP5	0.24	0.68	0.20	0.74	0.26	0.009	0.84	0.24	0.73
IgG1	Rh4.2	0.43	0.68	0.68	0.53	0.89	0.60	0.73	0.84	0.84
IgG1	Rh5	0.80	0.34	0.59	0.89	0.89	0.31	0.73	1	0.89
IgG2	EBA140	0.43	0.75	0.77	0.73	0.41	0.40	0.54	0.26	0.01
IgG2	EBA175_R3-5	0.07	0.15	0.03	0.25	0.31	0.01	0.95	0.04	0.04
IgG2	MSP1_42	0.04	0.051	0.07	0.23	0.84	0.15	0.95	0.19	0.002
IgG2	MSP1_BL2	0.21	0.75	0.03	0.62	0.96	0.84	0.11	0.04	0.11
IgG2	MSP5	0.17	0.30	0.23	0.58	0.34	0.06	0.73	0.04	0.24
IgG2	Rh4.2	0.90	0.24	0.49	0.41	0.60	0.31	0.73	0.30	0.04
IgG2	Rh5	0.57	0.07	0.49	0.55	0.48	0.73	0.72	0.29	0.008
IgG3	EBA140	0.53	0.75	0.07	0.21	0.76	0.69	0.84	0.44	0.006
IgG3	EBA175_R3-5	0.12	0.34	0.02	0.02	0.45	0.02	0.54	0.62	0.72
IgG3	MSP1_42	0.41	0.16	0.044	0.34	0.66	0.12	0.84	0.48	0.054
IgG3	MSP1_BL2	0.85	0.85	0.051	1	0.76	1	0.64	0.84	0.29
IgG3	MSP5	0.95	1	0.41	0.41	0.80	0.1	1	0.30	0.72
IgG3	Rh4.2	0.047	0.03	0.01	1	0.88	0.12	0.64	0.95	0.44
IgG3	Rh5	0.28	0.61	0.01	0.47	0.80	0.29	0.04	0.04	0.14
IgG4	EBA140	0.75	0.01	0.66	0.32	0.04	0.03	0.48	0.16	0.02
IgG4	EBA175_R3-5	0.25	0.24	0.06	0.34	0.84	0.1	0.67	0.32	0.52
IgG4	MSP1_42	0.71	0.31	0.16	0.29	0.45	0.051	0.78	0.12	0.08
IgG4	MSP1_BL2	0.08	0.41	1	0.31	0.13	0.12	0.57	0.57	0.26
IgG4	MSP5	0.53	0.41	0.07	0.19	0.19	0.29	0.72	0.054	0.12
IgG4	Rh4.2	0.70	0.66	0.31	0.92	0.057	0.29	0.62	0.67	1
IgG4	Rh5	0.07	0.75	0.43	0.58	0.51	0.41	0.37	0.73	0.72
IgM	EBA140	0.86	0.16	0.43	0.1	1	0.31	0.11	0.19	0.84
IgM	EBA175_R3-5	0.95	0.36	0.36	0.74	0.96	0.66	0.054	0.04	0.054
IgM	MSP1_42	0.95	1	0.95	0.74	0.18	0.1	0.45	0.37	0.64
IgM	MSP1_BL2	0.95	0.51	0.09	0.66	0.53	0.47	0.24	0.24	0.95
IgM	MSP5	1	0.86	0.43	0.53	0.53	0.96	0.30	0.64	0.054
IgM	Rh4.2	0.95	0.77	0.95	0.31	0.18	0.60	0.14	0.19	0.19
IgM	Rh5	0.36	0.30	0.95	0.31	0.22	0.15	0.08	0.24	0.02

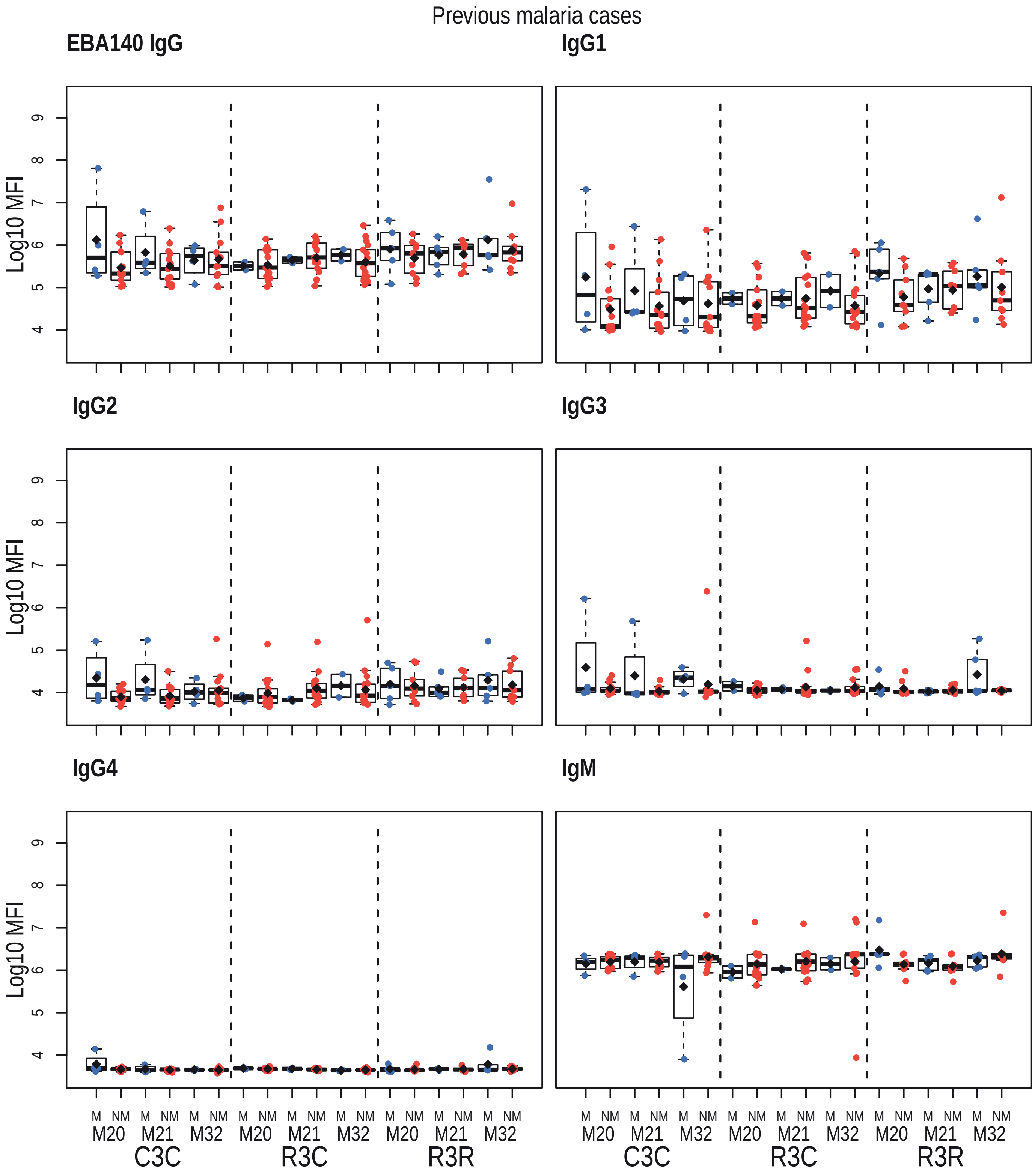

Supplementary Figure 24. Immunogenicity stratified by previous clinical malaria against blood stage antigens: Total IgG, IgG1-4 subclasses and IgM for EBA140 at month (M) 20, 21 and 32 for RTS,S/AS01 vaccinees with (R3R) and without (R3C) booster, and comparator (C3C). Stratified analysis by malaria cases before M20, subjects who presented with clinical malaria (M=blue) and subjects without clinical malaria (NM=red). Boxplots with medians, interquartile ranges (IQR), upper whisker as the smallest between maximum x value and Q3 + 1.5 * IQR, lower whisker as the largest between minimum x value and Q1 – 1.5 * IQR, and log10(geometric mean(MFI)) (diamond). Non-parametric tests were used to compare levels with or without clinical malaria (M vs NM). P-values were adjusted for multiple comparisons, but none was significant. Only p-values <0.05 before adjustment are shown. The y axis is in logarithm 10 scale. R3R: three doses of RTS,S/AS01E and a RTS,S/AS01E booster. R3C: three doses of RTS,S/AS01E and a comparator booster. C3C: three doses of a comparator vaccine and a comparator booster.

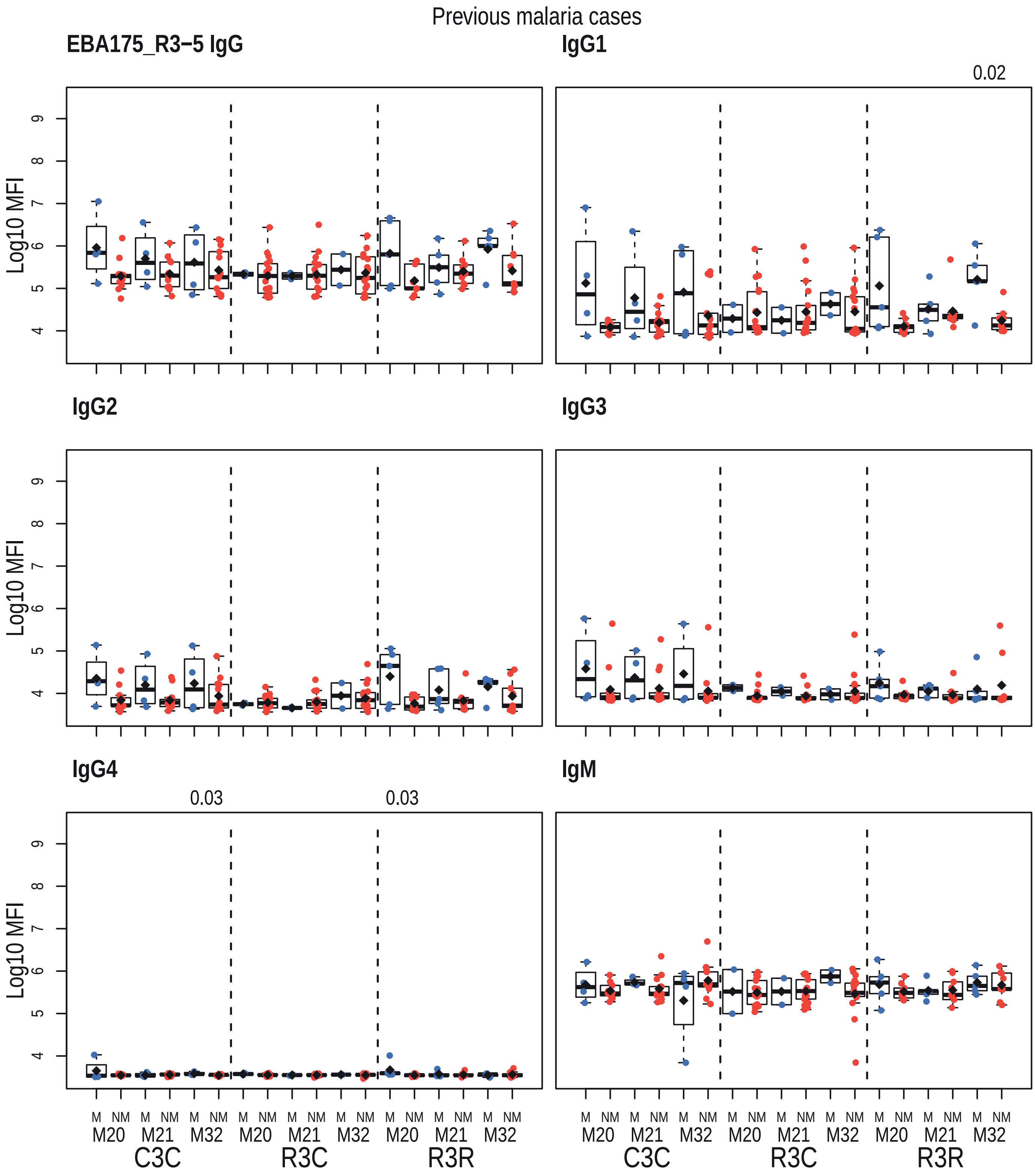

Supplementary Figure 25. Immunogenicity stratified by previous clinical malaria against blood stage antigens: Total IgG, IgG1-4 subclasses and IgM for EBA175 R3-5 at month (M) 20, 21 and 32 for RTS,S/AS01 vaccinees with (R3R) and without (R3C) booster, and comparator (C3C). Stratified analysis by malaria cases before M20, subjects who presented with clinical malaria (M=blue) and subjects without clinical malaria (NM=red). Boxplots with medians, interquartile ranges (IQR), upper whisker as the smallest between maximum x value and Q3 + 1.5 * IQR, lower whisker as the largest between minimum x value and Q1 – 1.5 * IQR, and log10(geometric mean(MFI)) (diamond). Non-parametric tests were used to compare levels with or without clinical malaria (M vs NM). P-values were adjusted for multiple comparisons, but none was significant. Only p-values <0.05 before adjustment are shown. The y axis is in logarithm 10 scale. R3R: three doses of RTS,S/AS01E and a RTS,S/AS01E booster. R3C: three doses of RTS,S/AS01E and a comparator booster. C3C: three doses of a comparator vaccine and a comparator booster.

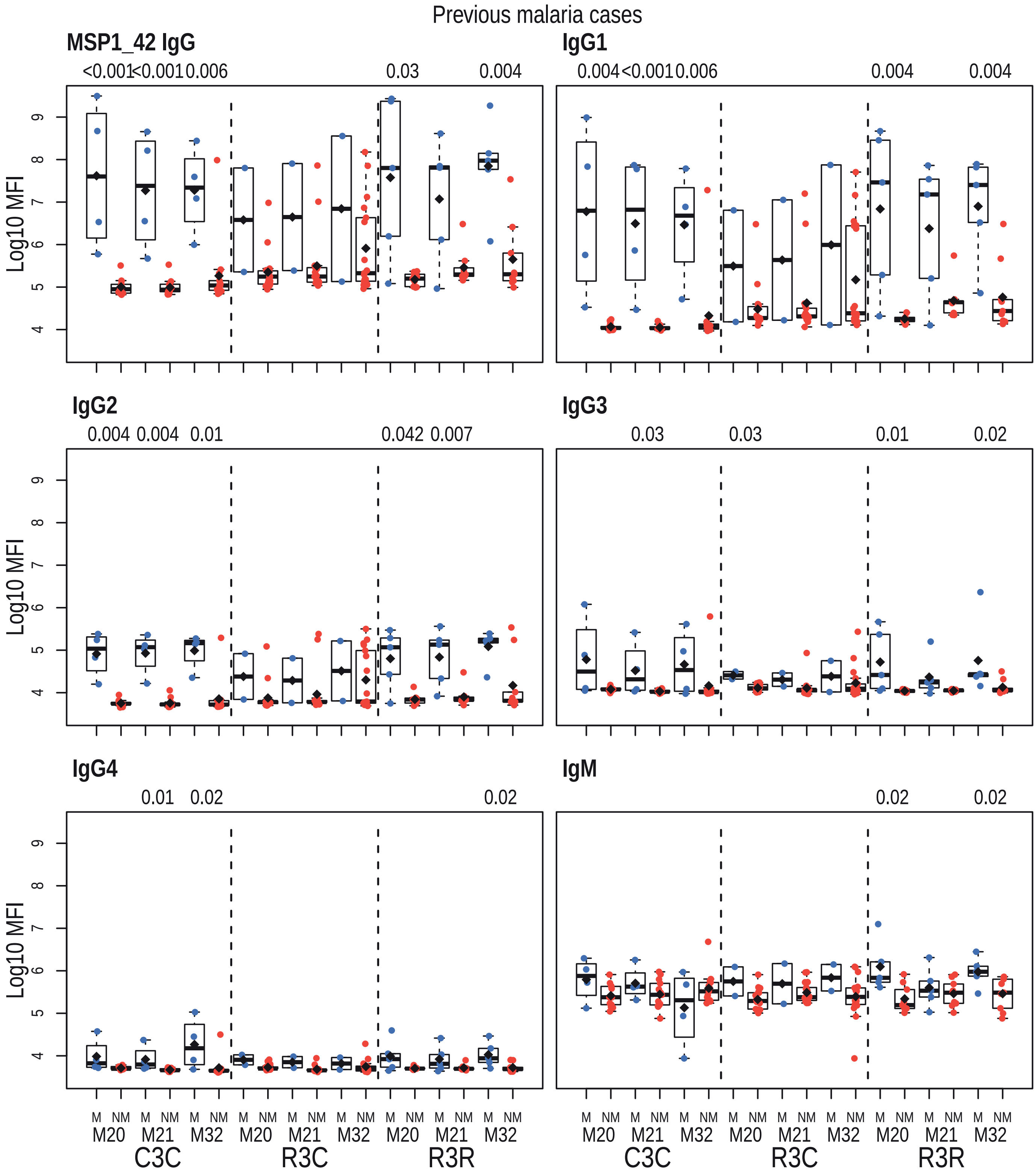

Supplementary Figure 26. Immunogenicity stratified by previous clinical malaria against blood stage antigens: Total IgG, IgG1-4 subclasses and IgM for $MSP1_{42}$ at month (M) 20, 21 and 32 for RTS,S/AS01 vaccinees with (R3R) and without (R3C) booster, and comparator (C3C). Stratified analysis by malaria cases before M20, subjects who presented with clinical malaria (M=blue) and subjects without clinical malaria (NM=red). Boxplots with medians, interquartile ranges (IQR), upper whisker as the smallest between maximum x value and Q3 + 1.5 * IQR, lower whisker as the largest between minimum x value and Q1 – 1.5 * IQR, and log10(geometric mean(MFI)) (diamond). Non-parametric tests were used to compare levels with or without clinical malaria (M vs NM). P-values were adjusted for multiple comparisons, but none was significant. Only p-values <0.05 before adjustment are shown. The y axis is in logarithm 10 scale. R3R: three doses of RTS,S/AS01E and a RTS,S/AS01E booster. R3C: three doses of RTS,S/AS01E and a comparator booster. C3C: three doses of a comparator vaccine and a comparator booster.

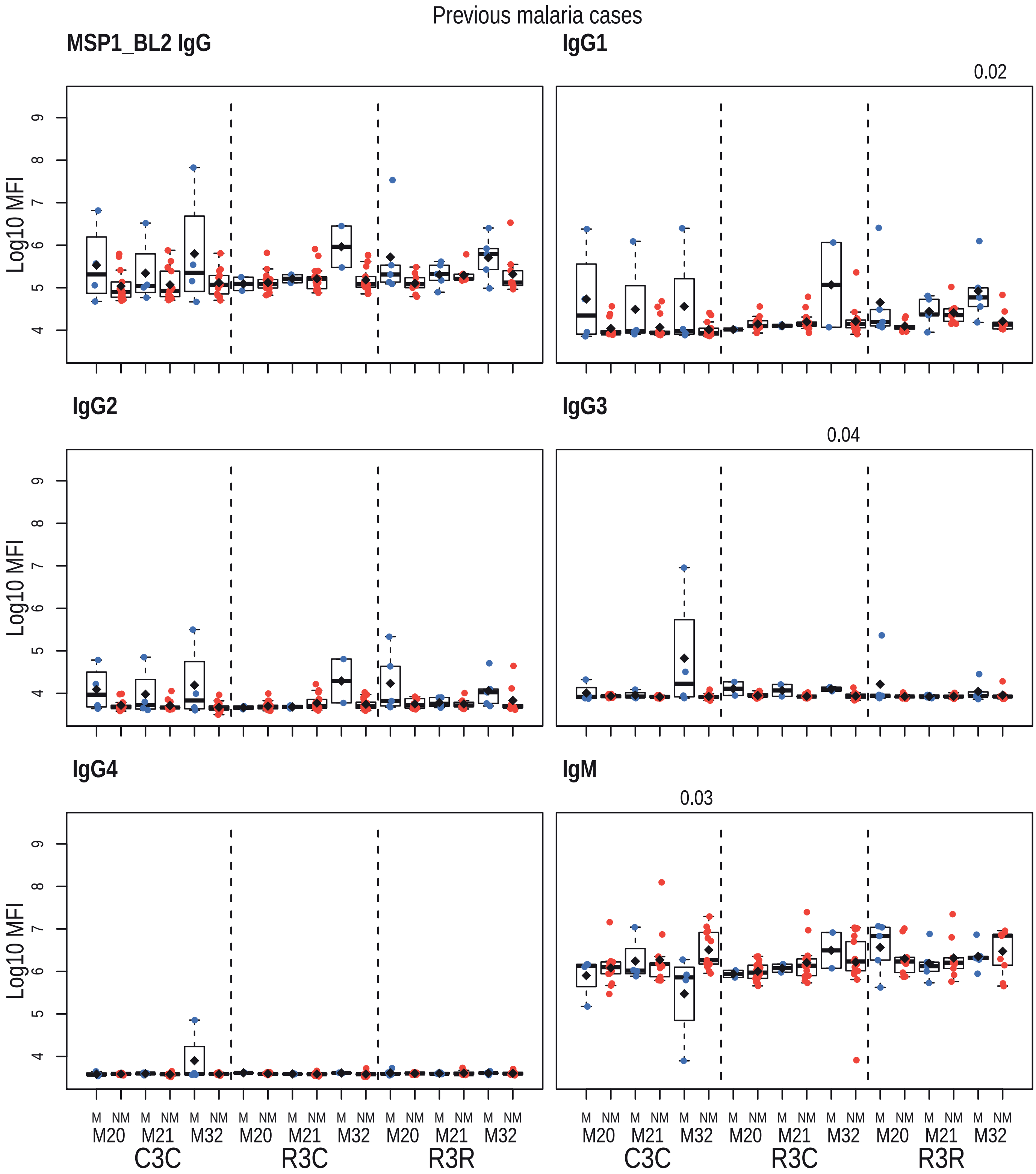

Supplementary Figure 27. Immunogenicity stratified by previous clinical malaria against blood stage antigens: Total IgG, IgG1-4 subclasses and IgM for MSP1 Block2 at month (M) 20, 21 and 32 for RTS,S/AS01 vaccinees with (R3R) and without (R3C) booster, and comparator (C3C). Stratified analysis by malaria cases before M20, subjects who presented with clinical malaria (M=blue) and subjects without clinical malaria (NM=red). Boxplots with medians, interquartile ranges (IQR), upper whisker as the smallest between maximum x value and Q3 + 1.5 * IQR, lower whisker as the largest between minimum x value and Q1 – 1.5 * IQR, and log10(geometric mean(MFI)) (diamond). Non-parametric tests were used to compare levels with or without clinical malaria (M vs NM). P-values were adjusted for multiple comparisons, but none was significant. Only p-values <0.05 before adjustment are shown. The y axis is in logarithm 10 scale. R3R: three doses of RTS,S/AS01E and a RTS,S/AS01E booster. R3C: three doses of RTS,S/AS01E and a comparator booster. C3C: three doses of a comparator vaccine and a comparator booster.

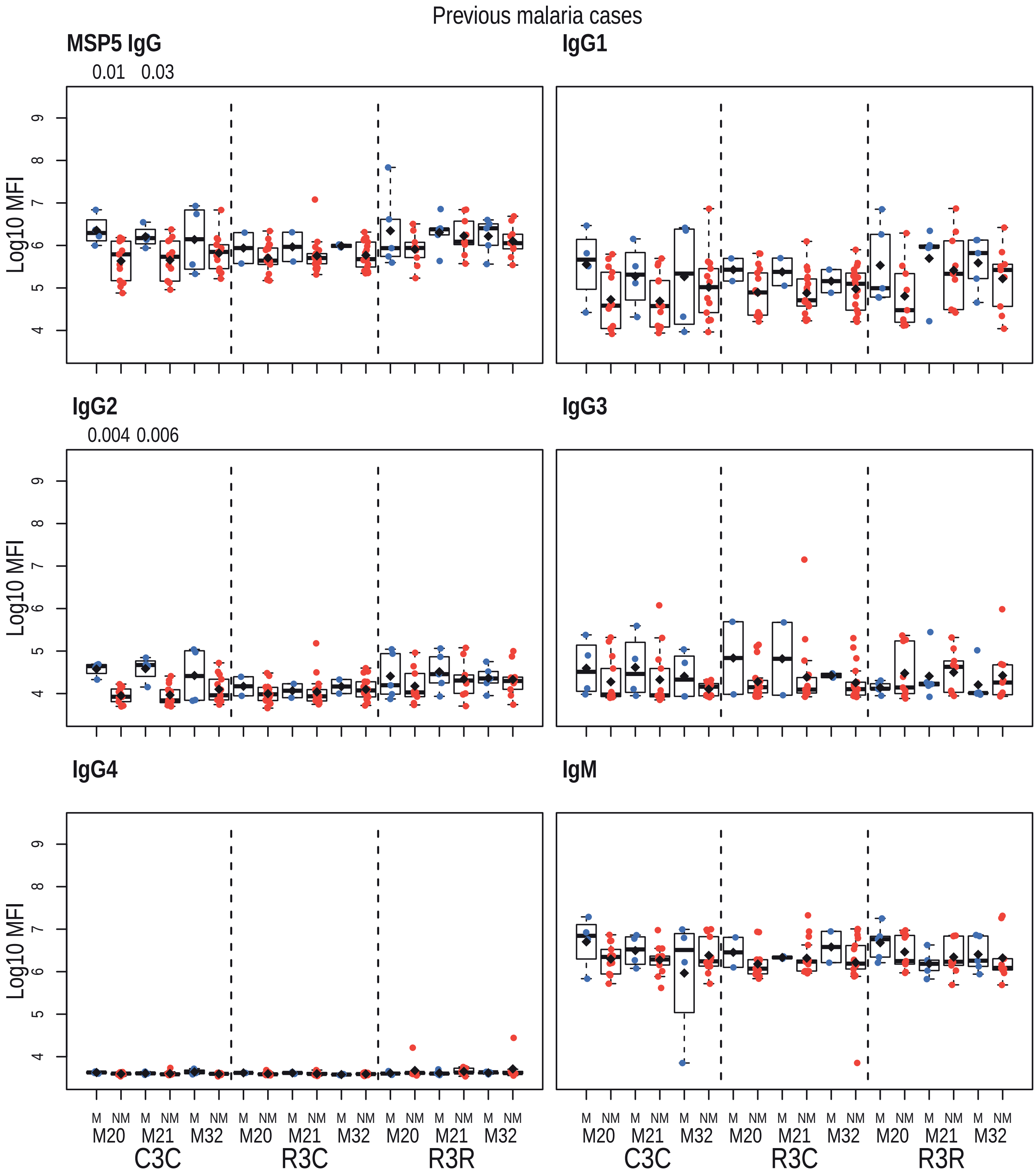

Supplementary Figure 28. Immunogenicity stratified by previous clinical malaria against blood stage antigens: Total IgG, IgG1-4 subclasses and IgM for MSP5 at month (M) 20, 21 and 32 for RTS,S/AS01 vaccinees with (R3R) and without (R3C) booster, and comparator (C3C). Stratified analysis by malaria cases before M20, subjects who presented with clinical malaria (M=blue) and subjects without clinical malaria (NM=red). Boxplots with medians, interquartile ranges (IQR), upper whisker as the smallest between maximum x value and Q3 + 1.5 * IQR, lower whisker as the largest between minimum x value and Q1 – 1.5 * IQR, and log10(geometric mean(MFI)) (diamond). Non-parametric tests were used to compare levels with or without clinical malaria (M vs NM). P-values were adjusted for multiple comparisons, but none was significant. Only p-values <0.05 before adjustment are shown. The y axis is in logarithm 10 scale. R3R: three doses of RTS,S/AS01E and a RTS,S/AS01E booster. R3C: three doses of RTS,S/AS01E and a comparator booster. C3C: three doses of a comparator vaccine and a comparator booster.

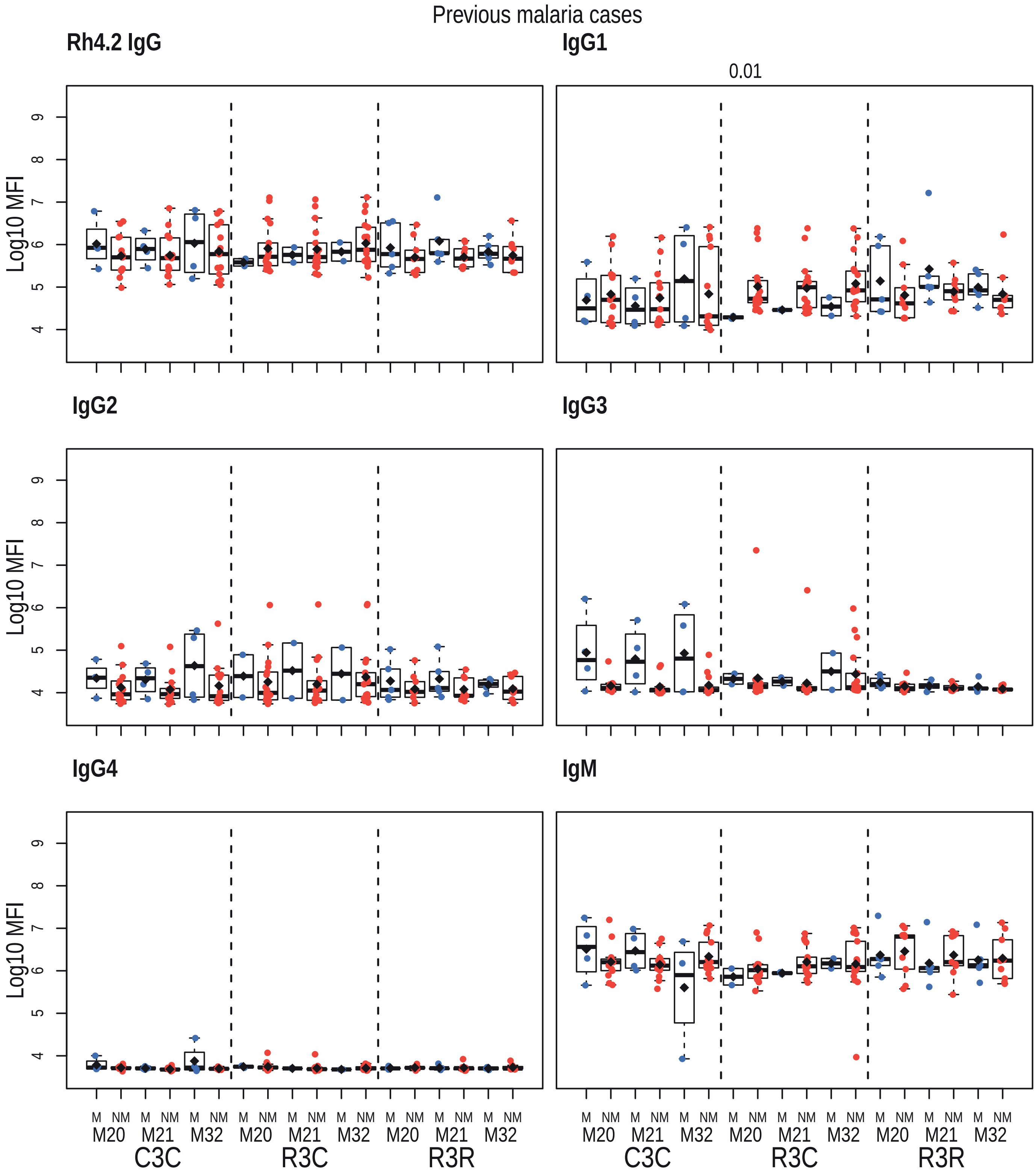

Supplementary Figure 29. Immunogenicity stratified by previous clinical malaria against blood stage antigens: Total IgG, IgG1-4 subclasses and IgM for Rh4.2 at month (M) 20, 21 and 32 for RTS,S/AS01 vaccinees with (R3R) and without (R3C) booster, and comparator (C3C). Stratified analysis by malaria cases before M20, subjects who presented with clinical malaria (M=blue) and subjects without clinical malaria (NM=red). Boxplots with medians, interquartile ranges (IQR), upper whisker as the smallest between maximum x value and Q3 + 1.5 * IQR, lower whisker as the largest between minimum x value and Q1 – 1.5 * IQR, and log10(geometric mean(MFI)) (diamond). Non-parametric tests were used to compare levels with or without clinical malaria (M vs NM). P-values were adjusted for multiple comparisons, but none was significant. Only p-values <0.05 before adjustment are shown. The y axis is in logarithm 10 scale. R3R: three doses of RTS,S/AS01E and a RTS,S/AS01E booster. R3C: three doses of RTS,S/AS01E and a comparator booster. C3C: three doses of a comparator vaccine and a comparator booster.

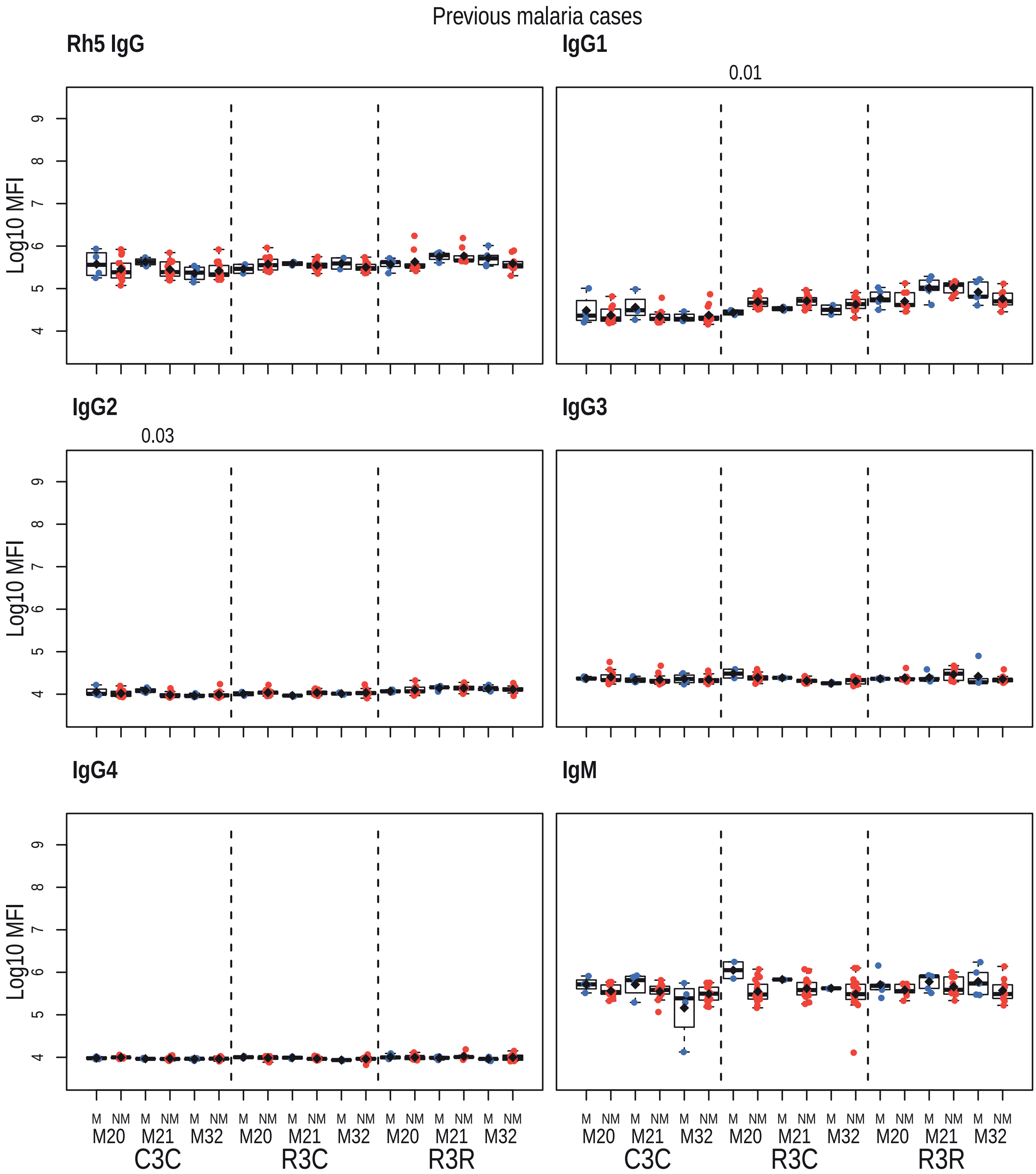

Supplementary Figure 30. Immunogenicity stratified by previous clinical malaria against blood stage antigens: Total IgG, IgG1-4 subclasses and IgM for Rh5 at month (M) 20, 21 and 32 for RTS,S/AS01 vaccinees with (R3R) and without (R3C) booster, and comparator (C3C). Stratified analysis by malaria cases before M20, subjects who presented with clinical malaria (M=blue) and subjects without clinical malaria (NM=red). Boxplots with medians, interquartile ranges (IQR), upper whisker as the smallest between maximum x value and Q3 + 1.5 * IQR, lower whisker as the largest between minimum x value and Q1 – 1.5 * IQR, and log10(geometric mean(MFI)) (diamond). Non-parametric tests were used to compare levels with or without clinical malaria (M vs NM). P-values were adjusted for multiple comparisons, but none was significant. Only p-values <0.05 before adjustment are shown. The y axis is in logarithm 10 scale. R3R: three doses of RTS,S/AS01E and a RTS,S/AS01E booster. R3C: three doses of RTS,S/AS01E and a comparator booster. C3C: three doses of a comparator vaccine and a comparator booster.

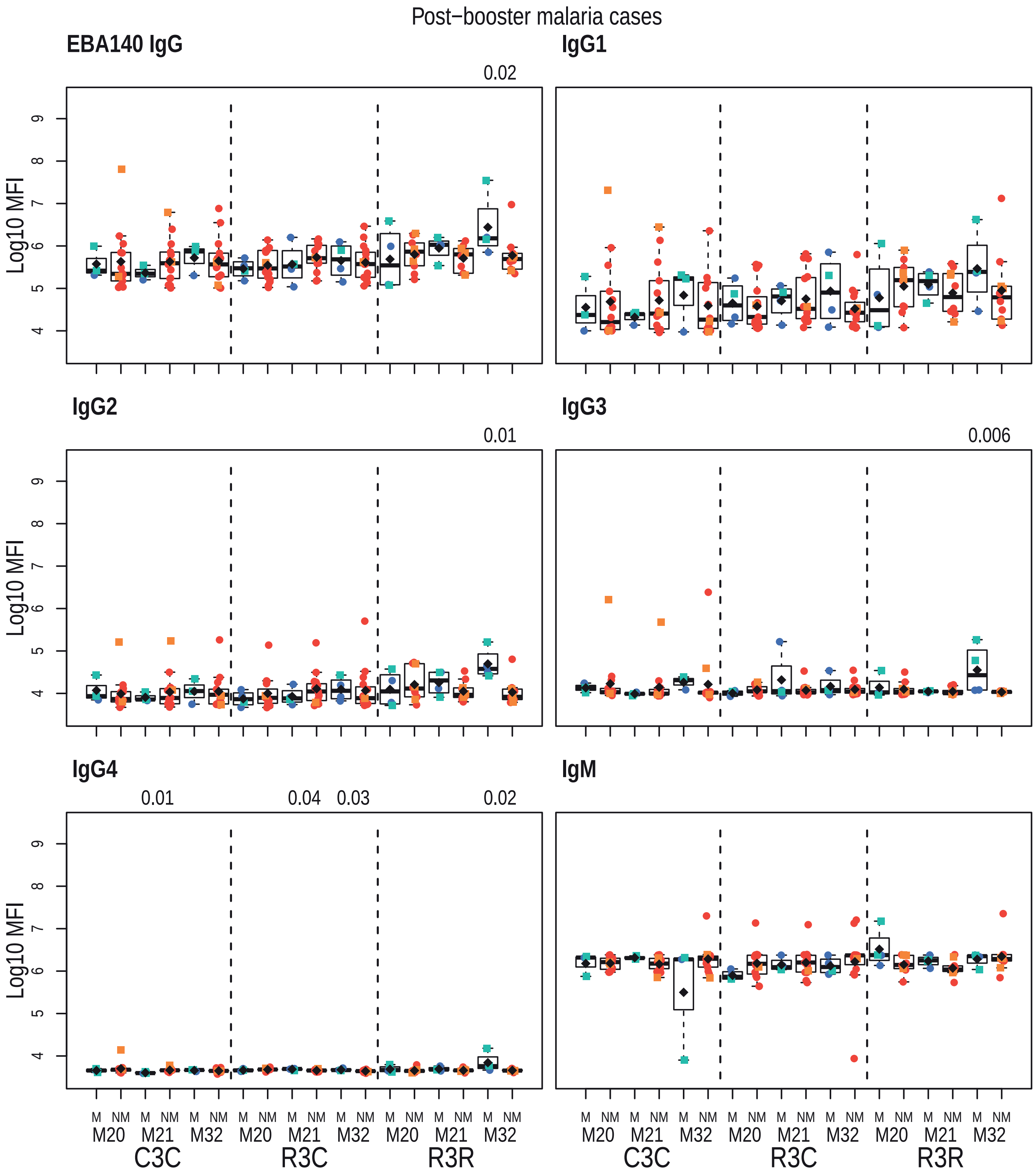

Supplementary Figure 31. Immunogenicity stratified by clinical malaria after M21 against blood stage antigens: Total IgG, IgG1-4 subclasses and IgM for EBA140 at month (M) 20, 21 and 32 for RTS,S/AS01 vaccinees with (R3R) and without (R3C) booster, and comparator (C3C). Stratified analysis by malaria after M21, subjects who presented with clinical malaria (M=blue) and subjects without clinical malaria (NM=red). Those who presented with clinical malaria before M20 are represented with green and orange squares. Boxplots with medians, interquartile ranges (IQR), upper whisker as the smallest between maximum x value and Q3 + 1.5 * IQR, lower whisker as the largest between minimum x value and Q1 – 1.5 * IQR, and log10(geometric mean(MFI)) (diamond). Non-parametric tests were used to compare levels with or without clinical malaria (NM vs M). P-values were adjusted for multiple comparisons, but none was significant. Only p-values <0.05 before adjustment are shown. The y axis is in logarithm 10 scale. R3R: three doses of RTS,S/AS01E and a RTS,S/AS01E booster. R3C: three doses of RTS,S/AS01E and a comparator booster. C3C: three doses of a comparator vaccine and a comparator booster.

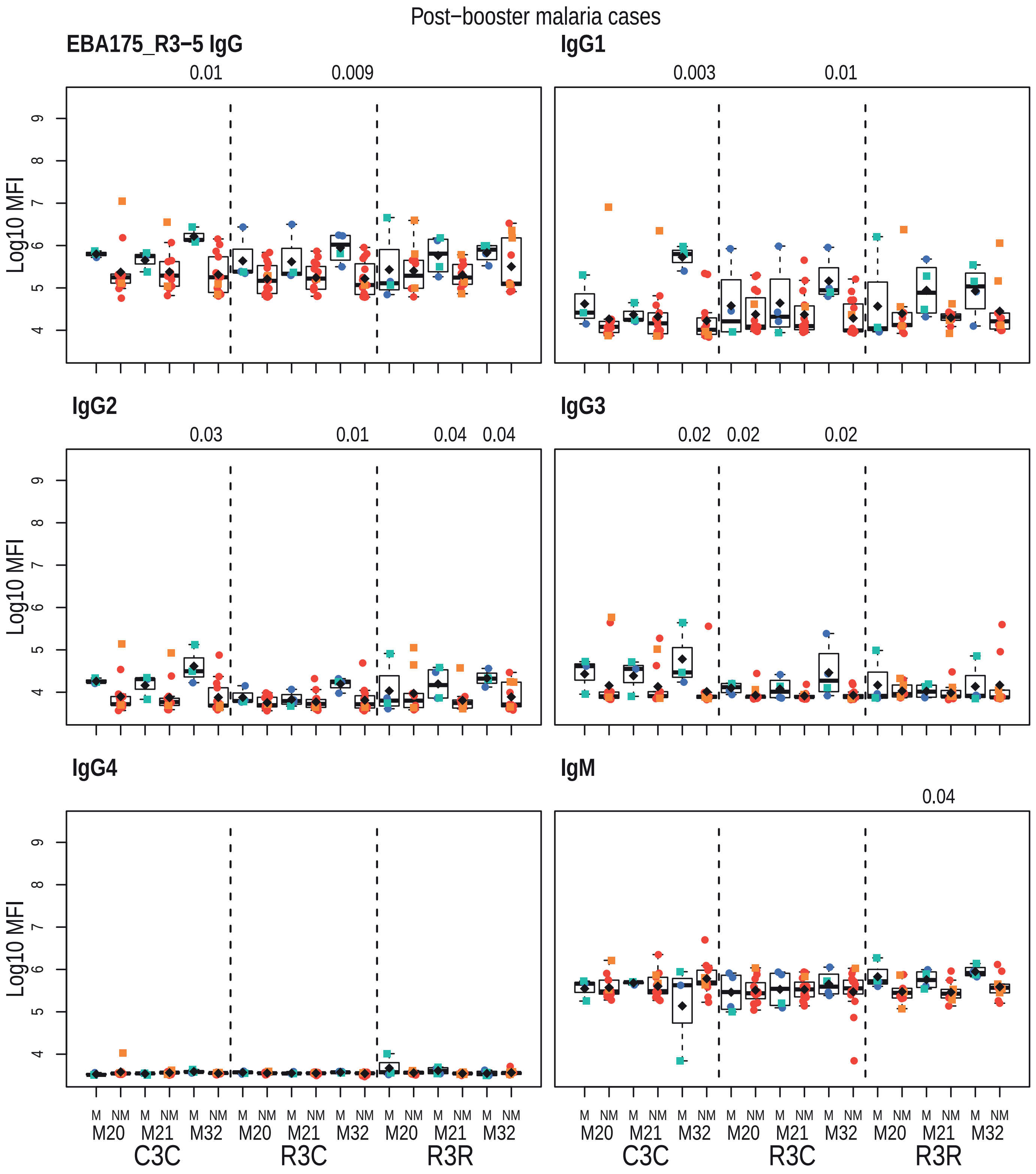

Supplementary Figure 32. Immunogenicity stratified by clinical malaria after M21 against blood stage antigens: Total IgG, IgG1-4 subclasses and IgM for EBA175 R3-5 at month (M) 20, 21 and 32 for RTS,S/AS01 vaccinees with (R3R) and without (R3C) booster, and comparator (C3C). Stratified analysis by malaria after M21, subjects who presented with clinical malaria (M=blue) and subjects without clinical malaria (NM=red). Those who presented with clinical malaria before M20 are represented with green and orange squares. Boxplots with medians, interquartile ranges (IQR), upper whisker as the smallest between maximum x value and Q3 + 1.5 * IQR, lower whisker as the largest between minimum x value and Q1 – 1.5 * IQR, and log10(geometric mean(MFI)) (diamond). Non-parametric tests were used to compare levels with or without clinical malaria (NM vs M). P-values were adjusted for multiple comparisons, but none was significant. Only p-values <0.05 before adjustment are shown. The y axis is in logarithm 10 scale. R3R: three doses of RTS,S/AS01E and a RTS,S/AS01E booster. R3C: three doses of RTS,S/AS01E and a comparator booster. C3C: three doses of a comparator vaccine and a comparator booster.

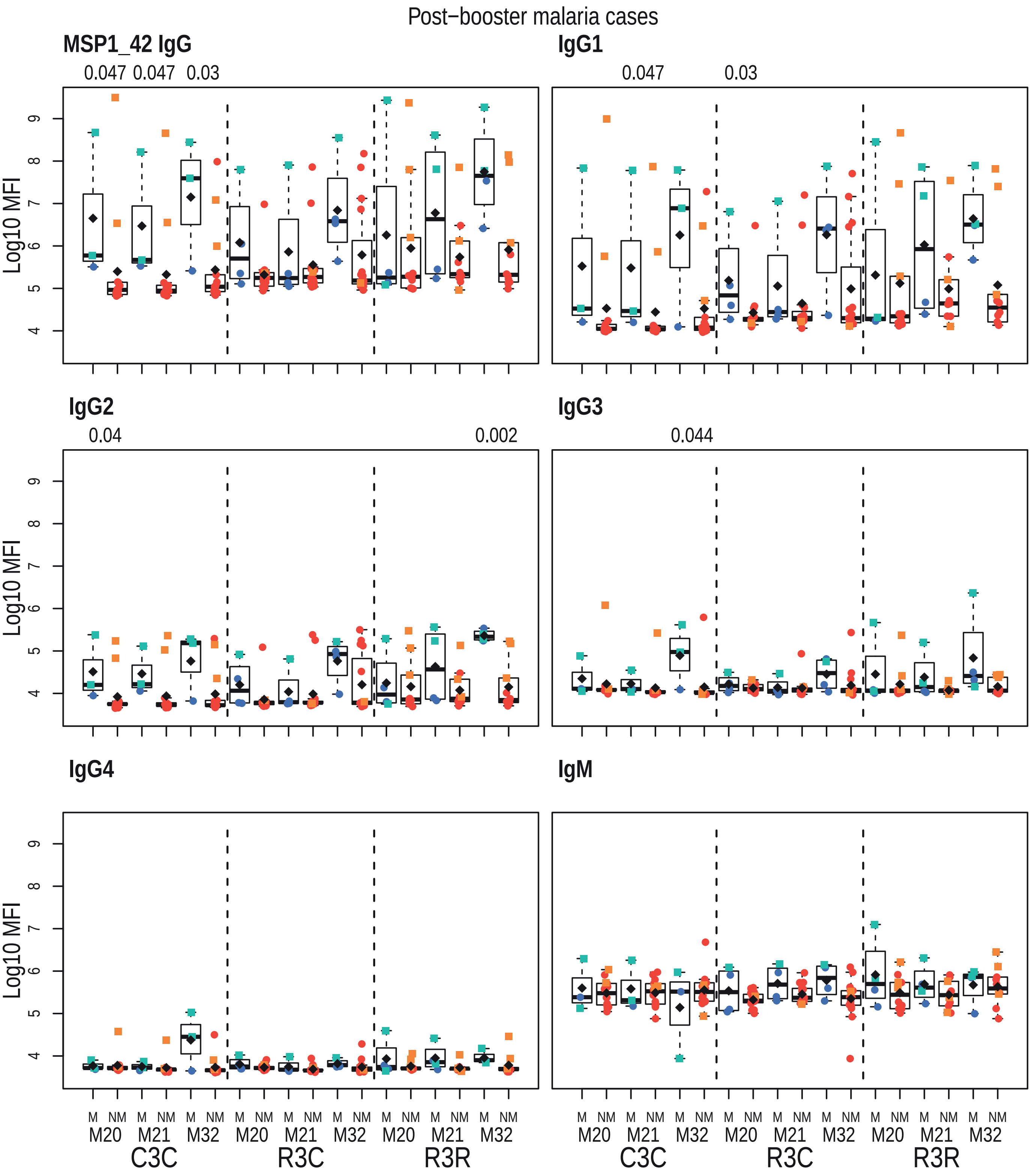

Supplementary Figure 33. Immunogenicity stratified by clinical malaria after M21 against blood stage antigens: Total IgG, IgG1-4 subclasses and IgM for $MSP1_{42}$ at month (M) 20, 21 and 32 for RTS,S/AS01 vaccinees with (R3R) and without (R3C) booster, and comparator (C3C). Stratified analysis by malaria after M21, subjects who presented with clinical malaria (M=blue) and subjects without clinical malaria (NM=red). Those who presented with clinical malaria before M20 are represented with green and orange squares. Boxplots with medians, interquartile ranges (IQR), upper whisker as the smallest between maximum x value and Q3 + 1.5 * IQR, lower whisker as the largest between minimum x value and Q1 – 1.5 * IQR, and log10(geometric mean(MFI)) (diamond). Non-parametric tests were used to compare levels with or without clinical malaria (NM vs M). P-values were adjusted for multiple comparisons, but none was significant. Only p-values <0.05 before adjustment are shown. The y axis is in logarithm 10 scale. R3R: three doses of RTS,S/AS01E and a RTS,S/AS01E booster. R3C: three doses of RTS,S/AS01E and a comparator booster. C3C: three doses of a comparator vaccine and a comparator booster-

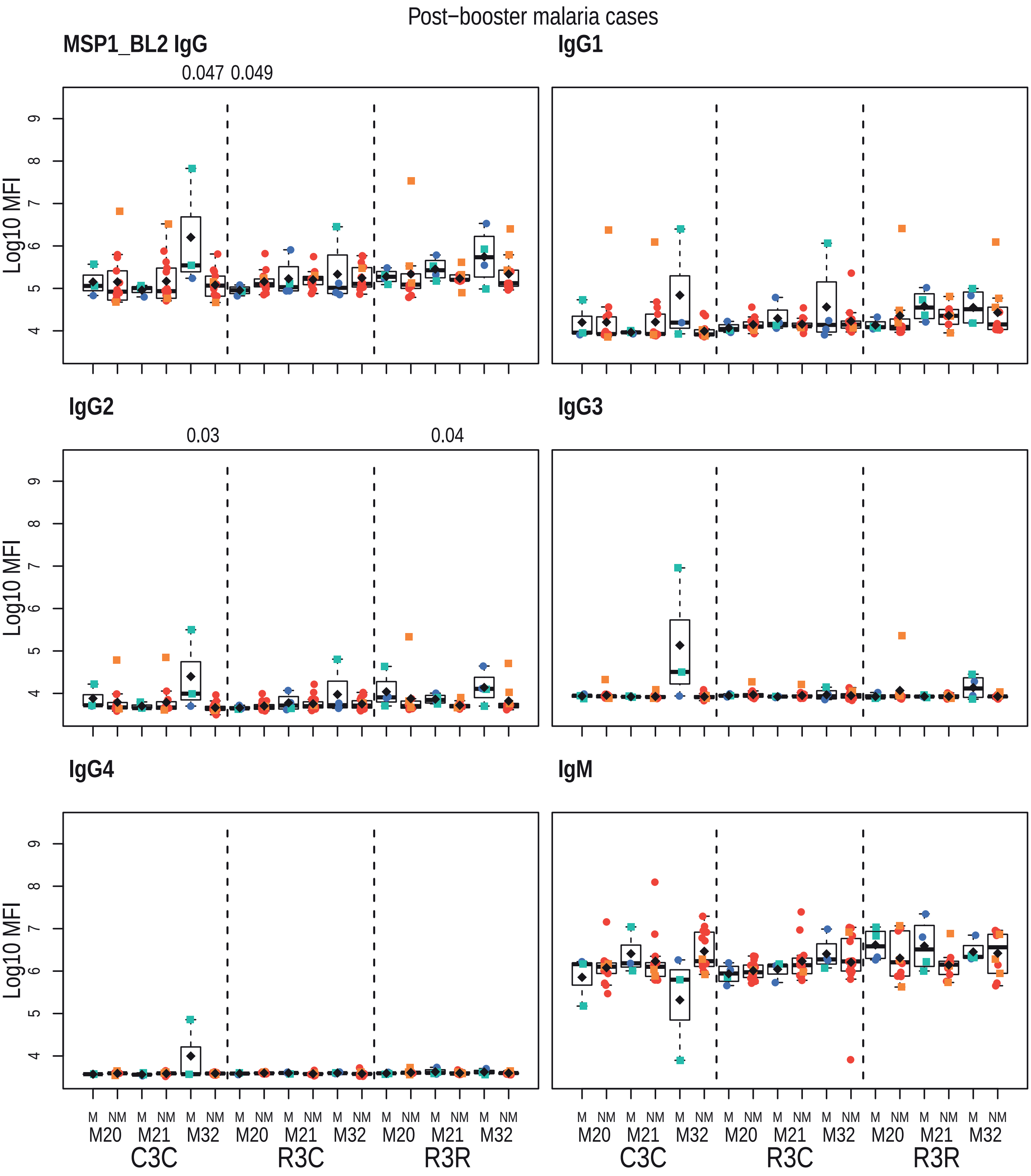

Supplementary Figure 34. Immunogenicity stratified by clinical malaria after M21 against blood stage antigens: Total IgG, IgG1-4 subclasses and IgM for MSP1 Block 2 at month (M) 20, 21 and 32 for RTS,S/AS01 vaccinees with (R3R) and without (R3C) booster, and comparator (C3C). Stratified analysis by malaria after M21, subjects who presented with clinical malaria (M=blue) and subjects without clinical malaria (NM=red). Those who presented with clinical malaria before M20 are represented with green and orange squares. Boxplots with medians, interquartile ranges (IQR), upper whisker as the smallest between maximum x value and Q3 + 1.5 * IQR, lower whisker as the largest between minimum x value and Q1 – 1.5 * IQR, and log10(geometric mean(MFI)) (diamond). Non-parametric tests were used to compare levels with or without clinical malaria (NM vs M). P-values were adjusted for multiple comparisons, but none was significant. Only p-values <0.05 before adjustment are shown. The y axis is in logarithm 10 scale. R3R: three doses of RTS,S/AS01E and a RTS,S/AS01E booster. R3C: three doses of RTS,S/AS01E and a comparator booster. C3C: three doses of a comparator vaccine and a comparator booster.

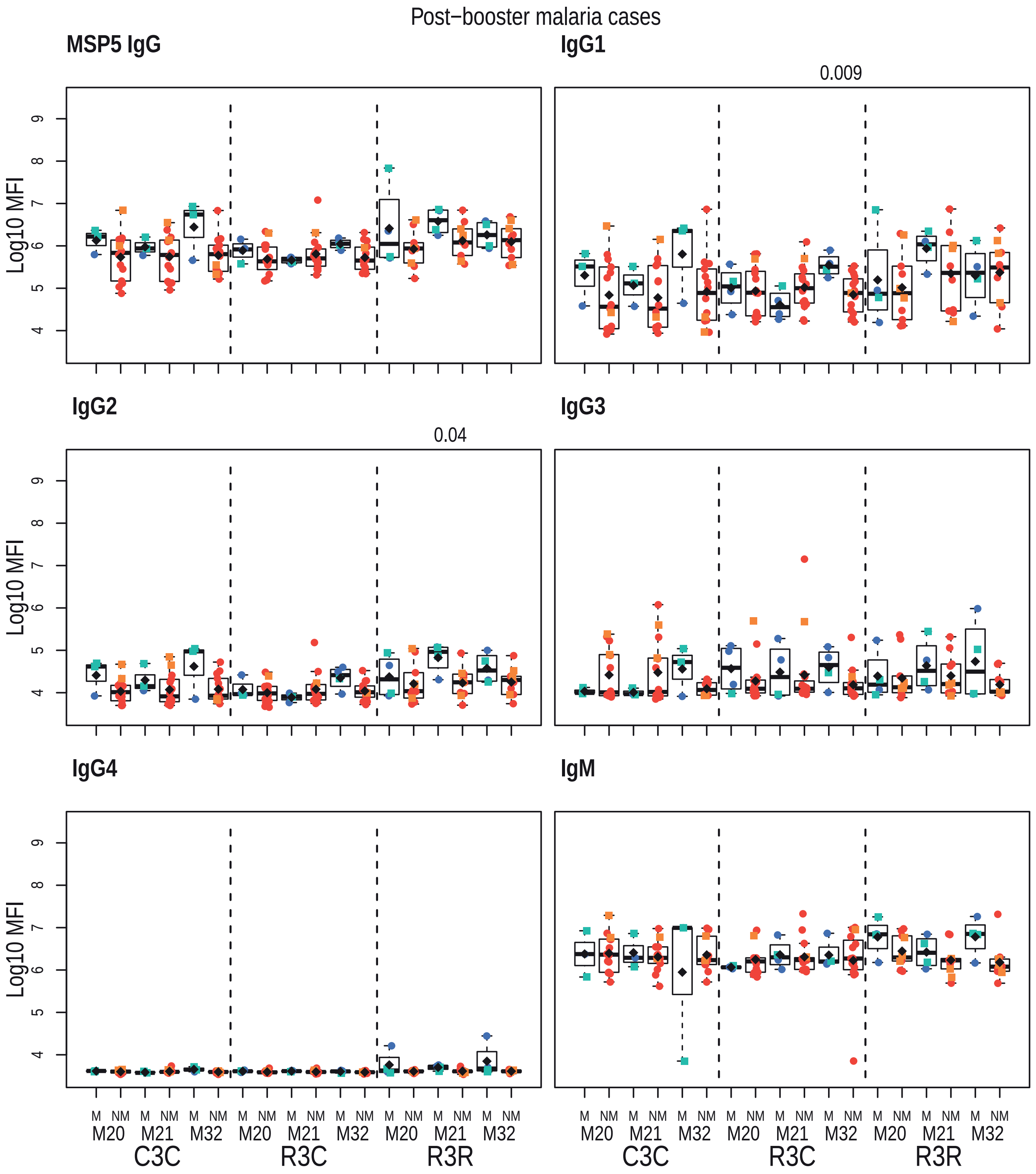

Supplementary Figure 35. Immunogenicity stratified by clinical malaria after M21 against blood stage antigens: Total IgG, IgG1-4 subclasses and IgM for MSP5 at month (M) 20, 21 and 32 for RTS,S/AS01 vaccinees with (R3R) and without (R3C) booster, and comparator (C3C). Stratified analysis by malaria after M21, subjects who presented with clinical malaria (M=blue) and subjects without clinical malaria (NM=red). Those who presented with clinical malaria before M20 are represented with green and orange squares. Boxplots with medians, interquartile ranges (IQR), upper whisker as the smallest between maximum x value and Q3 + 1.5 * IQR, lower whisker as the largest between minimum x value and Q1 – 1.5 * IQR, and log10(geometric mean(MFI)) (diamond). Non-parametric tests were used to compare levels with or without clinical malaria (NM vs M). P-values were adjusted for multiple comparisons, but none was significant. Only p-values <0.05 before adjustment are shown. The y axis is in logarithm 10 scale. R3R: three doses of RTS,S/AS01E and a RTS,S/AS01E booster. R3C: three doses of RTS,S/AS01E and a comparator booster. C3C: three doses of a comparator vaccine and a comparator booster.

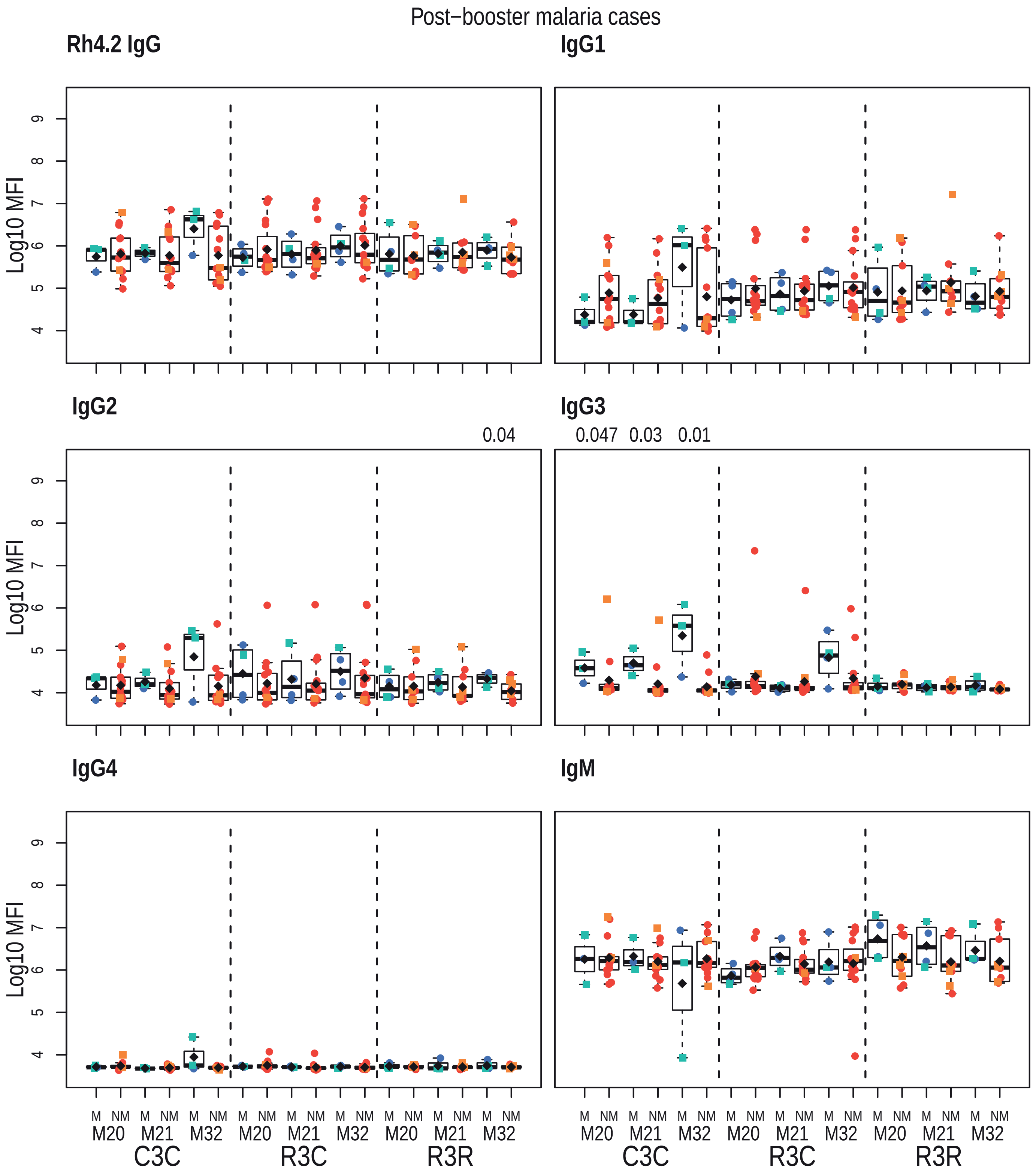

Supplementary Figure 36. Immunogenicity stratified by clinical malaria after M21 against blood stage antigens: Total IgG, IgG1-4 subclasses and IgM for Rh4.2 at month (M) 20, 21 and 32 for RTS,S/AS01 vaccinees with (R3R) and without (R3C) booster, and comparator (C3C). Stratified analysis by malaria after M21, subjects who presented with clinical malaria (M=blue) and subjects without clinical malaria (NM=red). Those who presented with clinical malaria before M20 are represented with green and orange squares. Boxplots with medians, interquartile ranges (IQR), upper whisker as the smallest between maximum x value and Q3 + 1.5 * IQR, lower whisker as the largest between minimum x value and Q1 – 1.5 * IQR, and log10(geometric mean(MFI)) (diamond). Non-parametric tests were used to compare levels with or without clinical malaria (NM vs M). P-values were adjusted for multiple comparisons, but none was significant. Only p-values <0.05 before adjustment are shown. The y axis is in logarithm 10 scale. R3R: three doses of RTS,S/AS01E and a RTS,S/AS01E booster. R3C: three doses of RTS,S/AS01E and a comparator booster. C3C: three doses of a comparator vaccine and a comparator booster.

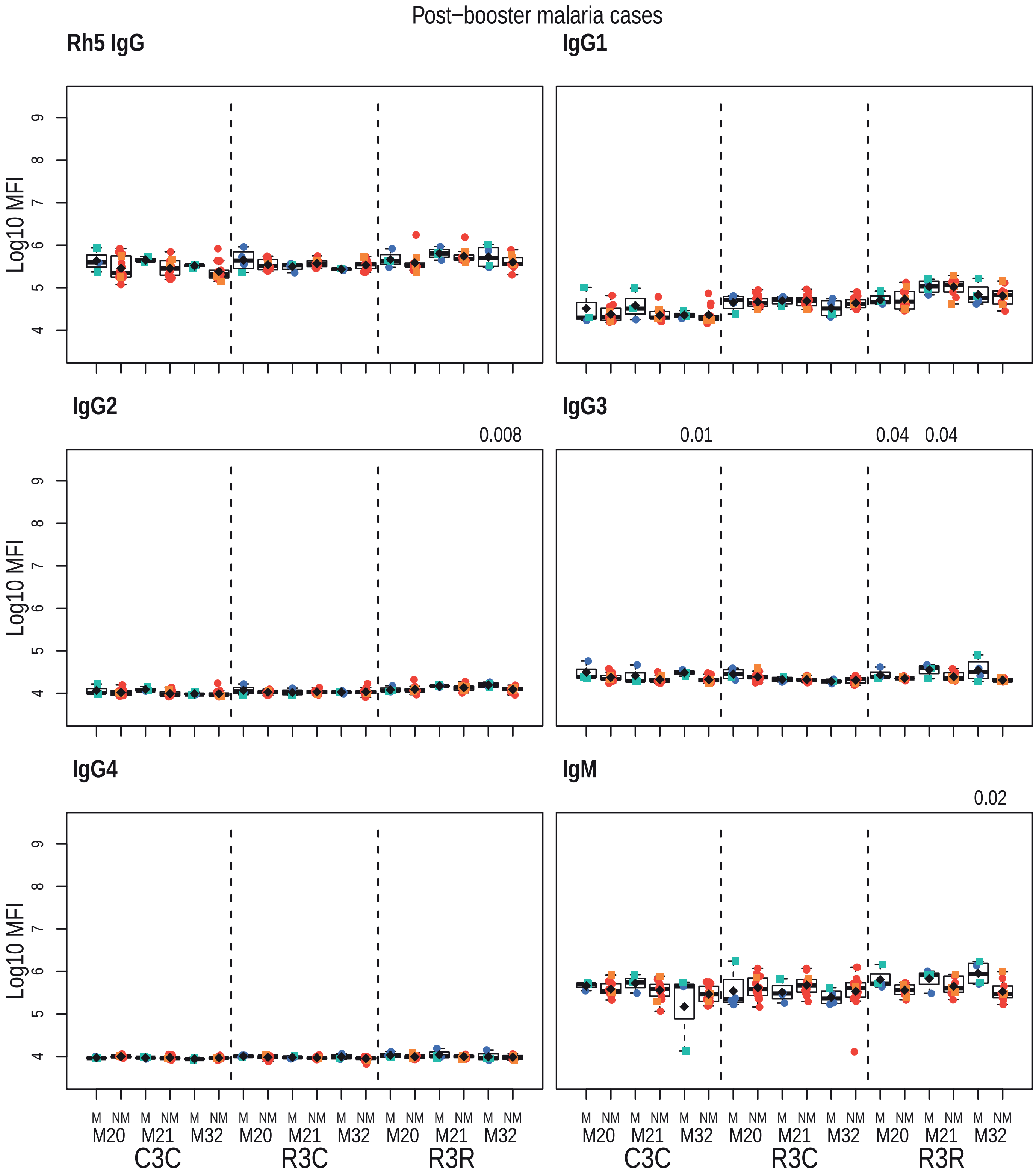

Supplementary Figure 37. Immunogenicity stratified by clinical malaria after M21 against blood stage antigens: Total IgG, IgG1-4 subclasses and IgM for Rh5 at month (M) 20, 21 and 32 for RTS,S/AS01 vaccinees with (R3R) and without (R3C) booster, and comparator (C3C). Stratified analysis by malaria after M21, subjects who presented with clinical malaria (M=blue) and subjects without clinical malaria (NM=red). Those who presented with clinical malaria before M20 are represented with green and orange squares. Boxplots with medians, interquartile ranges (IQR), upper whisker as the smallest between maximum x value and Q3 + 1.5 * IQR, lower whisker as the largest between minimum x value and Q1 – 1.5 * IQR, and log10(geometric mean(MFI)) (diamond). Non-parametric tests were used to compare levels with or without clinical malaria (NM vs M). P-values were adjusted for multiple comparisons, but none was significant. Only p-values <0.05 before adjustment are shown. The y axis is in logarithm 10 scale. R3R: three doses of RTS,S/AS01E and a RTS,S/AS01E booster. R3C: three doses of RTS,S/AS01E and a comparator booster. C3C: three doses of a comparator vaccine and a comparator booster .

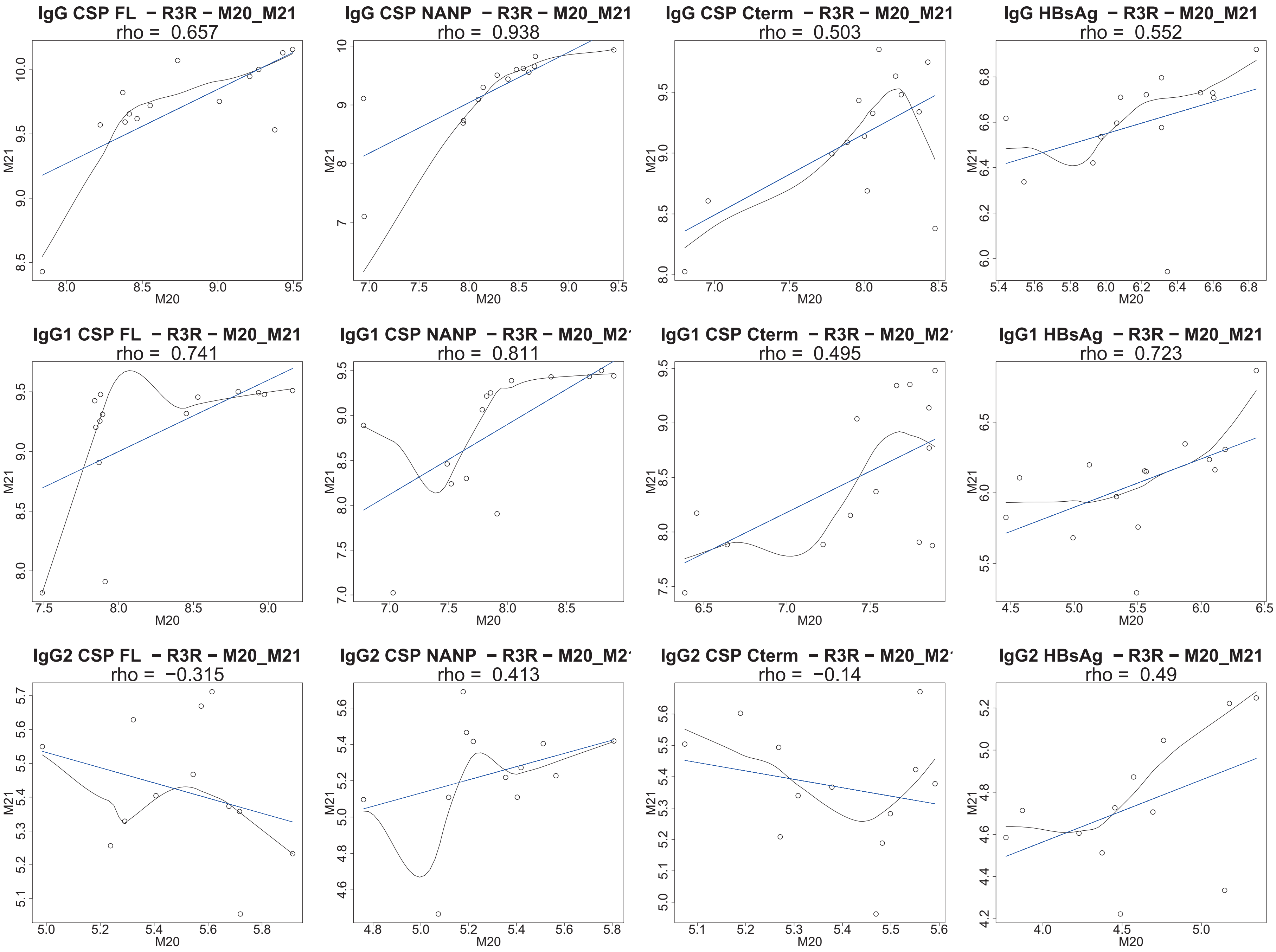

Supplementary Figure 38. Correlation plots between M20 and M21 for CSP constructs and HBsAg for IgG, IgG1-2: : linear regression (blue line), LOESS curve fitting (black line).

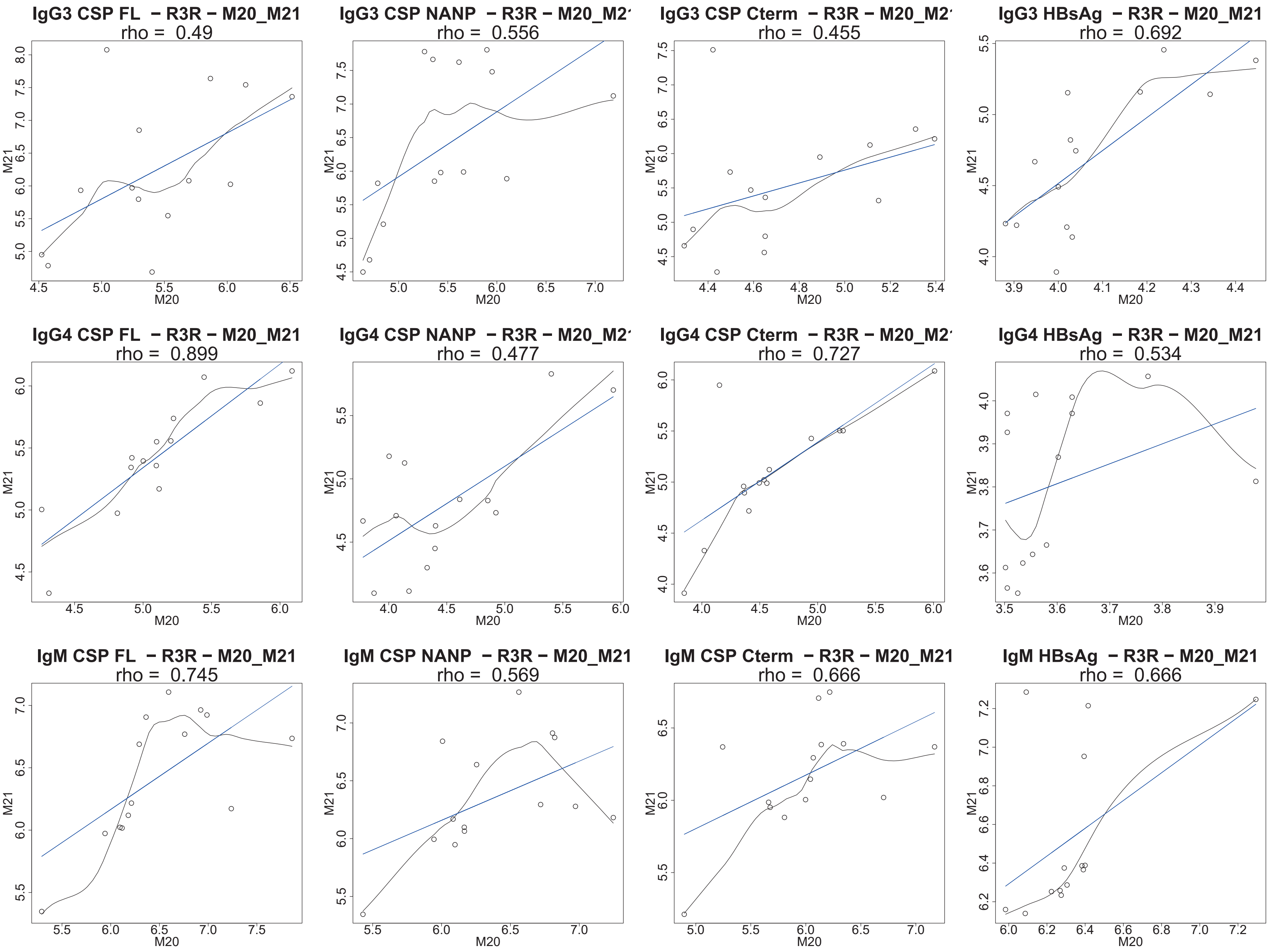

Supplementary Figure 39. Correlation plots between M20 and M21 for CSP constructs and HBsAg for IgG3-4 and IgM: : linear regression (blue line), LOESS curve fitting (black line).

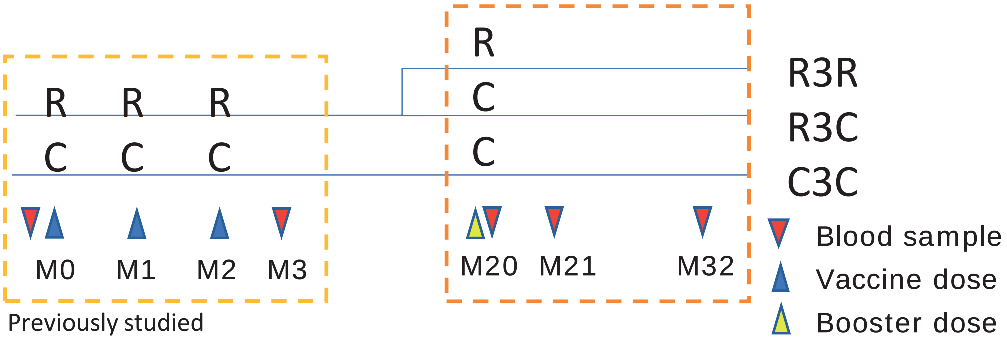

Supplementary Figure 40. Study design, vaccination and blood sampling schedule.

Supplementary Table 7. Study population demographic characteristics.

Study Population						
	**R3R**		**R3C**		**C3C**	
	N=14		N=19		N=17	
	n	%	n	%	n	%
Sex						
Female	4	28.6	7	36.8	9	52.9
Male	10	71.4	12	63.2	8	47.1
Age						
5-17 months	6	42.9	9	47.4	9	52.9
6-12 weeks	8	57.1	10	52.6	8	47.1
Malaria cases						
Before booster (pre-M20)	5	35.7	2	10.5	4	23.5
After booster (post-M20)	4	28.6	4	21.1	3	17.6
Malaria transmission in Manhiça was low [1,2].						
HIV infection was not a protocol exclusion/inclusion criterion, but only healthy children were included in the study. HIV testing was not a trial procedure. The prevalence of HIV infection in adults in the Manhiça area was around 40% [3].						
R3R: three doses of RTS,S/AS01E and a RTS,S/AS01E booster at month 20.<br>R3C: three doses of RTS,S/AS01E and a comparator booster.<br>C3C: three doses of a comparator vaccine and a comparator booster. M: month.						

Supplementary Table 8. Antigens coupled to the multiplex beads.

Antigen	Description		Sequence	Tag
**CSP Full Length** [4]	Circumsporozoite Protein	Vaccine components	YGSSSNTRVLNELNYDNAGTNLYNELEMNYYGKQENWYSLKKNSRSLGENDDGNNEDNEKLRKPKHKKLKQPADGNPDPNANPNVDPNANPNVDPNANPNVDPNANPNANPNANPNANPNANPNANPNANPNANPNANPNANPNANPNANPNANPNANPNANPNANPNKNNQGNGQGHNMPNDPNRNVDENANANSAVKNNNNEEPSDKHIKEYLNKIQNSLSTEWSPCSVTCGNGIQVRIKPGSANKPKDELDYANDIEKKICKMEK	Histidine
**CSP C-terminus** [5]	Circumsporozoite Protein	Vaccine components	KNNQGNGQGHNMPNDPNRNVDENANANSAVKNNNNEEPSDKHIKEYQNKIQNSLSTEWSPCSVTCGNGIQVRIKPGSANKPKDELDYANDIEKKICKMEK	GST
**CSP $NANP_6$** [6]	Circumsporozoite Protein	Vaccine components	NANPNVDPNANPNVDPNANPNVDPNANPNANPNANPNANPNANPNANPNANPNANPNANPNANPNANPNANPNANPNANPNANP	GST
**HBsAg**	Hepatitis B surface Antigen	Vaccine components	Source: Abcam	
**$MSP1_{42}$** [7]	Merozoite surface protein	Blood stage antigens	MAHHHHHHPGGSGSGTMAISVTMDNILSGFENEYDVIYLKPLAGVYRSLKKQIEKNIFTFNLNLNDILNSRLKKRKYFLDVLESDLMQFKHISSNEYIIEDSFKLLNSEQKNTLLKSYKYIKESVENDIKFAQEGISYYEKVLAKYKDDLESIKKVIKEEKEKFPSSPPTTPPSPAKTDEQKKESKFLPFLTNIETLYNNLVNKIDDYLINLKAKINDCNVEKDEAHVKITKLSDLKAIDDKIDLFKNPTDFEAIKKLINDDTKKDMLGKLLSTGLVQNFPNTIISKLIEGKFQDMLNISQHQCVKKQCPENSGCFRHLDEREECKCLLNYKQEGDKCVENPNPTCNENNGGCDADATCTEEDSGSSRKKITCECTKPDSYPLFDGIFCS	Histidine
**Rh5** [8]	Reticulocyte binding protein	Blood stage antigens	NAIKKTKNQENNLTLLPIKSTEEEKDDIKNGKDIKKEIDNDKENIKTNNAKDHSTYIKSYLNTNVNDGLKYLFIPSHNSFIKKYSVFNQINDGMLLNEKNDVKNNEDYKNVDYKNVNFLQYHFKELSNYNIANSIDILQEKEGHLDFVIIPHYTFLDYYKHLSYNSIYHKSSTYGKCIAVDAFIKKINETYDKVKSKCNDIKNDLIATIKKLEHPYDINNKNDDSYRYDISEEIDDKSEETDDETEEVEDSIQDTDSNHTPSNKKKNDLMNRTFKKMMDEYNTKKKKLIKCIKNHENDFNKICMDMKNYGTNLFEQLSCYNNNFCNTNGIRYHYDEYIHKLILSVKSKNLNKDLSDMTNILQQSELLLTNLNKKMGSYIYIDTIKFIHKEMKHIFNRIEYHTKIINDKTKIIQDKIKLNIWRTFQKDELLKRILDMSNEYSLFITSDHLRQMLYNTFYSKEKHLNNIFHHLIYVLQMKFNDVPIKMEYFQTYKKNKPLTQ	Histidine
**EBA175 region 3-5** [9]	Erythrocyte binding antigen	Blood stage antigens	Amino acid residues 761–1298 (*Plasmodium falciparum* 3D7 genotype)	GST
**EBA140 region 3-5** [9]	Erythrocyte binding antigen	Blood stage antigens	Amino acid residues 770–1064 (*P. falciparum* 3D7)	GST
**MSP5** [10]	Merozoite surface protein	Blood stage antigens	MNILCILSYIYFFVIFYSLNLNNKNENFLVVRRLMNDEKGEGGFTSKNKENGNNNRNNENELKEEGSLPTKMNEKNSNSSDKQPNDISHDESKSNSNNSQNIQKEPEEKENSNPNLDSSENSSESATRSVDISEHNSNNPETKEENGEEPLDLEINENAEIGQEPPNRLHFDNVDDEVPHYSALRYNKVEKNVTDEMLLYNMMSDQNRKSCAINNGGCSDDQICININNIGVKCICKDGYLLGTKCIILNSYSCHPFFSILIYITLFLLLFV	Histidine
**Rh 4.2** [11]	Reticulocyte binding protein	Blood stage antigens	Amino acid residues 1277-1451 (*P. falciparum* 3D7)	GST
**MSP1 Block 2** [12]	Merozoite surface protein	Blood stage antigens	NEEEITTKGASAQSGASAQSGASAQSGASAQSGASAQSGASAQSGTSGPSGPSGT SPSSRSNTLPRSNTSSGASPPADAS	GST
**GST**	For fusion proteins	Tag	MSPILGYWKIKGLVQPTRLLLEYLEEKYEEHLYERDEGDKWRNKKFELGLEFPNLPYYIDGDVKLTQSMAIIRYIADKHNMLGGCPKERAEISMLEGAVLDIRYGVSRIAYSKDFETLKVDFLSKLPEMLKMFEDRLCHKTYLNGDHVTHPDFMLYDALDVVLYMDPMCLDAFPKLVCFKKRIEAIPQIDKYLKSSKYIAWPLQGWQATFGGGDHPPKSDLEVLFQGPLGS	

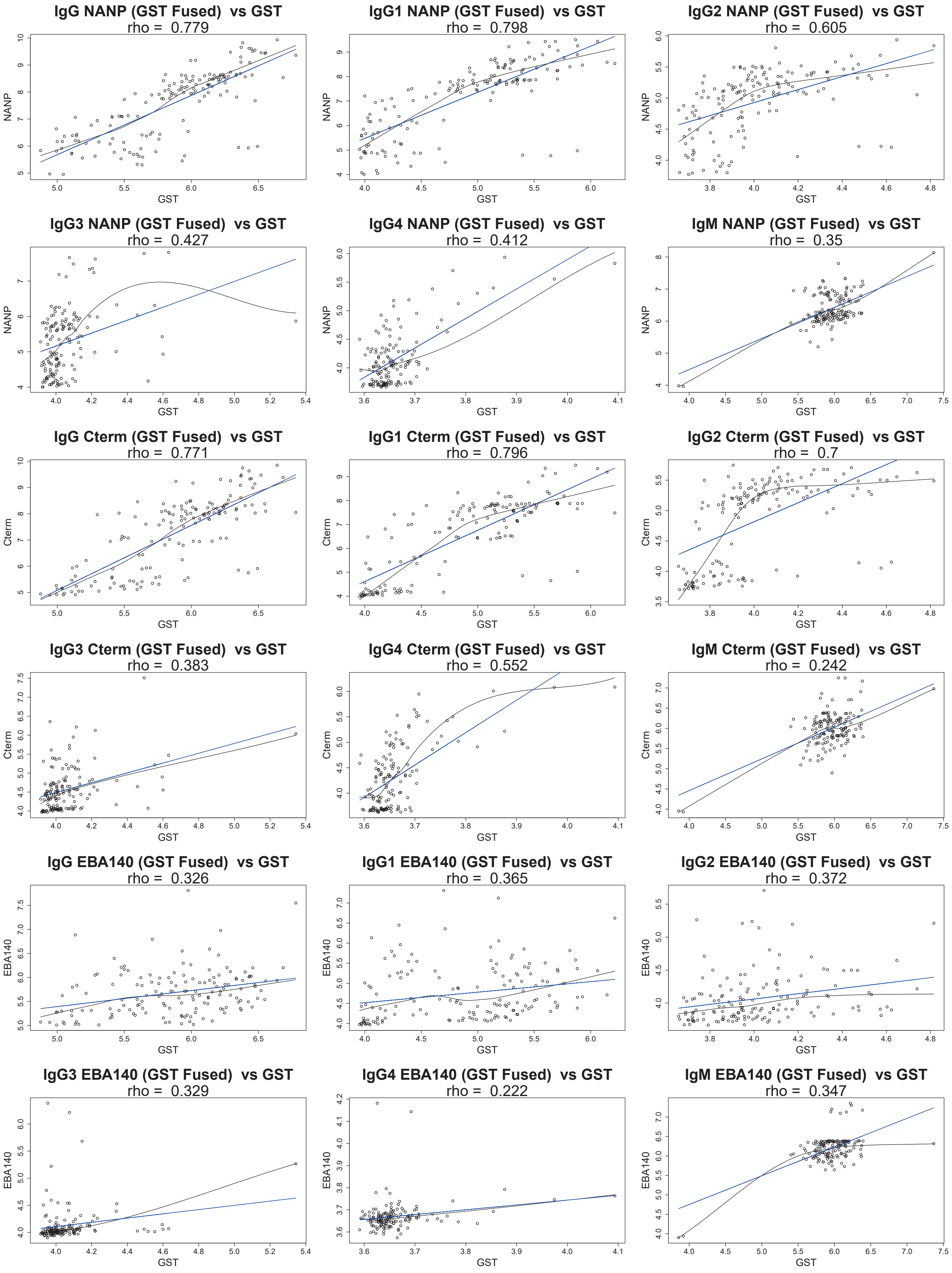

Supplementary Figure 41. Correlation plots for antibody response to IgG, IgG1-4, IgM against GST fused antigens (CSP NANP,CSP Cterm, EBA140) vs GST: linear regression (blue line), LOESS curve fitting (black line).

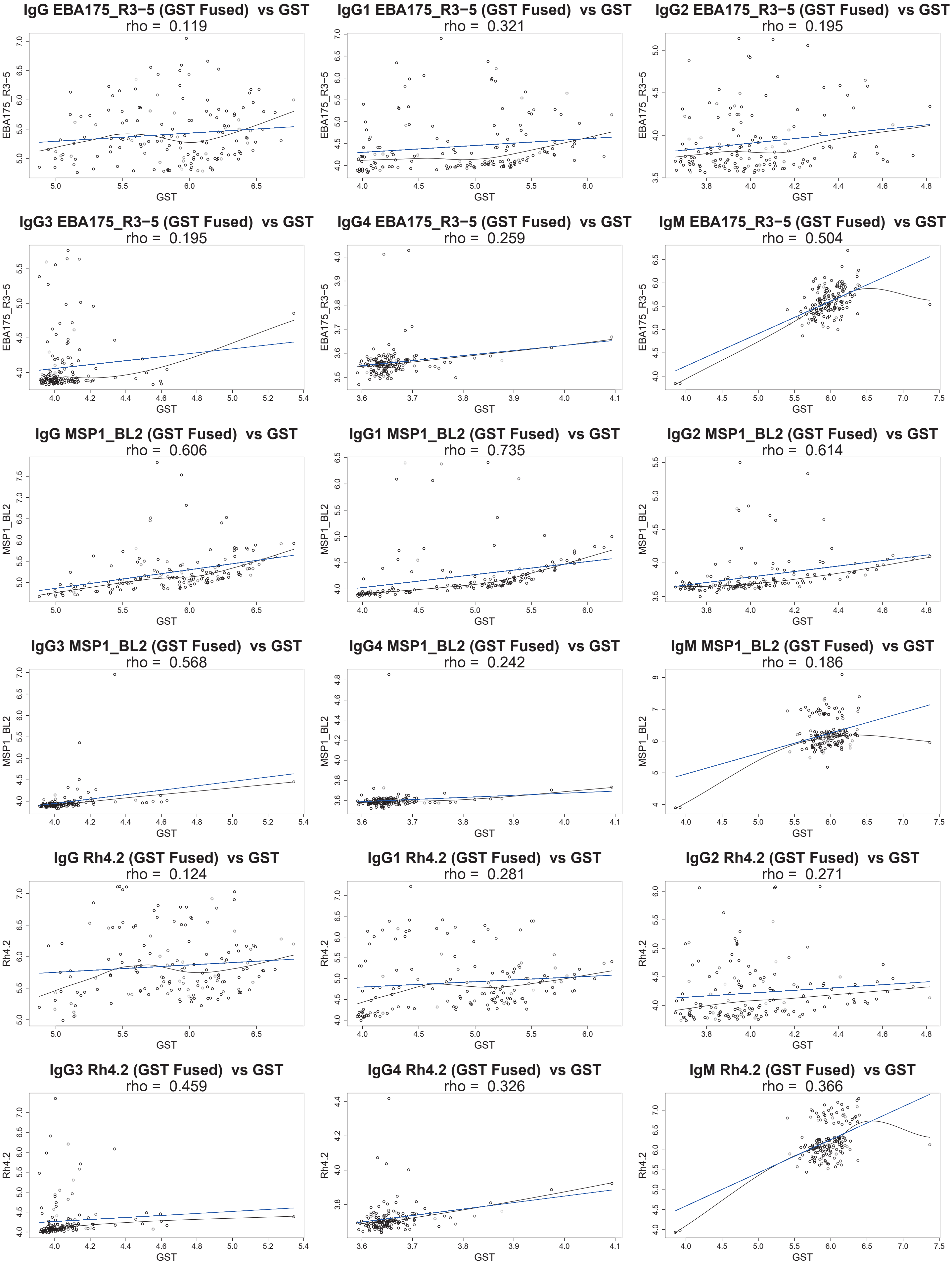

Supplementary Figure 42. Correlation plots for for antibody response to IgG, IgG1-4, IgM against GST fused antigens (EBA175_R3-5, MSP1_BL2, Rh4.2) vs GST: linear regression (blue line), LOESS curve fitting (black line).

Supplementary References

1. The RTS S Clinical Trials Partnership. Efficacy and safety of RTS,S/AS01 malaria vaccine with or without a booster dose in infants and children in Africa: Final results of a phase 3, individually randomised, controlled trial. *Lancet* **386,** 31–45 (2015).

2. The RTS S Clinical Trials Partnership. First Results of Phase 3 Trial of RTS,S/AS01 Malaria Vaccine in African Children. *N. Engl. J. Med.* **365,** 1863–1875 (2011).

3. González, R. *et al.* HIV Incidence and Spatial Clustering in a Rural Area of Southern Mozambique. *PLoS One* **10,** e0132053–e0132053 (2015).

4. Kolodny, N. *et al.* Two-step chromatographic purification of recombinant Plasmodium falciparum circumsporozoite protein from Escherichia coli. *J. Chromatogr. B Biomed. Sci. Appl.* **762,** 77–86 (2001).

5. Ubillos, I. *et al.* Baseline exposure, antibody subclass, and hepatitis B response differentially affect malaria protective immunity following RTS,S/AS01E vaccination in African children. *BMC Med.* **16,** 197 (2018).

6. Kastenmuller, K. *et al.* Full-length Plasmodium falciparum circumsporozoite protein administered with long-chain poly(I.C) or the Toll-like receptor 4 agonist glucopyranosyl lipid adjuvant-stable emulsion elicits potent antibody and CD4+ T cell immunity and protection in mice. *Infect. Immun.* **81,** 789–800 (2013).

7. Angov, E., Hillier, C. J., Kincaid, R. L. & Lyon, J. A. Heterologous protein expression is enhanced by harmonizing the codon usage frequencies of the target gene with those of the expression host. *PLoS One* **3,** e2189–e2189 (2008).

8. Reddy, K. S. *et al.* Bacterially expressed full-length recombinant Plasmodium falciparum RH5 protein binds erythrocytes and elicits potent strain-transcending parasite-neutralizing antibodies. *Infect. Immun.* **82,** 152–164 (2014).

9. Richards, J. S. *et al.* Association between naturally acquired antibodies to erythrocyte-binding antigens of Plasmodium falciparum and protection from malaria and high-density parasitemia. *Clin. Infect. Dis.* **51,** e50–60 (2010).

10. Black, C. G., Barnwell, J. W., Huber, C. S., Galinski, M. R. & Coppel, R. L. The Plasmodium vivax homologues of merozoite surface proteins 4 and 5 from Plasmodium falciparum are expressed at different locations in the merozoite. *Mol. Biochem. Parasitol.* **120,** 215–224 (2002).

11. Reiling, L. *et al.* The Plasmodium falciparum erythrocyte invasion ligand Pfrh4 as a target of functional and protective human antibodies against malaria. *PLoS One* **7,** e45253–e45253 (2012).

12. Cavanagh, D. R. & McBride, J. S. Antigenicity of recombinant proteins derived from Plasmodium falciparum merozoite surface protein 1. *Mol. Biochem. Parasitol.* **85,** 197–211 (1997).
